# Microbial Indicators of Dental Health, Dysbiosis, and Early Childhood Caries

**DOI:** 10.1177/00220345231160756

**Published:** 2023-04-11

**Authors:** D. Kahharova, V.Y. Pappalardo, M.J. Buijs, R.X. de Menezes, M. Peters, R. Jackson, A.T. Hara, G. Eckert, B. Katz, M.A. Keels, S.M. Levy, E. Zaura, B.W. Brandt, M. Fontana

**Affiliations:** 1 Department of Preventive Dentistry, Academic Centre for Dentistry Amsterdam (ACTA), University of Amsterdam and Vrije Universiteit Amsterdam, Amsterdam, the Netherlands; 2 Biostatistics Centre, Department of Psychosocial Research and Epidemiology, Netherlands Cancer Institute, Amsterdam, The Netherlands; 3 Department of Cariology, Restorative Sciences and Endodontics, University of Michigan, Ann Arbor, MI, USA; 4 Department of Cariology, Operative Dentistry and Dental Public Health, Indiana University, Indianapolis, IN, USA; 5 Department of Biostatistics and Health Data Science, Indiana University, Indianapolis, IN, USA; 6 Department of Pediatrics, Duke University School of Medicine, Durham, NC, USA; 7 Department of Preventive and Community Dentistry, College of Dentistry and Department of Epidemiology, College of Public Health, University of Iowa, Iowa City, IA, USA

**Keywords:** saliva, dental plaque, dental caries, child, 16S rRNA, antibiotics

## Abstract

Dental caries lesions are a clinical manifestation of disease, preceded by microbial dysbiosis, which is poorly characterized and thought to be associated with saccharolytic taxa. Here, we assessed the associations between the oral microbiome of children and various caries risk factors such as demographics and behavioral and clinical data across early childhood and characterized over time the salivary and dental plaque microbiome of children before clinical diagnosis of caries lesions. Children (*N* = 266) were examined clinically at ~1, 2.5, 4, and 6.5 y of age. The microbiome samples were collected at 1, 2.5, and 4 y. Caries groups consisted of children who remained caries free (International Caries Detection and Assessment System [ICDAS] = 0) at all time points (CFAT) (*n* = 50); children diagnosed with caries (ICDAS ≥ 1) at 6.5 y (C6.5), 4 y (C4), or 2.5 y of age (C2.5); and children with early caries or advanced caries lesions at specific time points. Microbial community analyses were performed on zero-radius operational taxonomic units (zOTUs) obtained from V4 of 16S ribosomal RNA gene amplicon sequences. The oral microbiome of the children was affected by various factors, including antibiotic use, demographics, and dietary habits of the children and their caregivers. At all time points, various risk factors explained more of the variation in the dental plaque microbiome than in saliva. At 1 y, composition of saliva of the C4 group differed from that of the CFAT group, while at 2.5 y, this difference was observed only in plaque. At 4 y, multiple salivary and plaque zOTUs of genera *Prevotella* and *Leptotrichia* were significantly higher in samples of the C6.5 group than those of the CFAT group. In conclusion, up to 3 y prior to clinical caries detection, the oral microbial communities were already in a state of dysbiosis that was dominated by proteolytic taxa. Plaque discriminated dysbiotic oral ecosystems from healthy ones better than saliva.

## Introduction

The oral microbiome establishes, matures, and changes throughout life in response to various physiological and environmental factors (Kilian et al. 2016). To ensure that the oral ecosystem remains healthy, it needs to adapt to changes occurring in the oral environment (Zaura and ten Cate 2015; Marsh and Zaura 2017). When the natural equilibrium between the host and its oral microbiome shifts toward an imbalanced, also called dysbiotic, state, this can promote demineralization of a tooth surface and increase risk of dental caries (Kilian 2018). A dysbiotic shift in the oral microbiome toward a cariogenic state commences before the manifestation of a caries lesion and is, therefore, challenging to determine and characterize.

Here we used a longitudinal design to assess associations between children’s oral microbiome and various caries risk factors, such as demographics, oral health, diet, and the behaviors of the children and their caregivers. At different time points (study visits), we also compared the salivary and dental plaque microbiome of groups of children with different caries statuses: those who remained caries free at all time points (for the entire study period from 1–6.5 y of age); those who were diagnosed with dental caries at 6.5 y, 4 y, or 2.5 y of age but remained caries free before that time point (i.e., those with ecological dysbiosis toward caries before its clinical manifestation); and those who presented with early and advanced caries lesions at a specific time point (i.e., at 2.5 y and 4 y of age).

## Materials and Methods

### Current Study Population

This report conforms to the Strengthening the Reporting of Observational Studies in Epidemiology (STROBE) guidelines. The study was part of a larger project, “Predicting Caries Risk in Underserved Toddlers in Primary Healthcare Settings” (Fontana et al. 2019). The current study is a longitudinal prospective cohort observational study. No intervention was performed during the study. The participants and study procedures have been described in detail elsewhere (Kahharova et al. 2020). Briefly, 266 children who completed 3 study visits (baseline or time point T1: ~1 y, T2: ~2.5 y, and T3: ~4 y of age) were included in the current study. At the age of 6.5 y (T4), 189 from 266 children underwent follow-up clinical oral examinations. Clinical oral examinations, the questionnaire administrations (Appendix Table 1), and the collection of the saliva and pooled dental plaque samples of children were performed at 3 time points (T1–T3). At baseline, saliva was collected from caregivers. Clinical oral examinations were performed using the International Caries Detection and Assessment System (ICDAS) criteria (Banting et al. 2012) by yearly calibrated examiners.

### Sample Collection and Processing

Sample collection, storage, DNA isolation, polymerase chain reaction (PCR) amplification, sequencing, and data processing were performed as described previously (Kahharova et al. 2020). The zero-radius operational taxonomic units (zOTUs) were assigned taxonomy using HOMD v14.51 (Chen et al. 2010). The sequences are available in the NCBI BioProject database under accession number PRJNA803343.

### Statistical Analyses

**Figure 1.**
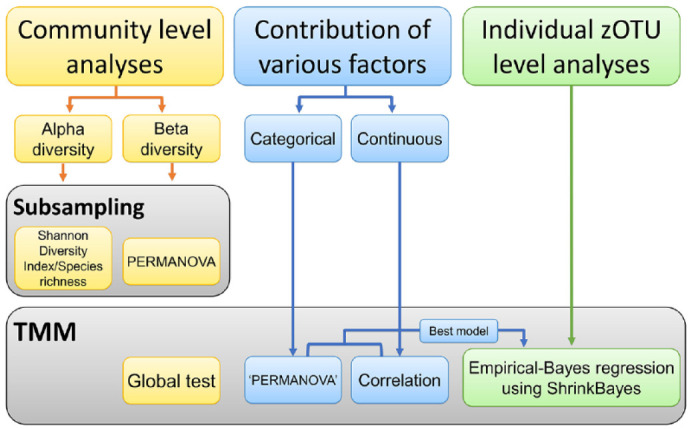
The flow diagram with the type of data set normalization per analysis type. Community-level analyses (yellow rectangles) were performed in both subsampled and trimmed mean of M-value (TMM) normalized data sets. Contribution of the various caries risk factors to the oral microbiome (blue rectangles) and individual zero-radius operational taxonomic unit–level analyses (green rectangles) were done on TMM normalized data sets.

Detailed analyses are described in the Appendix. In this study, the data set was normalized in one of the following 2 ways: by random subsampling of the zOTU table or by trimmed mean of M-value (TMM) normalization of the zOTU data (Fig. 1). In addition, we analyzed the contributions of various caries risk factors (variables) to the salivary and dental plaque microbiome over time and associations of those factors with the children’s caries status (Fig. 1). Principal component analysis (PCA), group comparisons (β-diversity) using PERMANOVA, the Shannon Diversity Index, and species richness calculations were performed in PAST software version 3.21 (Hammer et al. 2001). The *P* values were corrected for multiple testing (Bonferroni correction), with 0.05 as the overall significance level.

To assess the contribution of the risk factors (variables) to the microbiome variance over time, the envfit function (vegan version 2.5_7 [Oksanen et al. 2020], R version 4.1.3 [R Core Team 2022]) was performed. The variables that yielded the best model by the bioenv function were included in the empirical Bayes regression analysis using ShrinkBayes (version 2.13.7 [van de Wiel et al. 2014], INLA version 22.5.03 [Rue et al. 2009]) as covariates. zOTUs with a Bayesian false discovery rate (BFDR) (Storey 2003) of at most 0.1 were deemed significant. The global test (Goeman et al. 2004) (R package version 5.42.0, R version 4.02) was used to assess the relationship between the microbiome and a response variable (caries status). For categorical variables, multinomial univariate logistic regression was conducted, while for continuous variables, the Kruskal–Wallis and the Mann–Whitney tests were performed (Bonferroni corrected), using IBM SPSS version 25 (SPSS, Inc.).

## Results

### Caries Status over Time

**Figure 2.**
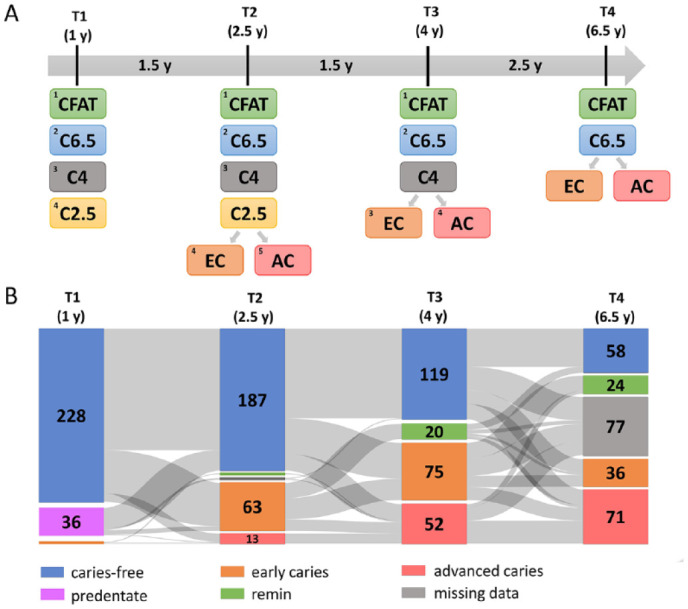
Distribution of the children based on the time of caries diagnosis and caries severity between 1 and 6.5 y of age. (**A**) The study groups according to the caries status per time point. The gray horizontal arrow shows the time interval in years between each time point. At T1 (age 1 y), children were divided into 4 groups by their caries status: 1) CFAT group (*n* = 50, green rectangle), children who remained caries free (International Caries Detection and Assessment System [ICDAS] = 0) at all time points (T1–T4, thus between age 1 and 6.5 y); 2) C6.5 group (*n* *=* 38, blue rectangle), children first time diagnosed with caries (ICDAS ≥ 1) at T4 (thus at 6.5 y) but caries free at T1, T2, and T3 (age 1, 2.5, and 4 y); 3) C4 group (*n* *=* 70, gray rectangle), children first time diagnosed with caries at T3 (4 y) but caries free at T1 and T2; and 4) C2.5 group (*n* *=* 75, yellow rectangle), children first time diagnosed with caries at T2 (2.5 y) but caries free at T1. At T2 (age 2.5 y), the children were divided into 5 caries groups: 1) CFAT (*n* = 50); 2) C6.5 (*n* *=* 38); 3) C4 (*n* *=* 70); 4) EC (*n* = 63, orange rectangle), children with early (thus presented with ICDAS = 1 or ICDAS = 2) caries lesions at T2 (2.5 y); and 5) AC group (*n* = 13, red rectangle), children with advanced (ICDAS ≥ 3) caries lesions at T2. At T3 (age 4 y), the children were divided into 4 caries groups: 1) CFAT (*n* = 50); 2) C6.5 (*n* = 38); 3) EC (*n* = 75), children with early caries lesions at T3 (4 y); and 4) AC (*n* = 52), children with advanced caries lesions at T3. (**B**) The Sankey graph of the entire study population. Among the 266 participants at the first visit (T1), 228 (85.7%) children were clinically caries free (blue columns), 2 children (0.9%) had early dental caries lesions (orange column), and 36 (13.5%) children were predentate (purple column). Only 50 (26.4%) children remained caries free during all 4 visits (T1–T4), while at T4, data were not available (missing data, gray column) for 77 (28.9%) children. Remin (green column)—children previously diagnosed with caries at the specific visit but caries free at the next time point, suggesting remineralization of lesions.

The children were assigned to different groups according to their caries status at each time point (Fig. 2A). Of the 266 children with microbial samples, 77 were lost to follow-up after 4 y of age (T3) (Fig. 2B). Across all time points (T1–T4 or 1–6.5 y of age), 50 of the 189 remaining children stayed clinically caries free Decayed, Missing due to caries, and Filled Teeth (dmft) = 0, d scored as ICDAS = 0 on all visible tooth surfaces) and were deemed the caries free at all time points (CFAT) group (Fig. 2A). While prevalence of dental caries (dmft ≥ 1; d scored as ICDAS ≥ 1) at the age of 1 y (T1) was 0.9% (*n* = 2), it was 28.8% (*n* = 76) at 2.5 y (T2) and 47.7% (*n* = 127) at 4 y (T3) (Fig. 2B). At 6.5 y (T4), 56.6% (*n* = 107) of the 189 children examined had at least 1 dental surface with ICDAS ≥ 1 (Appendix Fig. 1A, B). Prevalence of advanced caries (ICDAS ≥ 3) was 0% at T1, 4.9% at T2, 19.5% at T3, and 37.6% at T4. The median proportion of the teeth affected by caries (ICDAS ≥ 1) was 10% (range: 5–70%) at 2.5 y, 15% (5%–100%) at 4 y, and 16.7% (4%–67%) at 6.5 y.

### Oral Microbiome Analyses

#### Contributions of various caries risk factors to the oral microbiome composition

**Figure 3.**
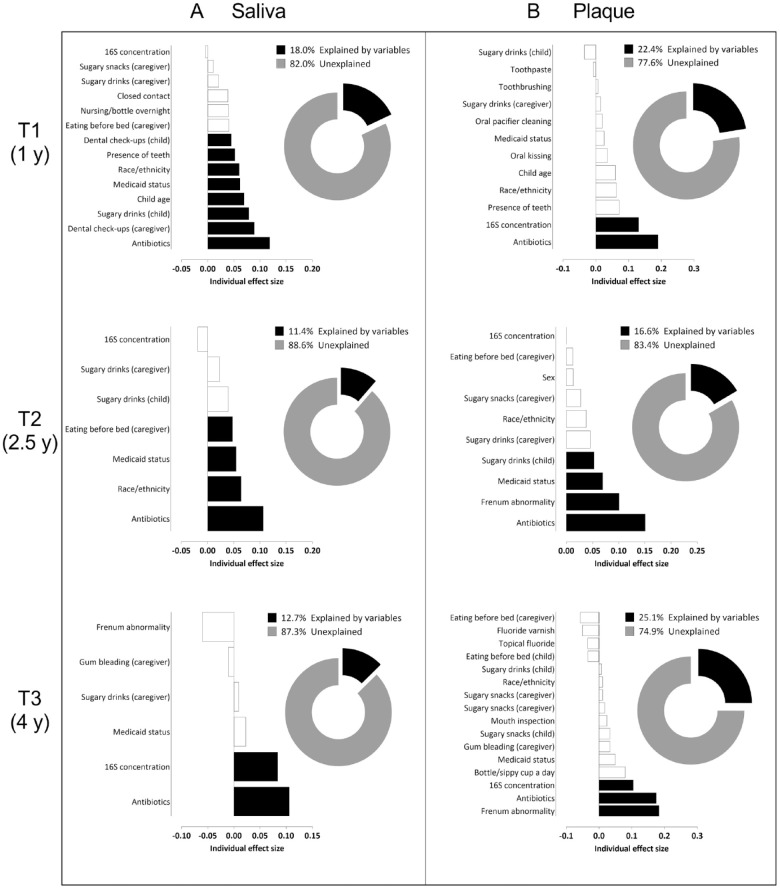
Variables associated with the salivary (**A**) and dental plaque (**B**) microbiome variation at T1, T2, and T3. Bar graphs depict the individual effect sizes of the variables selected as significantly influencing the microbiome composition. The black bars indicate the variables contributing to the best model based on the bioenv function. Bars with negative values mean that, even if the variable had a significant effect on the microbiome, it did not explain variation between samples. A variable can have a negative effect size if it is taken alone but contributes positively to the variation in combination with other variables. Pie charts show the percentage (%) of the explained variation of the microbiome composition with a combined effect size coming from the best combination of the selected variables (best model) (black part) and unexplained part of the microbiome (gray part).

All analyzed variables are listed in Appendix Table 1, and a detailed description of the contributions of selected variables to the oral microbiome of the children over time is provided in the Appendix. In brief, at T1, exposure to antibiotics explained the largest variation in the salivary microbiome (Fig. 3A). At each time point, various factors, including demographics and the dietary habits of the children and their caregivers, were significantly related to the salivary microbiome community variation (Fig. 3A). Between the baseline (T1) and the later time points (T2 and T3), the proportion of the variation in salivary microbial composition explained by the included variables decreased (T1: 18.0%; T2: 11.4%; T3: 12.7%). For dental plaque, the largest variation in microbiome composition was explained by exposure to antibiotics, thick or tethered maxillary labial frenulum, frequency of sugary drinks and snacks, concentration of the bacterial DNA in the sample, race/ethnicity, and Medicaid status (Fig. 3B). At all time points, the frequency of sugary drink intake by both the children and their caregivers was significantly related to the dental plaque microbiome community variation of the children (Fig. 3B). In contrast with saliva, at T3, the proportion of the variation in plaque microbial composition explained by the variables was the highest (T1: 22.4%; T2: 16.6%; T3: 25.1%). At all time points, explained variation in plaque composition was higher than that of saliva (Fig. 3).

#### Microbial community-level differences

**Figure 4.**
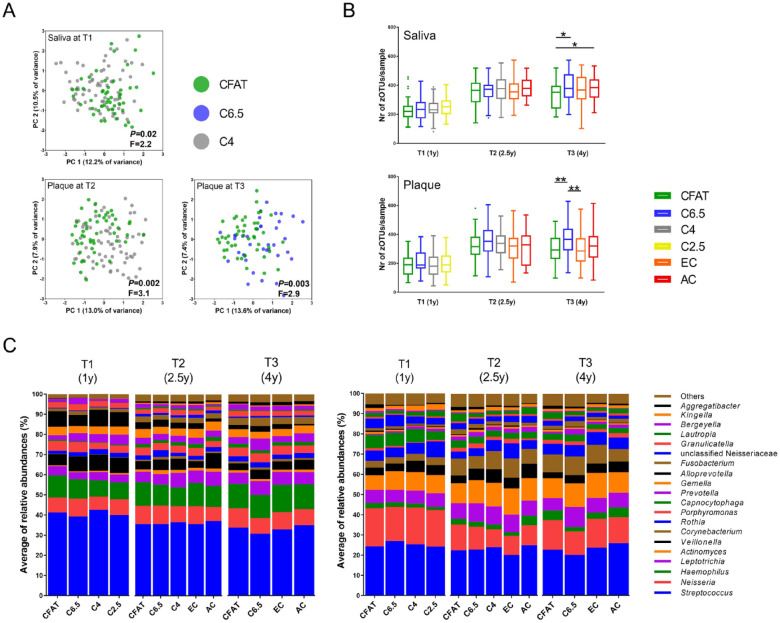
Microbial profile analyses of the salivary and dental plaque microbiome of the children over time (T1, T2, T3). (**A**) Principal component analysis (PCA) plots displaying the microbial profiles of the samples in the caries free at all time points (CFAT), C6.5, and C4 groups. Axes show the first 2 principal components (PCs) explaining the largest intersample variation (percentage of variance). The *P* and *F* values indicate the output of PERMANOVA analyses, using Bray–Curtis similarity. The *P* values were corrected for multiple testing using Bonferroni correction. (**B**) The α-diversity presented as species richness of the microbial composition in both salivary and dental plaque samples of the children according to their caries statuses over time. The boxplots are plotted using Tukey’s method. Statistically significant differences within the respective caries group are indicated by asterisks: *P < 0.05 and **P < 0.01 (Kruskal–Wallis test and Mann–Whitney test with Bonferroni correction). Different colors of the boxes indicate caries groups. Green boxes, CFAT (*n* = 50); blue, caries at 6.5 y (C6.5) (*n* = 38); gray, caries at 4 y (C4) (*n* = 70); yellow, caries at 2.5 y (C2.5) (*n* = 75); orange, early caries (EC) (International Caries Detection and Assessment System [ICDAS] score 1 and 2) (T2: *n* = 63; T3: *n* = 75); and red, advanced caries (AC) (ICDAS ≥ 3) (T2: *n* = 13; T3: *n* = 52) at the respective clinical examination. Lines connect the caries groups that differed within a time point. (**C**) Taxonomic distribution of the mean relative abundance of reads of the top 20 most abundant bacterial genera in salivary (left plot) and dental plaque (right plot) samples of the children according to their caries groups over time.

For the analyses below, detailed statistical outcomes are presented in the Appendix. In brief, at T1, the microbial profiles of saliva of the children, but not of plaque, differed significantly according to their caries status (Appendix Fig. 2A, B). The saliva of the 1-y-olds who would be diagnosed with caries by age 4 (C4) already differed from the saliva of those who remained clinically caries free throughout the study (CFAT) (Fig. 4A; Appendix Table 2A).

At T2, the microbial profiles of dental plaque, but not of saliva, differed significantly according to the caries groups (Appendix Fig. 2A, B). The dental plaque microbiome of the C4 group differed significantly from those in the CFAT group (Fig. 4A; Appendix Table 2B). The plaque in the CFAT group was distinct from that of the children with early caries lesions at 2.5 y (EC) (Appendix Table 2C). In other words, at the age of 2.5, dental plaque of not only those children who presented with early caries at the age of 2.5 but also those who were diagnosed with caries at the next time point (at the age of 4) differed compositionally from dental plaque of the children who remained caries free throughout the study.

**Figure 5.**
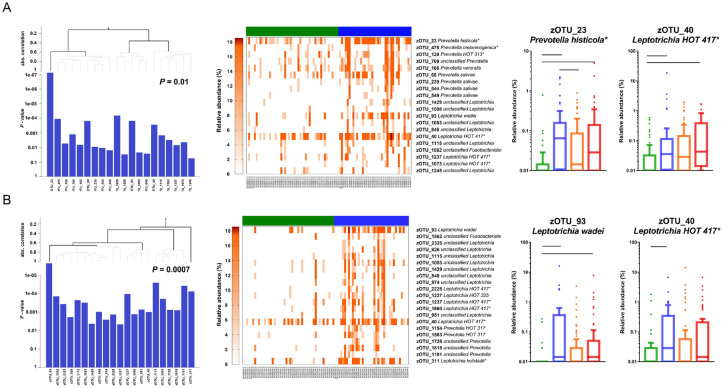
Differences in microbial composition between children with different caries status. The output of the global test comparing the composition of (**A**) salivary and (**B**) dental plaque samples collected at 4 y of age (T3) in children who remained caries free at all time points (CFAT) (*n* = 50) with those who were diagnosed with caries at 6.5 y (C6.5) (*n* = 38) but were caries free at the time of the sample collection (4 y). The bar graphs with dendrograms show the zero-radius operational taxonomic units (zOTUs) selected with the global test (*P* is the overall *P* value) and contributing to the significant differences in (A) salivary and (B) dental plaque samples. The dendrogram at the top shows single zOTUs and zOTU groups associated with the caries groups (in this case, all associated with C6.5, blue), with the absolute correlation from the global test at the upper y-axis. The lower y-axis shows the logarithmic *P* values corresponding to the tests for the associations of each individual zOTU with the caries group, plotted such that the longest bar has the lowest *P* value. The heatmaps show the relative abundance of the same zOTUs and zOTU groups in the same order as in the bar graphs in (A) salivary and (B) dental plaque samples in the CFAT (green) and C6.5 (blue) groups. The boxplots show examples of zOTUs selected with the global test in (A) salivary and dental (B) plaque samples by caries group: the CFAT, green; C6.5, blue; early caries (EC), orange; and advanced caries (AC), red. Lines connect the statistically significantly different groups. *Indicates zOTUs additionally blasted on the HOMD website with similarity ≥98.5%. The number of zOTUs per group, taxonomic names, and individual *P* values of all selected zOTUs are listed in Appendix Table 3A.

At T3, both the salivary and dental plaque microbiome profiles differed according to the caries groups (Appendix Fig. 2A, B). The saliva and plaque in the CFAT group were distinct from saliva and plaque in the C6.5 group (Fig. 5A, B; Appendix Table 3A, B) and in the EC (Appendix Table 3C) and AC groups (Appendix Fig. 3A, B; Appendix Table 3D, E). The dental plaque microbiome composition in the C6.5 group was distinct from that in the EC (Appendix Table 3F) and AC groups.

At T1 and T2, neither the salivary or plaque microbiome differed in α-diversity according to the caries groups, while at T3, the saliva samples in the CFAT group had significantly lower species richness than those in the C6.5 and AC groups (Fig. 4B). In plaque at T3, the microbial diversity was significantly higher in samples in the C6.5 group than in those in the CFAT group and in the EC group (Fig. 4B).

At the age of 4 (T3), in both saliva and plaque, groups of various species of the genera *Prevotella* and *Leptotrichia* contributed to the differences observed between the C6.5 and the CFAT groups, with a higher proportion of these taxa found in the C6.5 group (Fig. 5A, B; Appendix Table 3A, B). A group of taxa (zOTUs) assigned to the genera *Selenomonas*, *Streptococcus*, *Veillonella, Fusobacterium*, *Capnocytophaga*, *Propionibacterium*, and *Neisseria* was found at a higher proportion in the dental plaque of the C6.5 group than in that of the EC group (Appendix Table 3F).

#### Differences in individual taxa

First, we compared the top 20 most abundant genera in the salivary and dental plaque samples according to caries status (Fig. 4C). In saliva at T1, the relative abundance of genera *Actinomyces* and *Prevotella* increased and the proportion of *Porphyromonas* decreased from CFAT to C6.5, C4, and C2.5 (Appendix Fig. 4A), while no differences were observed at T2 and T3. In dental plaque, no significant differences by caries status were observed at T1, while at the later time points, the relative abundance of *Lautropia*, *Bergeyella*, and *Aggregatibacter* decreased with caries progression, and *Prevotella* and *Fusobacterium* were higher in children who developed caries by the next time point (Appendix Fig. 4B).

Next, we assessed the contribution of individual zOTUs to differences among the caries groups. For this, we used ShrinkBayes regression analysis, which included the most significant factors (best model) as covariates (Fig. 1). For saliva collected at T1, the following covariates were selected as the best model: child’s age, exposure to antibiotics, race/ethnicity, Medicaid status, number of teeth, the frequency of the child’s consumption of sugary drinks between meals, and the frequency of dental visits of the children and their caregivers. In total, 6 zOTUs differentiated between the C4 group and the CFAT group (Appendix Table 4A). The microbial profiles of dental plaque at T1 and those of saliva at T2 did not differ significantly according to the caries groups; therefore, the analyses on individual zOTUs were not performed. For T2 dental plaque, 4 covariates resulted in the best model: exposure to antibiotics, Medicaid status, tight or tethered maxillary labial frenulum, and frequency of the child’s consumption of sugary drinks between meals. Several zOTUs were significantly discriminatory between the dental plaque in the CFAT and samples from the C4 and EC groups (Appendix Table 4B, C).

For T3 saliva, exposure to antibiotics and the bacterial DNA concentration were included as covariates. Numerous zOTUs discriminated among the caries groups (Appendix Table 5A–C). For example, zOTUs such as zOTU_22 unclassified *Kingella/Neisseria*, zOTU_23 *Prevotella histicola*, and zOTU_34 *Streptococcus gordonii/HOT 056* significantly discriminated the saliva of the C6.5 group from that of the CFAT group (Appendix Fig. 5A). For the T3 dental plaque, 3 covariates were selected as the best model: exposure to antibiotics, tight or tethered maxillary labial frenulum, and bacterial DNA concentration in the sample. Among the taxa analyzed, 101 zOTUs significantly discriminated between the plaque in the C6.5 and CFAT groups, 95 zOTUs of which were at a higher proportion in the C6.5 group (Appendix Table 6A). For example, several zOTUs of *Prevotella* (zOTU_23 *P. histicola*, zOTU_68 *P. salivae*, zOTU_77 *P. nigrescens*) and *Leptotrichia* (zOTU_40 *L. HOT 417*, zOTU_93 *L. wadei*, zOTU_210 *L. HOT 212*) were present at a significantly higher proportion in the plaque of the C6.5 group (Appendix Fig. 5C). The detailed results are shown in the Appendix (Appendix Fig. 5; Appendix Tables 5 and 6).

#### Demographics, dietary and oral hygiene habits, and caries status

Child age, sex, delivery mode, and exposure to antibiotics did not differ across caries groups. However, Medicaid status and the recruitment site for all time points, income category for T2 and T3, and race/ethnicity for T3 did differ (results not shown) among the caries groups. The frequencies of consumption of sugary drinks by the children themselves and by their caregivers were significantly associated with the caries status at all time points (Appendix Fig. 7A, B). In addition, at T2, the frequency of the caregivers’ consumption of sugary snacks and caregivers’ consumption of anything other than plain water before going to bed was significantly associated with the caries status of their children. The frequency of caregivers’ inspection of the dentition of their child was significantly associated with caries status at T2 and T3.

## Discussion

This study showed that the microbiome of children with clinically sound teeth, but imbalanced (dysbiotic) oral microbiome toward caries, differed from the microbiome of those who remained caries free at all time points and those who had clinically established early and advanced caries lesions. The oral microbiome of the children was affected by various factors such as antibiotic use, maxillary labial frenulum abnormality, bacterial and fungal DNA concentration in the samples, demographics, Medicaid status, and the eating and oral hygiene habits of the children and their caregivers.

We found that, 1 to 3 y prior to clinical caries detection, oral microbial communities were in a state of dysbiosis that was dominated by proteolytic taxa such as *Leptotrichia* and *Prevotella* and were more diverse than communities in children who stayed caries free at all time points. The higher bacterial diversity and the higher abundance of the genera *Leptotrichia* and *Prevotella* have been associated with plaque accumulation and gingival inflammation (Teng et al. 2016). These findings, however, differ from the caries-associated microbial shifts proposed in the ecological plaque hypothesis (Marsh 1994). This hypothesis is based on the notion that dysbiosis occurs after frequent exposure to sucrose, which leads to the enrichment of aciduric and acidogenic microbiota and the loss of bacterial diversity at the expense of acid-sensitive microorganisms (Marsh 1994; Marsh 2003). This may be true for the very late stages coinciding with the commencement of the lesion formation, as has been shown in studies with initial caries lesions (Gross et al. 2012; Peterson et al. 2013). Our findings indicate that a carbohydrate-rich oral environment, possibly combined with neglected oral hygiene, promotes a bacterial shift toward highly diverse and metabolically versatile communities. Although we did not assess gingival health and the amount of plaque, the significant role of bacterial DNA concentration in microbiome composition of dental plaque and our microbiological findings suggest poor oral hygiene in a part of our study population. To verify this assumption, future studies should assess oral hygiene and gingivitis and perform microbial sampling at more frequent intervals.

One of the unexpected findings of our study was the resilience toward caries development (Rosier et al. 2017): even though the salivary or dental plaque microbiome presented signs of dysbiosis, some children were not diagnosed with caries during the second checkup at the age of 2.5 (1.5 y after the first assessment at the age of 1), but only at the third assessment at the age of 4. The interplay among microbial and host-related factors, such as the immune system (Zaura et al. 2014) and salivary properties (Pedersen et al. 2018), might play a role and should be included in future assessments.

Another unusual finding was the incongruity of the results in the 2.5-y-olds relative to those from the same children at a younger or an older age. At T2, when the children were approximately 2.5 y old, no significant differences in salivary microbiome across caries groups were observed, while saliva in both the 1- and 4.5-y-olds did distinguish children who remained caries free at all time points from the other groups. In addition, plaque samples collected from children with dysbiosis or caries at T2 had higher proportions of *S. mutans* than did samples from the same children at T1 or T3. Taken together, these findings suggest that being a toddler is a unique stage not only in general terms but also for a person’s oral ecosystem, which is influenced by environmental factors such as having a fully erupted deciduous dentition and lack of parenting control over a child’s behavior (Campbell et al. 2000). To elucidate this, future studies should combine assessment of the oral ecosystem of children with assessment of their parents’ behaviors and parenting control (Quinonez et al. 2020). In the youngest age group (12 mo), saliva appeared to be more discriminatory among caries groups, most likely due to the low number of erupted teeth. However, in the 2.5- and 4-y age group, pooled dental plaque emerged as a more informative sample in discriminating among the caries groups than saliva.

In addition, currently assessed covariates could account only for a maximum of 18% of salivary or 25% of dental plaque microbiome composition variance, suggesting that a much broader view on the host genetic factors and immunological, environmental, and behavioral parameters might be needed to explain how the individual oral microbiome is shaped. The current study focused on questions that are associated with caries risk assessment only and can be self-administered to primary caregivers during medical or dental visits.

The current study sample exhibited high early and advanced caries prevalence, with high frequency of consumption of sweet drinks and snacks. Interestingly, in our study, not only the dietary habits of the children themselves but also those of their caregivers correlated positively with the caries outcome and affected the composition of the oral microbiome of the children, most likely due to higher availability of sugary snacks in certain households (Vepsäläinen et al. 2018).

## Conclusion

Our findings demonstrate that the shift in the balance of the oral microbiome occurs before the clinical manifestation of dental caries. The dysbiosis state is observed 1 to 3 y before clinical detection of caries lesions and is associated with an increase of anaerobic and proteolytic taxa like *Prevotella* and *Leptotrichia*. Dental plaque in children with complete deciduous dentitions discriminates a dysbiotic oral ecosystem from a healthy one better than does saliva. Our findings also show that various caries risk factors such as antibiotics, frenulum abnormality, demographics, Medicaid status, and dietary and oral hygiene habits affect the oral microbiome composition of children over time. At all time points, various factors explained a greater proportion of variance in the dental plaque microbiome than that of saliva.

## Author Contributions

D. Kahharova, V.Y. Pappalardo, M.J. Buijs, R.X. de Menezes, E. Zaura, B.W. Brandt, contributed to data acquisition, drafted and critically revised the manuscript; M. Peters, R. Jackson, A.T. Hara, G. Eckert, B. Katz, M.A. Keels, S.M. Levy, M. Fontana, contributed to conception and design, data acquisition, drafted and critically revised the manuscript. All authors gave final approval and agree to be accountable for all aspects of the work.

## Supplemental Material

sj-docx-1-jdr-10.1177_00220345231160756 – Supplemental material for Microbial Indicators of Dental Health, Dysbiosis, and Early Childhood Caries Supplemental material, sj-docx-1-jdr-10.1177_00220345231160756 for Microbial Indicators of Dental Health, Dysbiosis, and Early Childhood Caries by D. Kahharova, V.Y. Pappalardo, M.J. Buijs, R.X. de Menezes, M. Peters, R. Jackson, A.T. Hara, G. Eckert, B. Katz, M.A. Keels, S.M. Levy, E. Zaura, B.W. Brandt and M. Fontana in Journal of Dental Research
