# Supplementary material for: Microbial Indicators of Dental Health, Dysbiosis, and Early Childhood Caries
Source: J Dent Res. 2023 Apr 11;102(7):759–66. doi: 10.1177/00220345231160756 (PMC10288166; doi:10.1177/00220345231160756)
Supplement: sj-docx-1-jdr-10.1177_00220345231160756 – Supplemental material for Microbial Indicators of Dental Health, Dysbiosis, and Early Childhood Caries [file sj-docx-1-jdr-10.1177_00220345231160756.docx]

**MICROBIAL INDICATORS OF DENTAL HEALTH, DYSBIOSIS AND EARLY CHILDHOOD CARIES**

D. KAHHAROVA, V. Y. PAPPALARDO, M. J. BUIJS, R. X. DE MENEZES, M. PETERS, R. JACKSON, A. HARA, G. ECKERT, B. KATZ, M. A. KEELS, S. M. LEVY, E. ZAURA, B.W. BRANDT AND M. FONTANA

**APPENDIX**

**MATERIALS AND METHODS**

ETHICAL CONSENT AND STUDY DESIGN

Approval for this study was obtained from the Medical Ethics Committee of the University of Michigan Medical School [Institutional Review Board (IRBMED) approval no. HUM00071519. Date of approval: 14-03-2013], Duke University [Duke Medicine Institutional Review Board approval no. PRO00044905. Date of approval: 13-06-2013], Indiana University [Indiana University Institutional Review Board approval no. 1303010908. Date of approval: 15-04-2013] and University of Iowa [University of Iowa Institutional Review Board approval no. 201302810. Date of approval: 5-03-2013].

CURRENT STUDY POPULATION

In total, 266 children completed collection of the salivary and supragingival dental plaque samples at three study visits (time points) (T1: ~1-year-old, T2: ~2.5-year-old and T3: ~4-year-old). All children underwent clinical oral examinations. The caregivers completed the questionnaire at each time point. From the 266 children, 189 were followed up to T4 (~6.5 years of age) as part of the extension of the study. In the current study, we analyzed the data from the questionnaires, the clinical oral examinations, and the microbiome of the salivary and dental plaque samples of 266 children at the first three time points (T1–T3), and included information from the clinical oral examinations of the 189 children obtained at T4. We also assessed the salivary microbiome of the primary caregivers at baseline (T1).

CLINICAL ORAL EXAMINATION

The clinical oral examination has been described in detail elsewhere (Kahharova et al. 2020). In brief, the oral cavity of the children was examined by calibrated dentists at four time points (T1: ~1 y, T2: ~2.5 y, T3: ~4 y and T4 ~6.5 y of age). To determine the caries status of the children, the International Caries Detection and Assessment System (ICDAS II) criteria were used (Banting et al. 2012). The prevalence of dental caries was calculated based on the percentage of participants with both early and advanced caries (ICDAS≥1) and with advanced caries (ICDAS≥3) lesions. We also determined the median number of caries-affected surfaces per child for the children with both early and advanced caries lesions (ICDAS≥1) and for the children with advanced caries lesions (ICDAS≥3). To estimate the degree of severity of dental caries, we calculated the number of caries-affected teeth (including both early and advanced lesions) and divided them by the number of teeth present at each time point. During the clinical oral examination, presence of constricted tongue, abnormality of the maxillary labial frenulum, and spaces between teeth also was recorded. All participants received equal information on good oral health preventive practices at home at every visit, and were informed of the findings from the clinical oral examinations, with information on need for follow up care, and information on where to seek care if they did not have a dentist.

ENVIRONMENTAL AND QUESTIONNAIRE FACTORS

In total, 39 variables (28 from the DCR-007-Primary Caregiver Questionnaire (Daly et al. 2016; Eckert et al. 2010; Fontana et al. 2019; Fontana et al. 2011) and 11 from the oral examination, sample processing, sequencing, and general information) were included in the current analyses (Appendix Table 1). The questions included oral hygiene and eating habits, dental care regarding both the children and their primary caregivers, demographics and general information and health insurance concerning the children (sex, race/ethnicity, and delivery mode), and the behavior of the caregivers toward the oral and medical health and care of their children (Appendix Table 1). Due to the highly skewed distributions, the answers to the questions representing frequency of oral hygiene and dietary habits (twice a day, daily, weekly, monthly, and never) were combined into two categories (at least daily and less frequently). The responses to the question on the frequency of visiting a dentist (twice a year, yearly, only when pain and never) were also combined into two categories (at least yearly and never). The answers to the questions related to the behavior toward dental and medical health of the participants (excellent, very good, good, fair and poor) were combined into three (1) excellent or very good; 2) good; 3) fair or poor) or two (1) excellent or very good; 2) good, fair or poor) categories. Finally, race/ethnicity were combined into the Caucasian, Afro-American, Hispanic and multi-race groups.

SAMPLE COLLECTION AND PROCESSING

Sample collection, storage, DNA isolation, PCR amplification, sequencing, and data processing were performed as described previously (Kahharova et al. 2020). In brief, saliva and dental plaque samples were collected from children at three time points: at baseline or T1, when children were approximately 1-year-old, at T2 (2.5 years of age), and T3 (4 years of age). Unstimulated saliva was collected at T1 from all primary caregivers. Saliva collection from the caregivers was performed by drooling 1 mL saliva into the funnel (OM-505 tube) without active spiting. The saliva of the children was collected by gently swabbing the left pouch, the floor of the mouth, and the right pouch for 30 sec each with two sponges (Puritan PurFlock Ultra, Guilford, Maine, USA). Pooled dental plaque samples from children were collected before the ICDAS examination by swabbing vestibular surfaces of the teeth with a sterile microbrush (Microtip micro-applicator fine size; Microbrush International, Grafton, Wisconsin, USA). First choice to collect dental plaque was upper front teeth. If upper front teeth were not present, then the lower front teeth were sampled. After DNA extraction and purification, bacterial DNA concentration was determined by a 16S ribosomal RNA gene quantitative polymerase chain reaction (qPCR) with universal primers specific to the bacterial 16S rRNA gene (Ciric et al. 2010). Samples were normalized based on the 16S rRNA gene concentration by dilution to 200 pg/µl. The V4 hypervariable region of the 16S rRNA gene was amplified with barcoded forward and reverse primers (Kozich et al. 2013). Samples were mixed equimolarly; PCR products of isolation blanks, sample blanks, run-to-run controls, and negative PCRs were included and the final mix was sequenced (MiSeq; Illumina). The concentration of fungal DNA in the samples was determined using qPCR (Vollmer et al. 2008). The reads were denoised using UNOISE3 and mapped to the zero-radius operational taxonomic units (zOTUs). As described previously (Kahharova et al. 2020), the representative (most abundant) sequence of the zOTUs was assigned taxonomy using HOMD v14.51 (Chen et al. 2010) and the RDP classifier (Wang et al. 2007). zOTUs that were found statistically significant in the downstream analyses were additionally blasted on the HOMD website (using HOMD v14.51) and classified at the species level if the sequence similarity was ≥98.5%. Between 95% and 98.5% sequence similarity assignments were done at the genus or higher level. In both cases, the query sequence coverage in the alignment had to be ≥98%. The sequences are available in the NCBI BioProject database under the accession number PRJNA803343.

STATISTICAL ANALYSES

In this study, the dataset was normalized in one of the following two ways: by random subsampling of the zOTU-table or by trimmed mean of M-value (TMM) normalization of the zOTU data. The normalization method was chosen depending on the type of analysis used to approach the dataset on the microbial community level or the level of individual taxa (Fig. 1). All the figures illustrating the results from the oral microbiome analyses, except dendrograms, were plotted using the subsampled zOTU-table.

*Statistical analyses with subsampling*

For multivariate microbial profile analyses, the zOTU-table was subsampled at a depth of 7,000 reads per sample. Next, the zOTU-table was log-2 transformed (on read counts + 1) and ordinated using Principal Component Analysis (PCA). Differences in microbial profiles among the sample groups (β-diversity) were assessed with one-way Permutational Multivariate Analysis of Variance (PERMANOVA; 9999 permutations) using the Bray-Curtis similarity. α-diversity was assessed using the Shannon Diversity Index and species richness (number of zOTUs/sample). These analyses were performed using PAST software version 3.21 (Hammer et al. 2001). The *P*-values were corrected for multiple comparisons using Bonferroni correction, and 0.05 was used as the significance level unless otherwise stated. Additionally, for these multivariate microbial profile analyses, the global test (see below) was used.

*Statistical analyses with TMM normalization*

For these analyses, the original zOTU-table was filtered by excluding samples with less than 1000 reads. Then, the data were split into six sub-tables according to the time points (T1-T3) and the sample type (saliva or dental plaque). For the caries-group comparisons only, for each time point and sample type, spurious zOTUs (present in less than 20% of the samples in at least one of the caries groups) were removed from the zOTU-table. Next, normalizing factors were computed using the TMM method (Robinson and Oshlack 2010) implemented in the edgeR package (version 3.36.0 (Robinson et al. 2010)), the zOTU abundances were multiplied by their normalizing factors, rounded to keep an integer count table and transformed using the inverse hyperbolic sine (asinh) (Huber et al. 2002). This function becomes equivalent to the logarithmic transformation for values in the high range, while zeros are preserved. The resulting datasets were used to fit vectors or factors onto a PCoA ordination, for Empirical-Bayes regression analysis using ShrinkBayes (van de Wiel et al. 2014), and for analysis using the global test (Goeman et al. 2004), described below.

Three types of analyses were performed. First, we identified the contributions of various caries risk factors (continuous and categorical variables) to the variation in the microbiome (Falony et al. 2016) of the children’s saliva and dental plaque per time point. The associations between continuous or categorical variables and the PCoA ordination (on a Bray-Curtis distance matrix) were calculated using the envfit function (vegan package version 2.5_7 (Oksanen et al. 2020)) with 8 components and 999999 permutations. Returned *P*-values were corrected (false discovery rate, threshold *P*<0.05, (Benjamini and Hochberg 1995)). Then, the bioenv function (Spearman correlation, Gower distance, maximum eight variables) was used to select the combination of variables with highest correlation to the Bray-Curtis distances of the microbiome samples. To assess the individual effect size (correlation) of a specific variable, we also ran the bioenv function for each variable separately.

Second, based on the outcomes of the above analyses, variables which yielded the best model were included as covariates in Empirical-Bayes regression analyses using the ShrinkBayes R package version 2.13.7 (van de Wiel et al. 2014). These Empirical-Bayes regression analyses estimate the effects of covariates on a count variable as a response, which follows a negative binomial distribution with zero inflation. These analyses were used to identify the discriminatory zOTUs among different caries groups (CFAT, C6.5, C4, C2.5, EC, and AC) per sample type (the saliva or dental plaque) and time point (T1-T3). Those zOTUs with a Bayesian False discovery rate (BFDR) of at most 0.1 were declared statistically significant. BFDR is a method to correct a *P*-value for multiple testing (Storey 2003).

Third, to analyze the microbiome at the community level, we performed the global test (Goeman et al. 2004), which is a method that can be used to assess the association between a group of features (here zOTUs) and a response variable (here, the caries status). For this, we used the gt function from the globaltest R package (version 5.42.0) which returns a *P*-value that indicates how likely it is to encounter by chance the observed differences in the entire microbiome among samples with different caries statuses. Subsequently, using this package, sequential testing was performed to identify individual or subgroups of zOTUs that display the differences among samples with the different caries status (*P*<0.05). A significant subgroup of zOTUs can be interpreted as indicating that at least one of the zOTUs in the subgroup is significantly related to the given caries status. For these microbial profile analyses, both the global test and PERMANOVA were used. If one of these was significant, we reported both test results and regarded the difference significant.

*Univariate statistical tests*

For categorical variables, to assess differences in various risk factors (Appendix Table 1) of the children and their caregivers according to the children’s caries status, the multinomial univariate logistic regression test was applied. For continuous variables, the Kruskal-Wallis and the Mann-Whitney tests were performed. The above tests were performed using SPSS version 25. The Bonferroni correction was applied in cases of multiple testing.

**RESULTS**

CARIES STATUS OVER TIME

At T1 (~1-year-olds), only two participants (0.9%) experienced dental caries (dmft≥1; ICDAS≥1) (Fig. 2B). At T2 (~2.5-year-olds), caries prevalence was 28.8% (*n*=76), with median of 2 (range: 1-18) caries-affected (ICDAS≥1) surfaces per child among only those affected by caries (Appendix Fig. 1A). At T3 (~4-year-olds), caries prevalence was 47.7% (*n*=127) with median of 5 (range: 1-71) surfaces among only those affected by caries. At T4 (~6.5-year-olds), 56.6% (*n*=107) of 189 examined children had dental caries with median of 6 (range: 1-76) surfaces among only those affected by caries (Appendix Fig. 1A). For the children with advanced caries lesions (ICDAS≥3), caries prevalence and the median number (range) of caries-affected surfaces among only those affected also increased with time: T2 - caries prevalence was 4.9% (*n*=13) with median of 4 (1-11), T3 - 19.5% (*n*=52) with median of 5 (1-61), and at T4 - 37.6% (*n*=71) with median of 10 (1-76) (Appendix Fig. 1B). At T2, the median proportion of teeth affected by caries (ICDAS≥1) relative to the number of erupted teeth was 10% (range: 5-70%); at T3 - 15% (5-100%); and at T4 - 16.7% (4-67%). Several children experienced remineralization of caries lesions at each time point (*n=*1 at T2, *n*=20 at T3, *n*=24 at T4) (Fig. 2B). Two children at T2 and 77 children at T4 did not attend the dental examination which resulted in missing data (Fig. 2B). Loss of the participants in follow-up time point (T4) as part of the extension of the study was due to self-withdrawal due to time constraints to participate in the study and attend visits, moving or other reasons, or those who were withdrawn by the Study Principal Investigator either because ongoing contact was not obtained or if they developed any unanticipated problem and would not meet the inclusion criteria anymore (Daly et al. 2022). The data from children who had experienced presumably remineralization of initial caries lesions and the children with missing values were excluded from the microbiome analyses at the respective time points.

ORAL MICROBIOME ANALYSES

*Overall sequencing output*

Extended results of the sequencing output of the entire dataset before subsampling, data cleaning from the contamination, and the assessment of potential batch effects of sequencing were presented previously (Kahharova et al. 2020). The current dataset consisted of the salivary and dental plaque samples collected from the children at three time points. In total, 1,514 samples remained in the subsampled and 1,556 samples in TMM-normalized datasets. The 2,068 zOTUs of the subsampled dataset were classified into 11 phyla and 143 genera or higher-level taxa. Of all zOTUs, 22 zOTUs (1,725 reads or 0.016%) could only be assigned to kingdom Bacteria. From the caregivers, 251 baseline salivary samples were analyzed for identification of zOTUs classified as *Streptococcus mutans* and *Porphyromonas gingivalis*. The caregivers’ data subset (at T1 only) consisted of 2,176 zOTUs.

*Contributions of various caries risk factors to the oral microbiome composition*

We have tested the contributions of 39 metadata variables (demographics and general questions, variables related to sample collection, processing and sequencing, variables from the oral examination, questions about dietary, oral hygiene and behavioral habits of the children and their primary caregivers) (Appendix Table 1) to the microbiome variation in the salivary and dental plaque samples of 266 children over time. In one-year-olds (T1), 14 variables were significantly related to the salivary microbiome community variation. With 11.8% of individual effect size, exposure to antibiotics explained the largest variation in the salivary microbiome. The combination of the 8 variables such as exposure to antibiotics, child’s age in months, number of teeth present, race/ethnicity, Medicaid status, frequency of use of sugary drinks between meals by the child, and frequency of dental visits by the child and by the caregiver showed the best correlation (best model) to the salivary microbiome composition, with the combined effect size of these variables being 18.0% (Fig. 3A). At T2 (2.5 years of age), 7 variables and, at T3 (4 years of age), 6 variables were significantly related to the salivary microbiome. Additionally, at each time point, dietary habits such as frequency of sugary drinks between meals of the caregivers (Question 39) were related to the salivary microbiome community variation. The combined effect size of the best combination of variables was 11.4% at T2 and 12.7% at T3 (Fig. 3A).

In contrast to the saliva samples, the number of influencing variables on the dental plaque microbiome increased between the first two timepoints (T1 and T2) and the latest time point (T3) (Fig. 3B). The dental plaque microbiome of 1-year-old children (T1) was significantly related to 12 variables. Two variables showed the highest correlation, explaining 22.4% of the variation in the plaque microbiome at this age: concentration of the bacterial DNA of the samples and exposure to antibiotics (Fig. 3B). At T2, 10 variables contributed significantly to plaque microbiome variation. Five of these variables formed the best combination, explaining 16.6% of the variation. At T3, 16 variables, three of which constituted the best combination, were significantly related to dental plaque microbiome composition. The combined effect size of the best correlation variables at T3 was 25.1% (Fig. 3B), which had the largest explanatory power for the microbiome composition by the tested variables among all time points and sample types. For all time points, frequency of sugary drinks of the caregivers was significantly related to the dental plaque microbiome community variation of the children. For all time points, the variables of exposure to antibiotics, abnormality of the maxillary labial frenulum, frequency of sugary drinks and snacks by the children and the caregivers, concentration of bacterial DNA, race/ethnicity, and Medicaid status consistently appeared as the top individual features explaining the largest variation in the dental plaque microbiome. At all time points, the variation in plaque composition explained by various risk factors was higher than that explained for saliva (Fig. 3).

*Microbial community-level differences*

For the community-level analyses, we performed both PERMANOVA, with the subsampled dataset and the global test using the TMM-normalized dataset to assess the overall microbiome composition, species (zOTUs) similarity, diversity, and richness (Fig. 1). The description below is similar to the main text, but includes more detailed information and the statistical output of the tests used.

At the age of one year (T1), the microbial profiles of saliva differed significantly according to the children’s caries status by PERMANOVA (*P*=0.04, F=1.3), but not by the global test (*P*=0.052) (Appendix Fig. 2A, B). The microbiome of dental plaque at T1 did not differ by caries status. The saliva of the 1-year-olds who were diagnosed with caries three years later at 4 y of age (C4) already differed from the saliva of those who remained clinically caries-free throughout the study (CFAT) (*P*=0.02, F=2.2, PERMANOVA; *P*=0.03, global test; both Bonferroni-corrected) (Fig. 4A; Appendix Table 2A). Those with caries diagnosed after 1.5 years at 2.5 y of age (C2.5) also differed from those in the CFAT group (*P=*0.02, F=1.8, PERMANOVA; *P*=0.03, global test) based on the unadjusted *P*-values, though after Bonferroni correction both tests lost significance.

At the age of 2.5 years (T2), the microbial profiles of the dental plaque (*P*=0.0007, F=1.6, PERMANOVA; *P*=0.002, global test), but not of saliva, differed significantly according to the caries groups of these children (Appendix Fig. 2A, B). The dental plaque of the C4 group differed significantly from those in the CFAT group (*P*=0.003, F=3.1, PERMANOVA; *P*=0.0007 global test; Bonferroni-corrected) (Fig. 4A; Appendix Table 2B). The plaque in the CFAT group was distinct from that of the children with early caries (EC) lesions at 2.5 y (ICDAS 1 and 2) (*P*=0.01, F=2.4, PERMANOVA; *P*=0.02 global test; Bonferroni-corrected) (Appendix Table 2C) while it did just not significantly differ from the advanced caries (AC) lesions at 2.5 y (ICDAS>3) (*P*=0.082, F=1.9, PERMANOVA; *P*=0.053 global test; Bonferroni-corrected).

At the age of 4 years (T3), both the salivary (*P*=0.0002, F=1.8, PERMANOVA; *P*=0.00003, global test) and dental plaque (*P*=0.0001, F=2.3, PERMANOVA; *P*=0.00006, global test) microbiome profiles differed according to the caries groups (Appendix Fig. 2A, B). The saliva of the C6.5 group was distinct from the CFAT group (*P=*0.01 Bonferroni-corrected global test; *P=*0.014, F=1.9, PERMANOVA; with *P*=0.08 not significant after Bonferroni correction) (Fig. 5A, Appendix Table 3A). Additionally, the salivary microbiome in the CFAT group differed significantly from the children in the EC (*P*=0.03, F=2.1, PERMANOVA; *P*=0.01, global test, Bonferroni-corrected) and AC (*P*=0.0006, F=3.2, PERMANOVA; *P*=0.000004, global test, Bonferroni-corrected) groups (Appendix Fig. 3A; Appendix Table 3C, D). The dental plaque microbiome of the children in the C6.5 group differed significantly from those in CFAT (*P=*0.003, F=2.9, PERMANOVA; *P*=0.0007, global test, Bonferroni- corrected) (Fig. 4A; Fig. 5B; Appendix Table 3B) and from the children in the EC group (*P=*0.008, F=2.8, PERMANOVA; *P*=0.003, global test, Bonferroni-corrected) (Appendix Table 3F). Additionally, the plaque in the C6.5 group was compositionally-distinct from the plaque in the AC group (*P=*0.04, F=2.2, Bonferroni-corrected PERMANOVA; *P*=0.009, global test; *P*=0.055 after Bonferroni correction). The plaque from the CFAT group differed from the EC (*P=*0.047, F=2.0, Bonferroni-corrected PERMANOVA; *P*=0.01, global test, *P*=0.067 after Bonferroni correction) and the AC (*P=*0.002, F=2.7, PERMANOVA; *P*=0.002, global test, Bonferroni-corrected) groups (Appendix Fig. 3B; Appendix Table 3E).

At T1 and T2, neither the saliva or dental plaque microbiome differed in α-diversity indices according to the caries group (*P*>0.05, Kruskal-Wallis test), while at T3, the saliva samples in the CFAT group had significantly lower species richness than those in the C6.5 (*P*=0.04, Bonferroni-corrected Mann-Whitney test) and the AC groups (*P*=0.05, Bonferroni- corrected Mann-Whitney test) (Fig. 4B). In dental plaque at T3, the microbial diversity was significantly higher in samples in the C6.5 group than in those in the CFAT group (species richness: *P*=0.008, Shannon Diversity Index: *P*=0.004, Mann-Whitney test, Bonferroni corrected) and in the EC groups (Species richness*: P*=0.003, Shannon Diversity Index: *P*=0.002, Mann-Whitney test, Bonferroni-corrected) (Fig. 4B).

At the age of 4 years (T3), both in saliva and dental plaque, groups (zOTUs) of various species of the genus *Prevotella* and *Leptotrichia* contributed to the differences observed between the C6.5 and the CFAT groups, with a higher proportion of these taxa found in the C6.5 group (Fig. 5A, B; Appendix Table 3A, B). A group of zOTUs assigned to the genera *Selenomonas*, *Streptococcus*, *Veillonella*, *Fusobacterium*, *Capnocytophaga*, *Propionibacterium,* and *Neisseria* were found in a higher proportion in the dental plaque of the C6.5 group than in that of the EC group (Appendix Table 3F).

*Differences in individual taxa*

The top 20 most abundant genera in the salivary and dental plaque samples were compared according to caries status for each time point (Fig. 4C). Ordered on the basis of the children’s caries status (from CFAT to C6.5, C4 and C2.5), the relative abundance of genus *Actinomyces* and *Prevotella* in saliva increased significantly at T1, and that of *Porphyromonas* decreased (*P*<0.05, FDR-corrected Kruskal-Wallis test, Bonferroni-corrected Mann-Whitney test) (Appendix Fig. 4A), while no differences were observed at T2 and T3.

In dental plaque samples at T1, no genera in this top 20 showed significant differences according to caries status. At T2, the relative abundance of genus *Lautropia* and *Bergeyella* decreased with caries progression, and at T3 of genus *Prevotella*, *Fusobacterium*, *Bergeyella,* and *Aggregatibacter* were significantly different according to the caries groups (Appendix Fig. 4B). The genus *Lautropia* had a higher relative abundance in the CFAT group than in the C4 group (*P*=0.003, Bonferroni-corrected Mann-Whitney test) and the EC group (*P*=0.01, Bonferroni-corrected Mann-Whitney test). At T3, dental plaque samples in the C6.5 group had a higher relative abundance of genus *Fusobacterium* than in the EC group (*P*=0.001, Bonferroni-corrected Mann-Whitney test).

To assess which individual zOTUs contributed to the differences among the caries groups, we used the TMM-normalized dataset and performed Empirical-Bayes regression test using the ShrinkBayes algorithm (Fig. 1). To take into account the effects of the main covariates influencing the microbiome, the combination of those variables with the largest explained variation (best model) to the microbiome was included in the Empirical-Bayes regression. We described the analyses on the individual zOTU-level only for those caries groups which showed significant differences in one or both (PERMANOVA and the global test) community-level analyses.

In saliva collected at T1, the following covariates were selected as the best model by the bioenv function and then taken into account while assessing the differences in individual taxa: child age, exposure to antibiotics, race/ethnicity, Medicaid status, number of teeth present, the covariate defined in Question 18 (frequency of consumption of sugary drinks between meals by the children) and frequency of dental visits of the children (Question 23) and their caregivers (Question 42). In total, 6 zOTUs discriminated salivary sample significantly among C4 and CFAT groups. This made it possible to differentiate between the C4 group and the CFAT group (6 zOTUs: three zOTUs belonged to genus *Streptococcus*, two to *Haemophilus*, and one to *Leptotrichia*) (*BFDR*≤0.1, ShrinkBayes) (Appendix Table 4A). At T1 the microbial profiles of dental plaque did not differ significantly according to the caries groups; therefore, analyses on individual zOTUs were not performed.

At T2, the microbial profiles of saliva did not differ significantly according to caries group. In the dental plaque collected at 2.5 years of age (T2), four covariates were selected as the best model: exposure to antibiotics, Medicaid status, tight or tethered maxillary labial frenulum, and Question 18 (frequency of consumption of sugary drinks between meals by the children). Several zOTUs belonging to genus *Corynebacterium (C. matruchotii)*, *Leptotrichia (L. hofstadii)*, *Rothia (R. dentocariosa)*, *Streptococcus (S. gordonii/HOT 056* and *S. cristatus/oligofermentans/sinensis)*, and *Veillonella* (*V. dispar*) significantly discriminated between the dental plaque microbiome of those in the C4 group and those in the CFAT group, and between those in the CFAT and those in EC groups (*BFDR*≤0.1, ShrinkBayes) (Appendix Table 4B, C).

In the salivary samples collected at the age of 4 years (T3), exposure to antibiotics and bacterial DNA concentration were used as covariates for the ShrinkBayes analyses. The results revealed that 81 zOTUs significantly discriminated the C6.5 group from the children in CFAT group (*BFDR*≤0.1, ShrinkBayes) (Appendix Table 5A). Several zOTUs belonging to genus *Alloprevotella (A. HOT 308), Campylobacter (C. concisus),* unclassified *Kingella/Neisseria, Lachnoanaerobaculum (L. orale), Leptotrichia (L. HOT 417), Prevotella (P. histicola, P. nigrescens, P. oulorum, P. pallens, P. scopos/HOT 313/HOT 314,* and *P. veroralis), Rothia (R. mucilaginosa), Stomatobaculum (S. longum), Streptococcus (S. gordonii/HOT 056)* and *Tannerella (T. HOT 286/HOT 808)* significantly discriminated the C6.5 group from the CFAT group*.* Some of those zOTUs were plotted in the Appendix Fig. 5A. Ten zOTUs significantly discriminated the salivary microbiome in the CFAT (*Alloprevotella HOT 914*) group from that in the EC (nine zOTUs) group (*BFDR*≤0.1, ShrinkBayes) (Appendix Table 5B). When saliva in the CFAT group was compared with that in the AC group, 10 zOTUs were at a higher proportion in CFAT, and 96 zOTUs were at a higher proportion in the AC group (Appendix Fig. 5B; Appendix Table 5C).

For the ShrinkBayes analyses of the dental plaque samples at T3, three covariates were selected as the best model: exposure to antibiotics, tight or tethered maxillary labial frenulum, and bacterial DNA concentration in the sample. At the level of the individual taxa, 101 zOTUs significantly discriminated between the dental plaque in C6.5 group and that of the CFAT group, 95 zOTUs of which were at a higher proportion in the C6.5 group (*BFDR*≤0.1, ShrinkBayes) (Appendix Table 6A). Several zOTUs belonging to genus *Actinomyces (A. HOT 414, A. HOT 448), Campylobacter (C. gracilis, C. HOT 324), Fusobacterium (F. nucleatum subspecies animalis), Leptotrichia (L. HOT 212, L. HOT 417, L. wadei), Prevotella (P. histicola, P. maculosa, P. nigrescens, P. oris,* and *P. salivae),* and *Selenomonas (S. HOT 442/sputigena)* significantly discriminated the C6.5 group from the CFAT group (Appendix Fig. 5C). Moreover, 43 zOTUs were more abundant in the dental plaque microbiome of the children in the C6.5 group compared to that of the EC group (*BFDR*≤0.1, ShrinkBayes) (Appendix Table 6A), while 47 zOTUs were higher in plaque of the C6.5 group versus 12 zOTUs of the AC group (*BFDR*≤0.1, ShrinkBayes) (Appendix Table 6B). Specifically, the dental plaque in the C6.5 group was discriminated by, among others, zOTUs belonging to genus *Campylobacter (C. rectus/showae), Capnocytophaga (C. HOT 332, C. leadbetteri), Corynebacterium (C. matruchotii), Kingella (K. HOT 012),* unclassified *Kingella/Neisseria, Lachnoanaerobaculum (L. umeaense), Leptotrichia (L. hofstadii, L. HOT 212, L. HOT 225),* and *Selenomonas (S. noxia)* compared to those in the AC group (Appendix Fig. 5C). When the plaque samples in the CFAT group were compared to those in the EC group, 24 zOTUs were significantly higher in the CFAT and 9 zOTUs were higher in the EC group (*BFDR*≤0.1, ShrinkBayes) (Appendix Table 6B). The difference between the CFAT and the AC groups was determined by 99 zOTUs (*BFDR*≤0.1, ShrinkBayes) (Appendix Table 6C). For example, zOTUs belonging to genus *Abiotrophia (A. defective), Aggregatibacter (A. aphrophilus/paraphrophilus, A. HOT 513/HOT 898), Alloprevotella (A. HOT 473), Cardiobacterium (C. hominis), Corynebacterium (C. durum), Fusobacterium (F. periodonticum), Haemophilus (H. parahaemolyticus/ paraphrohaemolyticus/sputorum), Kingella (K. HOT 012),* unclassified *Kingella/Neisseria, Leptotrichia (L. HOT 212),* and *Rothia (R. aeria)* were significantly more abundant in dental plaque in the CFAT group (Appendix Fig. 5D).

ZOTUS CLASSIFIED AS *STREPTOCOCCUS MUTANS* AND *PORPHYROMONAS GINGIVALIS*

We assessed the presence and relative abundances of zOTUs classified as *Streptococcus mutans* and *Porphyromonas gingivalis* in the salivary and dental plaque samples of the children at three time points (T1, T2, and T3) and salivary samples of their caregivers collected at T1. In the entire dataset (1,514 samples of the children and 251 samples of the caregivers), 7 zOTUs were classified as *S. mutans*, while only a single zOTU (zOTU 209) was classified as *P. gingivalis*. The prevalence of *P. gingivalis* was 1 read or 0.01% in eight different children: in saliva there was one at T2 and four at T3, and in dental plaque samples, there were three: one at each of T1, T2, and T3. The *P. gingivalis* zOTU was present in 44 salivary samples of the caregivers at a relative abundance of 0.01-2.4%. None of these caregivers were caregivers of any of the eight children with *P. gingivalis*.

From the 266 children and their caregivers, *S. mutans* was present in salivary samples of 67 children (25.2%) and 115 caregivers (43.2%), and in dental plaque samples of 62 children (23.3%). Seventeen child-caregiver pairs presented with *S. mutans* zOTUs in all three sample types (salivary samples from the children and their caregivers and dental plaque samples of the children). In salivary samples only, *S. mutans* zOTUs were found in 13 child-caregiver pairs. Eight child-caregiver pairs showed reads of *S. mutans* zOTUs in the saliva of the caregivers and only dental plaque of the children. Additionally, *S. mutans* zOTUs were identified in salivary samples of 77 caregivers (65.2%) whose child samples did not have the *S. mutans* zOTUs. In 44 children, *S. mutans* was present while absent in salivary samples of their primary caregivers. Among these, 30 children had *S. mutans* zOTUs in both sample types (salivary and dental plaque).

To assess the differences in *S. mutans* by caries group, the relative abundances of the 7 zOTUs classified as *S. mutans* were summed and analyzed at each time point. Although at T1 there was no difference in the proportion of *S. mutans* by caries status, at the later time points (T2, T3) we observed a significant difference in *S. mutans* by the caries groups (*P*<0.05, Kruskal-Wallis test) (Appendix Fig. 6). Saliva collected at T2 and T3 from the advanced caries (AC) group had significantly higher relative abundance of *S. mutans* compared to that of the children who were caries-free at all time points (CFAT) and children with caries at T4 (C6.5) (*P*<0.05, Bonferroni-corrected Mann-Whitney test) (Appendix Fig. 6A). In dental plaque collected at T2, the children with caries at T3 (C4 group) had a significantly higher relative abundance of *S. mutans* compared to that of the CFAT group (*P*=0.0001, Bonferroni-corrected Mann-Whitney test). At the same time point, the dental plaque of the early caries (EC) and advanced caries (AC) groups had higher relative abundance of *S. mutans* compared to that of the children who were caries-free at all time points (CFAT). Additionally, the proportion of *S. mutans* was higher in the advanced caries (AC) group versus children with caries at T4 (C6.5) (*P*<0.05, Bonferroni-corrected Mann-Whitney test) (Appendix Fig. 6B). At T3, the C6.5 and the AC groups had significantly higher relative abundance of *S. mutans* compared to the CFAT group (*P*<0.05, Bonferroni-corrected Mann-Whitney test) (Appendix Fig. 6B).

Next, with those children who were included in the caries groups comparisons, we assessed the proportions of *S. mutans* in saliva and dental plaque samples by dietary habits of the children (Questions 14, 15, 16, 17, and 18) at each time point. The group of 1-year-old children (T1) with “daily nursing or bottle use” (Question 14) had significantly higher relative abundance of *S. mutans* in their dental plaque samples compared to the groups performing this habit less frequently (*P*=0.01, Bonferroni-corrected Kruskal-Wallis test). In saliva samples collected at T2 and dental plaque samples collected at T2 and T3, the children who consumed “sugary drinks between meals daily” (Question 18) had significantly higher relative abundance of *S. mutans* compared to the children who consumed sugary drinks less frequently, irrespective of their caries status (*P*<0.05, Bonferroni-corrected Kruskal-Wallis test).

DEMOGRAPHICS, DIETARY FACTORS, AND ORAL HYGIENE HABITS

This part of the results describes the demographics and dietary habits of the children and their caregivers according to the caries status of the children over time. No significant differences were found in age (months), sex, delivery mode or exposure to antibiotics among the children with different caries statuses over time (*P*>0.05, Multinomial logistic regression). There was, however, a significant difference observed across different caries groups by Medicaid status and recruitment site at all three time points, and by race/ethnicity and income variables at T2 and T3 only (*P*<0.05, Multinomial logistic regression).

Next, we tested whether the dietary habits of the children and their caregivers differed according to the caries groups at each time point (T1, T2 and T3). From eight variables related to eating habits (Appendix Table 1), only frequency of consumption between meals of sugary drinks such as regular soda, sweet tea, chocolate milk, strawberry milk, sports drinks or fruit juice by the children themselves (Question 18) (Appendix Fig. 7A) and by their caregivers (Question 39) (Appendix Fig. 7B) showed significant associations with caries status of the children at all three time points (*P*<0.05, Multinomial logistic regression). Interestingly, Question 39 which is frequency of consumption of sugary drinks between meals by the caregivers of the children also showed a significant association with caries status of their children at all three time points (*P*<0.05, Multinomial logistic regression) (Appendix Fig. 7B). As expected, significantly more caregivers of the children with advanced caries lesions (AC) indicated that they gave sugary drinks between meals daily to their children than the caregivers of the other caries groups (*P*<0.05) (Appendix Fig. 7A). The caregivers themselves whose children had advanced caries lesions (AC) consumed sugary drinks significantly more often than the caregivers whose children were in the caries-free at all time points (CFAT) (*P*<0.05) (Appendix Fig. 7B) group. Furthermore, the caregivers of the children who were diagnosed with caries at T4 (C6.5) consumed sugary drinks significantly more frequently than did the caregivers of the children who were caries-free at all time points (CFAT) (*P*<0.05) (Appendix Fig. 7B). Additionally, at T2, Question 38 (frequency of the consumption of sugary snacks between meals by the caregivers) and Question 40 (consumption of anything other than plain water before going to bed by the caregivers) also were significantly associated with caries status of their children (*P*<0.05; Multinomial logistic regression).

The associations of six variables related to the oral care habits of the children (Appendix Table 1) with caries status of the children over time were investigated. A single variable indicating frequency at which the caregivers inspected the dentition of their children (Question 10) showed a significant association with caries status of the children (*P*<0.05, Multinomial logistic regression test) at T2 and T3.

**REFERENCES**:

Banting DW, Eggertsson H, Zandoná AGF, Ismail AI, Longbottom C, Pitts NB, Reich E, Ricketts D, Selwitz RH, Sohn W. 2012. Rationale and evidence for the international caries detection and assessment system (icdas ii) author: International caries detection and assessment system coordinating committee.

Benjamini Y, Hochberg Y. 1995. Controlling the false discovery rate: A practical and powerful approach to multiple testing. Journal of the Royal Statistical Society: Series B (Methodological). 57(1):289-300.

Chen T, Yu WH, Izard J, Baranova OV, Lakshmanan A, Dewhirst FE. 2010. The human oral microbiome database: A web accessible resource for investigating oral microbe taxonomic and genomic information. Database. 2010:baq013.

Ciric L, Pratten J, Wilson M, Spratt D. 2010. Development of a novel multi-triplex qpcr method for the assessment of bacterial community structure in oral populations. Environ Microbiol Rep. 2(6):770-774.

Daly JA-O, Xu Y, Yanca E, Levy SM, Levy BT, Talbert J, Tran JL, Ann Keels M, Fontana M. 2022. Primary caregiver retention and perceptions of retention strategies in a 36-month prospective childhood caries study. J Prim Care Community Health. 13(Electronic).

Daly JM, Levy SM, Xu Y, Jackson RD, Eckert GJ, Levy BT, Fontana M. 2016. Factors associated with parents' perceptions of their infants' oral health care. J Prim Care Community Health. 7(3):180-187.

Eckert GJ, Jackson R, Fontana M. 2010. Sociodemographic variation of caries risk factors in toddlers and caregivers. Int J Dent. 2010.

Falony G, Joossens M, Vieira-Silva S, Wang J, Darzi Y, Faust K, Kurilshikov A, Bonder MJ, Valles-Colomer M, Vandeputte D et al. 2016. Population-level analysis of gut microbiome variation. Science. 352(6285):560-564.

Fontana M, Eckert GJ, Keels MA, Jackson R, Katz BP, Kemper AR, Levy BT, Levy SM, Yanca E, Kelly S et al. 2019. Predicting caries in medical settings: Risk factors in diverse infant groups. J Dent Res. 98(1):68-76.

Fontana M, Jackson R, Eckert G, Swigonski N, Chin J, Zandona AF, Ando M, Stookey GK, Downs S, Zero DT. 2011. Identification of caries risk factors in toddlers. J Dent Res. 90(2):209-214.

Goeman JJ, van de Geer SA, de Kort F, van Houwelingen HC. 2004. A global test for groups of genes: Testing association with a clinical outcome. Bioinformatics. 20(1):93-99.

Hammer O, Harper DAT, Ryan P. 2001. Past: Paleontological statistics software package for education and data analysis. Palaeontol Electron. 4:1-9.

Huber W, von Heydebreck A, Sültmann H, Poustka A, Vingron M. 2002. Variance stabilization applied to microarray data calibration and to the quantification of differential expression. Bioinformatics. 18 Suppl 1:S96-104.

Kahharova D, Brandt BW, Buijs MJ, Peters M, Jackson R, Eckert G, Katz B, Keels MA, Levy SM, Fontana M et al. 2020. Maturation of the oral microbiome in caries-free toddlers: A longitudinal study. J Dent Res. 99(2):159-167.

Kozich JJ, Westcott SL, Baxter NT, Highlander SK, Schloss PD. 2013. Development of a dual-index sequencing strategy and curation pipeline for analyzing amplicon sequence data on the miseq illumina sequencing platform. Appl Environ Microbiol. 79(17):5112-5120.

Oksanen J, Blanchet FG, Friendly M, Kindt R, Legendre P, McGlinn D, Minchin P, O’Hara RB, Simpson G, Solymos P et al. 2020. Vegan community ecology package version 2.5-7 november 2020.

Robinson MD, McCarthy DJ, Smyth GK. 2010. Edger: A bioconductor package for differential expression analysis of digital gene expression data. Bioinformatics. 26(1):139-140.

Robinson MD, Oshlack A. 2010. A scaling normalization method for differential expression analysis of rna-seq data. Genome Biol. 11(3):R25.

Storey JD. 2003. The positive false discovery rate: A bayesian interpretation and the q -value. Ann Statist. 31(6):2013-2035.

van de Wiel MA, Neerincx M, Buffart TE, Sie D, Verheul HM. 2014. Shrinkbayes: A versatile r-package for analysis of count-based sequencing data in complex study designs. BMC Bioinformatics. 15:116.

Vollmer T, Stormer M, Kleesiek K, Dreier J. 2008. Evaluation of novel broad-range real-time pcr assay for rapid detection of human pathogenic fungi in various clinical specimens. J Clin Microbiol. 46(6):1919-1926.

Wang Q, Garrity GM, Tiedje JM, Cole JR. 2007. Naive bayesian classifier for rapid assignment of rrna sequences into the new bacterial taxonomy. Appl Environ Microbiol. 73(16):5261-5267.

**APPENDIX FIGURES:**


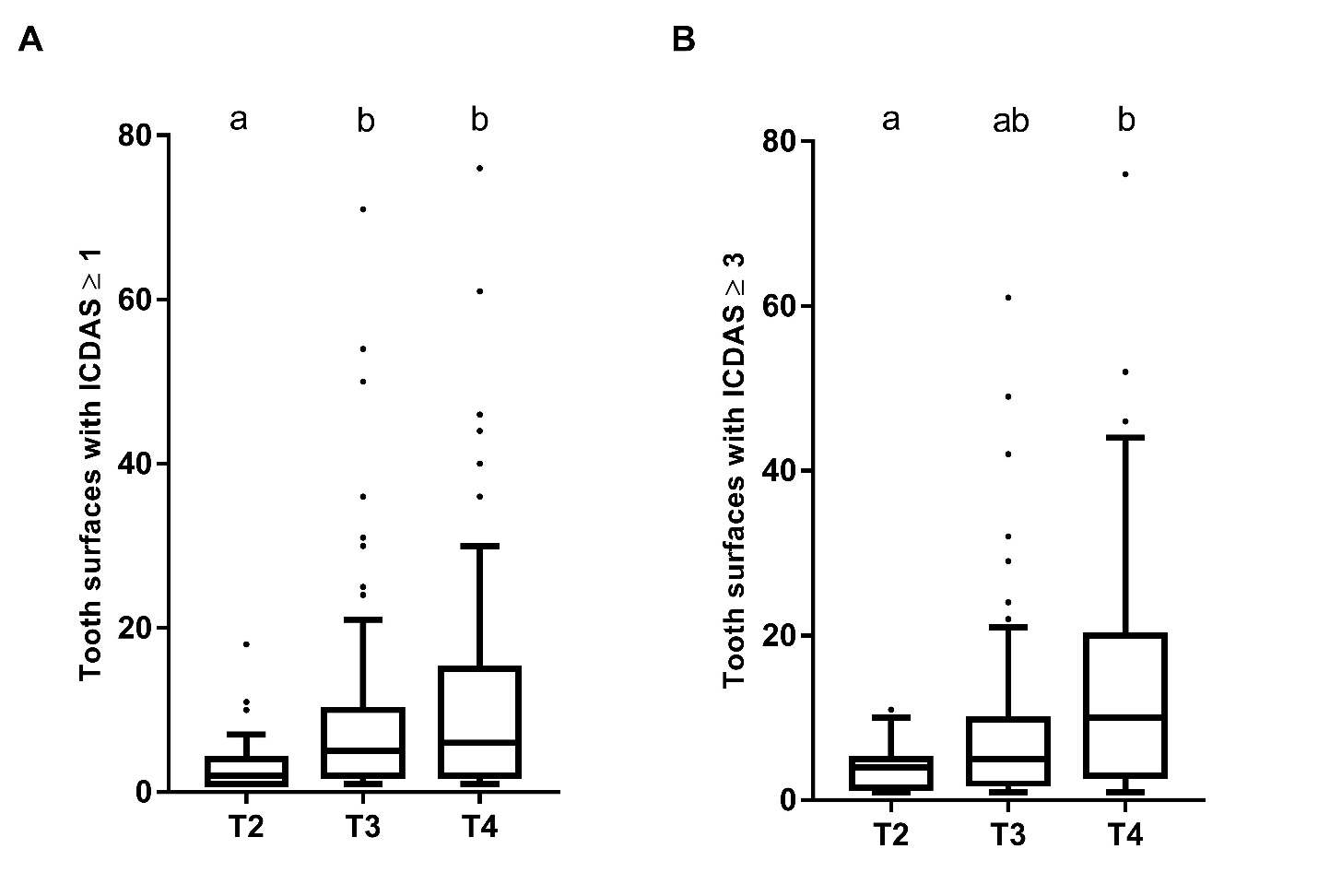


**Appendix Figure 1:** Number of early (ICDAS score 1 and 2) and advanced (ICDAS≥3) caries lesions **(A)** and advanced caries lesions **(B)** of the children at T2-T4. The boxes are plotted using Tukey’s method. The X-axis shows the time points of the clinical dental examinations and the Y-axis represents the number of tooth surfaces with early and advanced caries lesions. Different letters indicate a statistically significant difference (Kruskal-Wallis test followed by Bonferroni-corrected Mann-Whitney test) between time points.


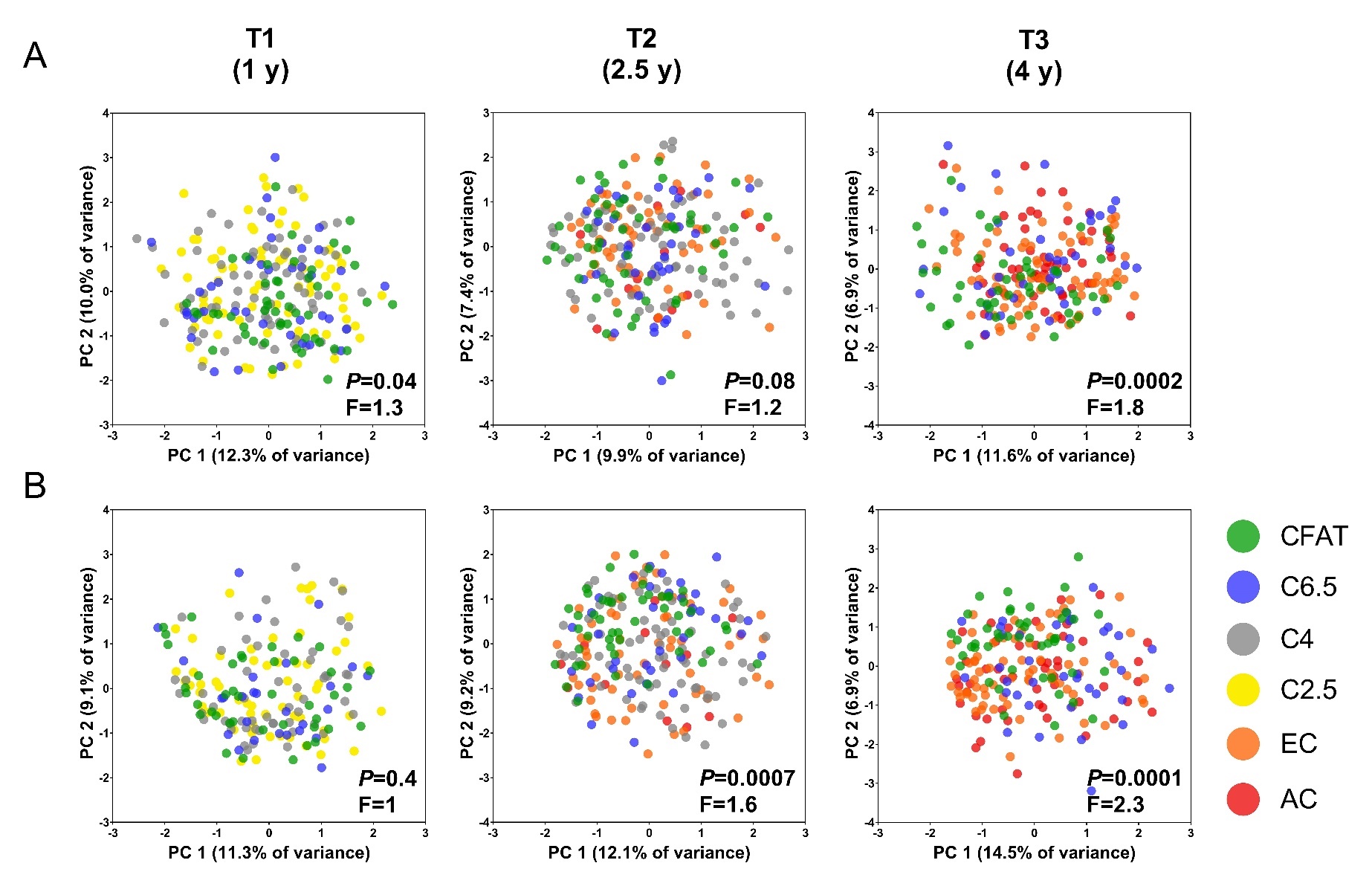


**Appendix Figure 2:** The microbial profiles of the salivary and dental plaque samples of the children according to their caries groups over time. Principal Component Analysis (PCA) plots depict the microbiome compositions of the salivary **(A)** and dental plaque **(B)** samples of the children at T1 (~1 years of child age), T2 (~2.5 years of age) and T3 (~4 years of age). Green dots - samples collected from the children who were caries-free at all time points (CFAT) (*n*=50), blue - samples collected from the children with caries at 6.5 y (C6.5) (*n*=38), grey - samples collected from the children with caries at 4 y (C4) (*n*=70), yellow - samples collected from the children with caries at 2.5 y (C2.5) (*n*=75), orange - samples collected from the children with early caries (EC) (ICDAS score 1 and 2) (at T2, *n*=63: at T3, *n*=75) and red - advanced caries (AC) (ICDAS≥3) (at T2, *n*=13: at T3, *n*=52) at the current clinical examination. Axes show the first two principal components (PCs) explaining the highest inter-sample variation (% of variance). The *P*- and F-values indicate the output of PERMANOVA analyses, using Bray-Curtis similarity.


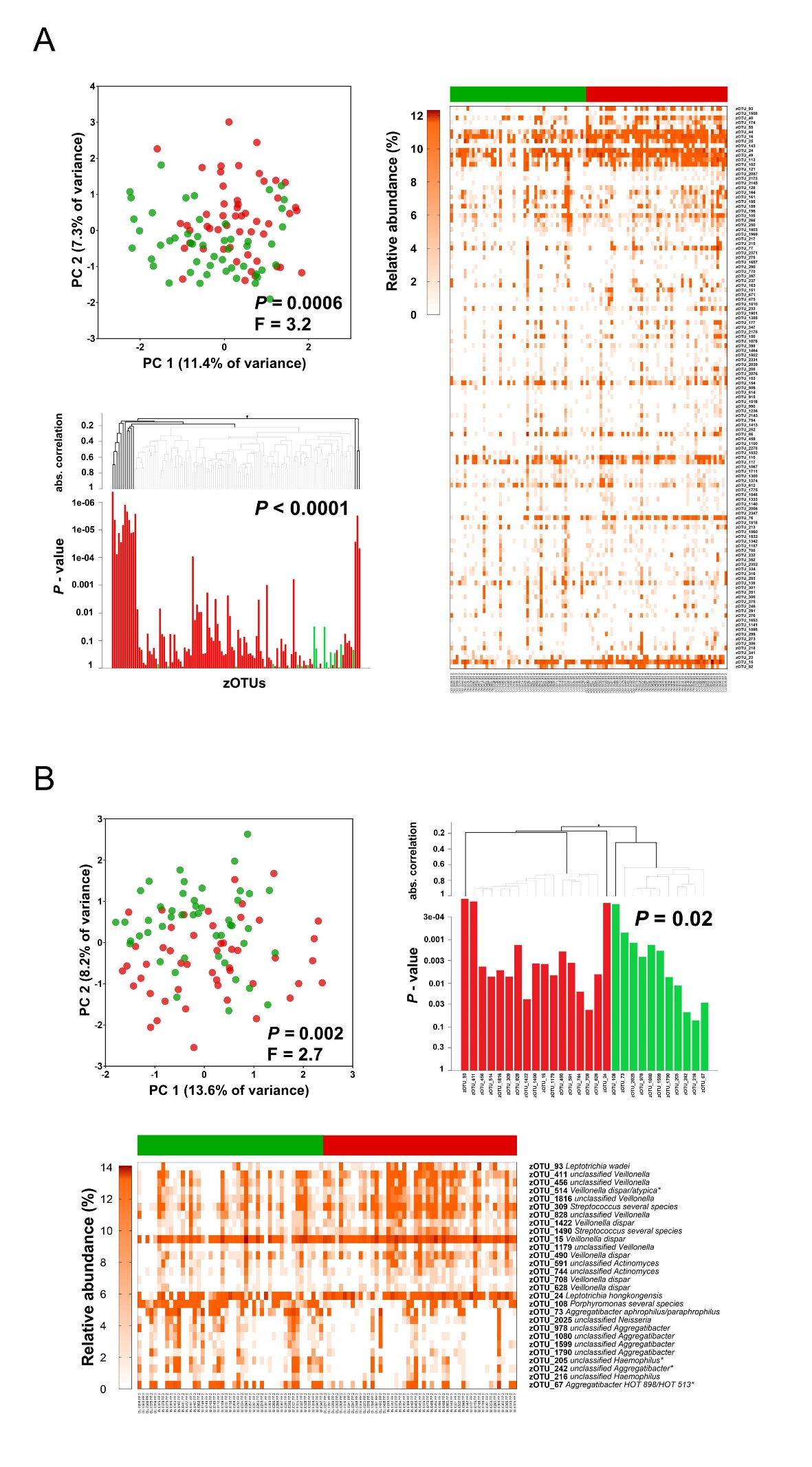


**Appendix Figure 3:** The **(A)** salivary and **(B)** dental plaque microbiome profiles of the children at T3 in the caries-free at all time points (CFAT) (green) group (*n*=50) versus the advanced caries (AC) (red) group (*n*=52). Principal Component Analysis (PCA) plots, dendrograms and heatmaps of the salivary and dental plaque microbiome composition are shown. The *P*- and F-values indicate the output of PERMANOVA analyses, using Bray-Curtis similarity. The dendrograms depict the zOTUs contributing to the global test analyses. Bars represent the zOTUs selected with the global test and they are colored according to the caries groups (green - zOTUs associated with CFAT group and red - AC group). Individual zOTUs and groups of zOTUs contributing to the significant difference of the global test are shown by the black lines on top of the bars. *P*-values represent the overall test results between the compared groups. The X-axis shows single zOTUs and zOTU groups associated with the caries groups. The Y-axis shows the *P*-value corresponding to the test for the association of each individual zOTU with the caries groups. The *P*-value is displayed on a logarithmic scale, so small *P*-values correspond to higher bars. The heatmaps depict the relative abundances of significant zOTUs and zOTU groups tested with the global test among CFAT vs. AC group. The zOTUs are ordered according to the dendrogram. Appendix Table 3D, E show group-wise and individual *P*-values of these zOTUs, in the same order. * indicates zOTUs additionally blasted on the HOMD website.


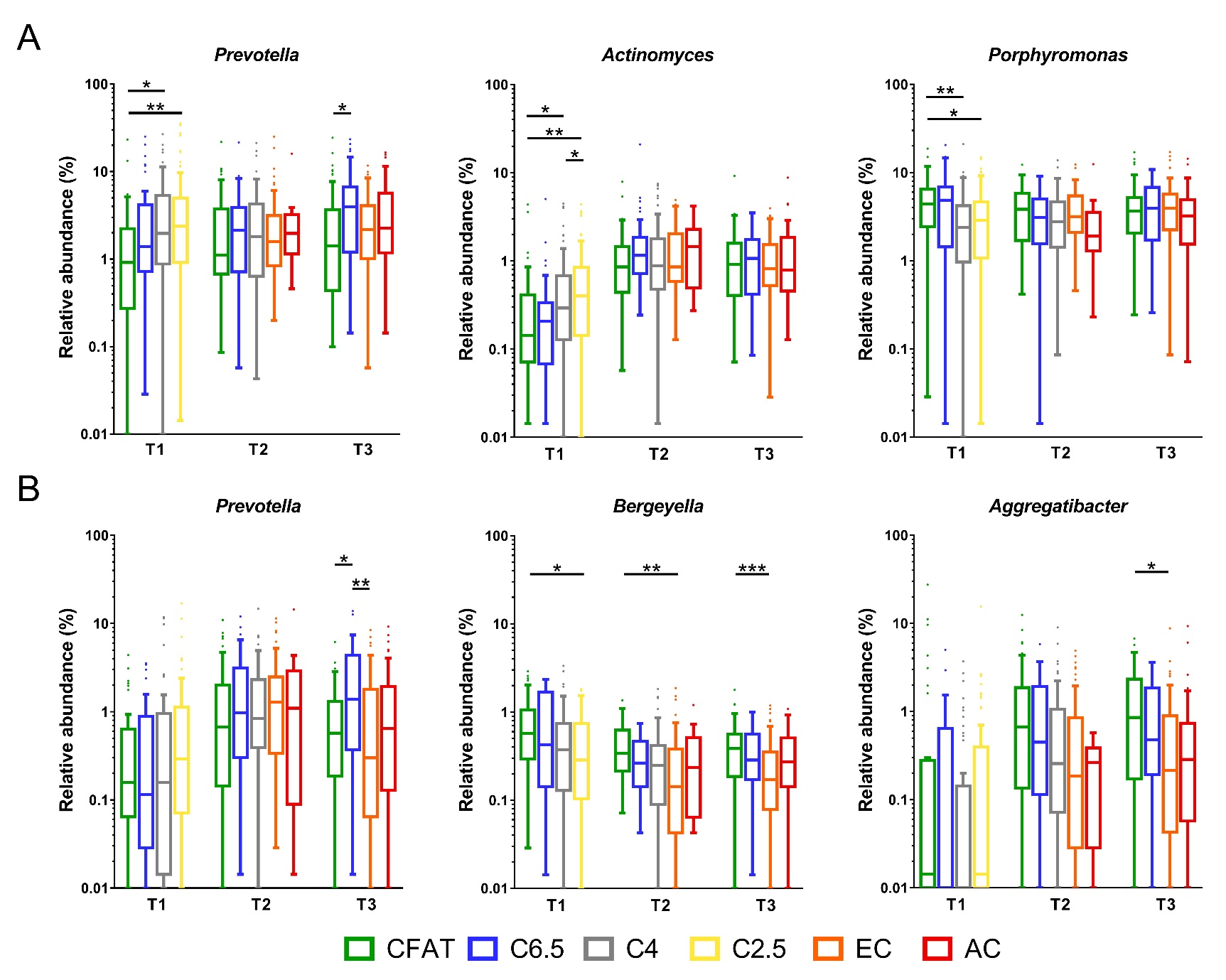


**Appendix Figure 4:** The relative abundance of bacterial genera in salivary **(A)** and dental plaque **(B)** samples of the children by their caries status over time. The boxes are plotted using Tukey’s method. Significant differences over time within the respective sample type are indicated by asterisks: **P*<0.05, ***P*<0.01, and ****P*<0.001 (FDR-corrected Kruskal-Wallis test and Bonferroni-corrected Mann-Whitney test). Different colors of the boxes indicate the caries groups. Green boxes - samples collected from the children who were caries-free at all time points (CFAT) (*n*=50), blue - samples collected from the children with caries at 6.5 y (C6.5) (*n*=38), grey - samples collected from the children with caries at 4 y (C4) (*n*=70), yellow - samples collected from the children with caries at 2.5 y (C2.5) (*n*=75), orange - samples collected from the children with early caries (EC) (ICDAS score 1 and 2) (at T2, *n*=63: at T3, *n*=75) and red - advanced caries (AC) (ICDAS≥3) (at T2, *n*=13: at T3, *n*=52) at the current clinical examination. Lines connect the caries groups with the respective difference.


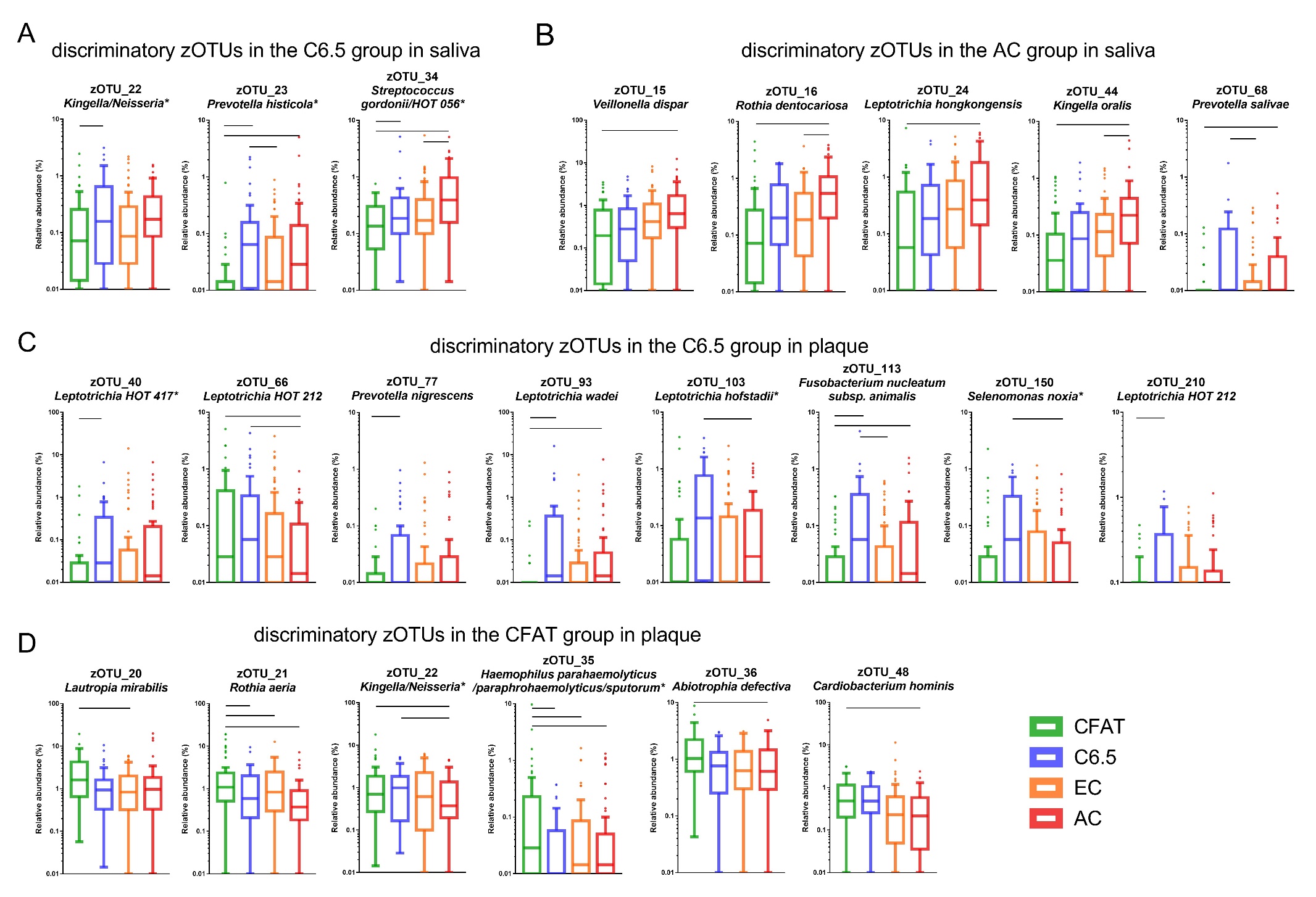


**Appendix Figure 5:** The relative abundances of zOTUs in **(A, B)** salivary and **(C, D)** dental plaque samples selected with pairwise ShrinkBayes analyses according to the caries status of the children at T3: Green - caries-free at all time points (CFAT), blue - caries at 6.5 (C6.5), orange - early caries (EC), and red - advanced caries (AC). The boxplots show discriminatory zOTUs in the C6.5 group compared to other caries groups in **(A)** saliva and **(C)** dental plaque samples, discriminatory zOTUs in the AC group compared to other caries groups in **(B)** saliva and discriminatory zOTUs in the CFAT group compared to other caries groups in **(D)** dental plaque samples. The boxes are plotted using Tukey’s method. Lines connect the study groups with the significant differences (BFDR≤0.1, ShrinBayes). Different colors of the boxes indicate the caries groups. The analysis was done on TMM-normalized data (ShrinkBayes test) and plotted on the subsampled dataset. * indicates zOTUs additionally blasted on the HOMD website.


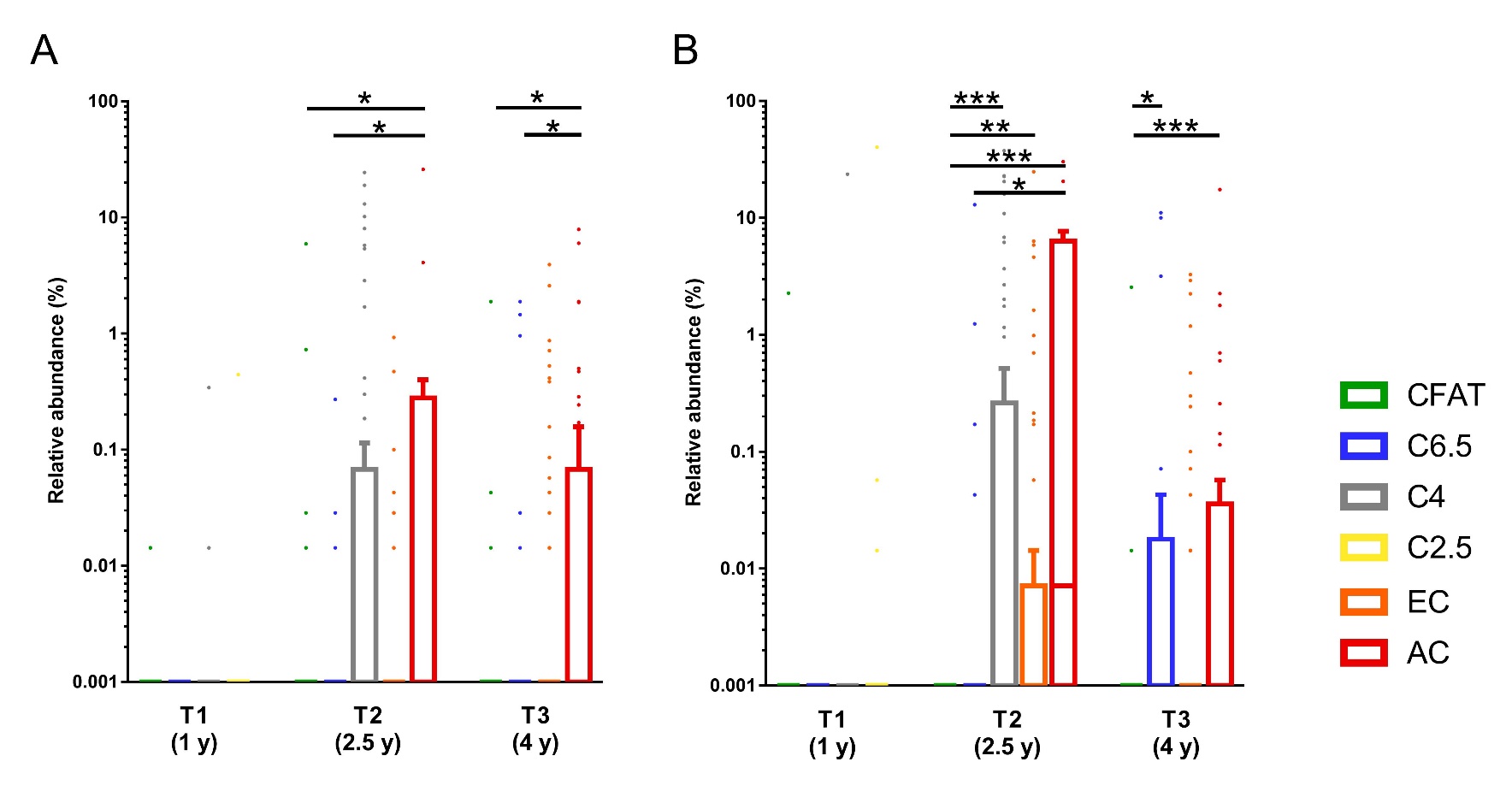


**Appendix Figure 6:** The relative abundances of summed zOTUs classified as *Streptococcus mutans* in **(A)** salivary and **(B)** dental plaque samples of the children by their caries groups over time. The boxes are plotted using Tukey’s method. Significant differences over time within the respective sample type are indicated by asterisks: **P*<0.05, ***P*<0.01, and ****P*<0.001 (Kruskal-Wallis test and Bonferroni-corrected Mann-Whitney test). Different colors of the boxes indicate the caries groups. Green boxes - samples collected from the children who were caries-free at all time points (CFAT) (*n*=50), blue - samples collected from the children with caries at 6.5 y (C6.5) (*n*=38), grey - samples collected from the children with caries at 4 y (C4) (*n*=70), yellow - samples collected from the children with caries at 2.5 y (C2.5) (*n*=75), orange - samples collected from the children with early caries (EC) (ICDAS score 1 and 2) (at T2, *n*=63: at T3, *n*=75) and red - advanced caries (AC) (ICDAS≥3) (at T2, *n*=13: at T3, *n*=52) at the current clinical examination. Lines connect the caries groups with the respective difference.


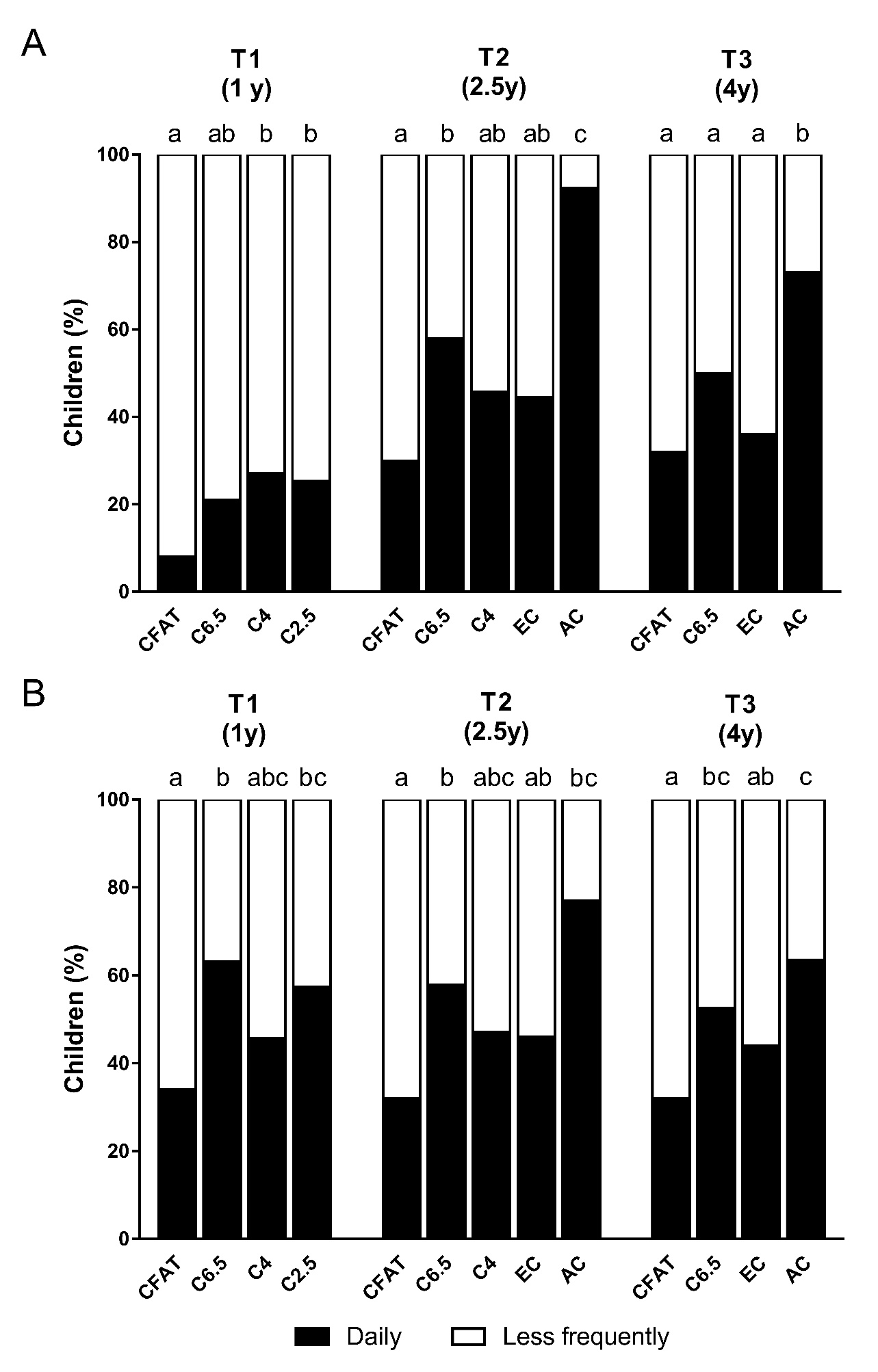


**Appendix Figure 7:** Frequency of consumption of sugary drinks **(A)** by children (Question 18 of the questionnaire) and **(B)** by their caregivers (Question 39) according to the caries status of the children over time. Black stacked bars indicate daily consumption of sugary drinks such as regular soda, sweet tea, chocolate milk, strawberry milk, or fruit juice between meals; white bars are for less frequent consumption (weekly, monthly and/or never) of sugary drinks. Analyses were performed separately at each time point (T1, T2 and T3). Different letters indicate statistically significant differences (*P*<0.05, Multinomial logistic regression test) among caries groups at the respective time point.

**APPENDIX TABLES:**

**Appendix Table 1:** The caries risk factors (variables) from the Primary Caregiver Questionnaire, sample collection and processing, and the oral examination used for analysis in the current study.

| **N** | **Variables** | **Description** | **Type** |
| --- | --- | --- | --- |
| **Demographics and general questions** | | |  |
| 1 | Sex | Sex of the children | Categorical |
| 2 | Race/ethnicity | Question 30: What is your child’s racial background? | Categorical |
| 3 | Delivery mode | Question 32: Was your child delivered by C-section? | Categorical |
| 4 | Child age | Child age (months) at each study visit (time points) | Continuous |
| 5 | Antibiotics | Antibiotic use in last 4 weeks prior each study visit | Categorical |
| **Sample collection, processing and sequencing** | | |  |
| 6 | 16S concentration | Bacterial DNA concentration (pg/ul) in the sample | Continuous |
| 7 | Run | The batch number of run the sample sequencing was performed | Categorical |
| **Oral examination** | | |  |
| 8 | Presence of teeth | The number of the teeth present at baseline (T1): predentate, 1-4 teeth and 5-more teeth | Categorical |
| 9 | Number of teeth | The number of the teeth present at second visit (T2): incomplete (≤20) and complete (20) dentition | Categorical |
| 10 | Closed contact | If the lower frontal teeth (between LL3 (Cuspid) and LR3 (Cuspid)) have spaces between them or in contact with each other (no space) at each study visit | Categorical |
| 11 | Space between incisors | If the upper incisors (between UR1 and UL1) have spaces between them or in contact with each other (no space) at each study visit |  |
| 12 | Fluoride varnish | If the child received fluoride varnish application between the study visits | Categorical |
| 13 | Frenum abnormality | If the child has thick or tethered maxillary labial frenum at each study visit | Categorical |
| **Eating habits of the children** | | |  |
| 14 | Bottle/sippy cup a day | Question 13: Does your child usually (throughout the day) drink from a bottle or sippy cup? | Categorical |
| 15 | Nursing/bottle overnight | Question 14: How often does your child go to sleep while nursing or while drinking something other than water from a bottle/sippy cup? | Categorical |
| 16 | Eating before bed (child) | Question 15: How often does your child eat or drink anything other than plain water before going to bed (and after you have brushed his/her teeth if teeth are brushed)? | Categorical |
| 17 | Drinking tap water (child) | Question 16: How often does your child typically drink tap water - including filtered water from the refrigerator? | Categorical |
| 18 | Sugary snacks (child) | Question 17: How often do you give your child sugary snacks such as raisins, candy, cookies, cakes, or cereal between meals? | Categorical |
| 19 | Sugary drinks (child) | Question 18: How often do you give your child sugary drinks such as regular soda, sweet tea, chocolate milk, strawberry milk or fruit juice between meals? | Categorical |
| **Eating habits of the caregivers** | | |  |
| 20 | Sugary snacks (caregiver) | Question 38: How often do you eat sugary snacks such as raisins, candy, cookies, cakes, or cereal bars between meals? | Categorical |
| 21 | Sugary drinks (caregiver) | Question 39: How often do you drink sugary drinks such as regular soda, sweet tea, chocolate milk, strawberry milk, sports drinks or fruit juice between meals? | Categorical |
| 22 | Eating before bed (caregiver) | Question 40: How often do you eat or drink anything other than plain water before going to bed (and after brushing your teeth, if teeth are brushed)? | Categorical |
| **Behavior of the caregivers with their children** | | |  |
| 23 | Sugar pacifier cleaning | Question 19: How often do you clean your child's pacifier with juice, soda, honey or sweet drink? | Categorical |
| 24 | Oral pacifier cleaning | Question 20: How often do you clean your child's pacifier by putting it in your mouth? | Categorical |
| 25 | Food sharing | Question 21: How often do you share/taste food with your child using the same spoon, fork, glass or another utensil? | Categorical |
| 26 | Oral kissing | Question 22: How often do you kiss your child on the mouth? | Categorical |
| **Tooth care of the children** | | |  |
| 27 | Topical fluoride | Question 05: Does your child receive topical fluoride from a health professional (doctor, dentist, nurse, hygienist etc.)? | Categorical |
| 28 | Toothbrushing | Question 06: How often does an adult brush your child's teeth? | Categorical |
| 29 | Toothpaste | Question 07: How often are your child's teeth brushed with toothpaste? | Categorical |
| 30 | Non-F toothpaste | Question 08: How often are your child's teeth brushed with non-fluoride toothpaste? | Categorical |
| 31 | Mouth inspection | Question 10: How often do you check your child's teeth for anything unusual? | Categorical |
| 32 | Mouth cleaning | Question 12: How often do you clean inside your child's mouth and/or gums? | Categorical |
| **Teeth and tooth care of the caregivers** | | |  |
| 33 | Caries status (caregiver) | Question 34: Have you had cavities, fillings and/or teeth pulled in the last two years? | Categorical |
| 34 | Gum bleeding (caregiver) | Question 35: How often do your gums bleed when you brush? | Categorical |
| **Dental care and health insurance of the children** | | |  |
| 35 | Dental check-ups (child) | Question 23: How often do you take your child to the dentist? | Categorical |
| 36 | Medicaid status | Question 25: Is your child's care covered by Medicaid or State Insurance? | Categorical |
| **Medical/dental care of the caregivers** | | |  |
| 37 | Medical check-ups (caregiver) | Question 41: How often do you see your health care provider for regular check-ups? | Categorical |
| 38 | Dental check-ups (caregiver) | Question 42: How often do you get dental check-ups? | Categorical |
| **Perception of the caregivers towards the medical/dental care of their children** | | |  |
| 39 | Child's oral care | Question 51: I do a/ an ___ job taking care of the child's teeth and/or gums (past behavior) | Categorical |

**Appendix Table 2:** zOTUs contributing to the difference in the microbiome composition of the saliva samples of the children at T1 **(A)** and the dental plaque samples at T2 **(BC)**. Only zOTUs with individual *P*<0.05 are shown.

| **A** | **saliva samples in the CFAT vs C4 group at T1** | | | | | |
| --- | --- | --- | --- | --- | --- | --- |
|  | ***n* of zOTU groups** | **Numbers and taxonomical names of the zOTUs at genus and species level** | **Associated group** | ***P*-value of the individual zOTU** | ***P*-value of the group of zOTUs corrected for multiple testing** | **Bonferroni- corrected Overall *P*-value** |
|  | 1 | *zOTU_2254 Gemella haemolysans/morbillorum/sanguinis* | CFAT | 9.40E-05 | 1.18E-02 | 2.90E-02 |
|  |  | *zOTU_46 Porphyromonas HOT 930* | CFAT | 9.79E-04 |  |  |
|  |  | *zOTU_2205 Porphyromonas HOT 930* | CFAT | 8.20E-03 |  |  |
|  |  | *zOTU_2174 Streptococcus dentisani/infantis/mitis/oralis/HOT 058/HOT 061/HOT 064/HOT 070/HOT 423/HOT 431/tigurinus* | CFAT | 1.23E-04 |  |  |
|  |  | *zOTU_1719 unclassified Streptococcus* | CFAT | 2.34E-02 |  |  |
|  |  | *zOTU_1249 Streptococcus dentisani/infantis/mitis/oralis/HOT 058/HOT 061/HOT 064/HOT 070/HOT 423/HOT 431/tigurinus* | CFAT | 5.45E-03 |  |  |
|  |  | *zOTU_2132 Streptococcus dentisani/infantis/mitis/oralis/HOT 058/HOT 061/HOT 064/HOT 070/HOT 423/HOT 431/tigurinus* | CFAT | 1.24E-03 |  |  |
|  |  | *zOTU_1222 Streptococcus dentisani/infantis/mitis/oralis/HOT 058/HOT 061/HOT 064/HOT 070/HOT 423/HOT 431/tigurinus* | CFAT | 1.85E-03 |  |  |
|  |  | *zOTU_1027 Streptococcus dentisani/infantis/mitis/oralis/HOT 058/HOT 061/HOT 064/HOT 070/HOT 423/HOT 431/tigurinus* | CFAT | 1.74E-02 |  |  |
|  |  | *zOTU_2235 unclassified Haemophilus* | CFAT | 4.29E-02 |  |  |
|  |  | *zOTU_1492 Porphyromonas HOT 930* | CFAT | 9.17E-03 |  |  |
|  |  | *zOTU_31 unclassified Bergeyella* | CFAT | 3.84E-02 |  |  |
|  |  | *zOTU_1035 unclassified Bergeyella* | CFAT | 4.14E-02 |  |  |
|  |  | *zOTU_1734 Streptococcus dentisani/infantis/mitis/oralis/HOT 058/HOT 061/HOT 064/HOT 070/HOT 423/HOT 431/tigurinus* | CFAT | 4.16E-02 |  |  |
|  |  | *zOTU_2276 Gemella haemolysans/morbillorum/sanguinis* | CFAT | 4.69E-02 |  |  |
|  |  | *zOTU_2407 unclassified Veillonella* | C4 | 1.55E-03 |  |  |
|  |  | *zOTU_1255 Veillonella HOT 780* | C4 | 8.00E-03 |  |  |
|  |  | *zOTU_615 unclassified Veillonella* | C4 | 4.64E-02 |  |  |
|  |  | *zOTU_1854 Alloprevotella HOT 473* | C4 | 1.30E-02 |  |  |
|  |  | *zOTU_10 Veillonella dispar/atypica** | C4 | 1.33E-03 |  |  |
|  |  | *zOTU_828 unclassified Veillonella* | C4 | 1.08E-02 |  |  |
|  |  | *zOTU_1816 unclassified Veillonella* | C4 | 3.10E-02 |  |  |
|  |  | *zOTU_935 unclassified Veillonella* | C4 | 3.87E-02 |  |  |
|  |  | *zOTU_1068 unclassified Veillonella* | C4 | 4.90E-02 |  |  |
|  |  | *zOTU_1088 unclassified Veillonella* | C4 | 4.30E-03 |  |  |
|  |  | *zOTU_490 Veillonella dispar* | C4 | 4.49E-02 |  |  |
|  |  | *zOTU_628 Veillonella dispar* | C4 | 4.54E-02 |  |  |
|  |  | *zOTU_1509 unclassified Veillonella* | C4 | 3.70E-02 |  |  |
|  |  | *zOTU_1873 Actinomyces odontolyticus/HOT 180* | C4 | 1.00E-02 |  |  |
|  |  | *zOTU_124 Rothia mucilaginosa* | C4 | 1.75E-02 |  |  |
|  |  | *zOTU_142 Actinomyces lingnae [NVP]* | C4 | 2.62E-02 |  |  |
|  |  | *zOTU_2040 Actinomyces odontolyticus/HOT 180* | C4 | 3.87E-02 |  |  |
|  |  | *zOTU_2222 unclassified Megasphaera* | C4 | 3.87E-02 |  |  |
|  |  | *zOTU_1828 unclassified Veillonella* | C4 | 4.74E-02 |  |  |
|  |  | *zOTU_2179 Streptococcus salivarius/vestibularis* | C4 | 1.51E-02 |  |  |
|  |  | *zOTU_135 Streptococcus australis/parasanguinis I/parasanguinis II/HOT 057/HOT 066** | C4 | 1.75E-02 |  |  |
|  |  | *zOTU_1855 Streptococcus peroris/lactarius/HOT 074** | C4 | 4.92E-02 |  |  |
|  |  | *zOTU_34 Streptococcus HOT 056/gordonii** | C4 | 1.70E-02 |  |  |
|  |  | *zOTU_47 Streptococcus HOT 056/gordonii** | C4 | 2.93E-02 |  |  |
|  |  | *zOTU_1128 Streptococcus salivarius/vestibularis* | C4 | 4.39E-02 |  |  |
|  |  | *zOTU_353 unclassified Enterococcus* | C4 | 3.41E-02 |  |  |
|  |  | *zOTU_1720 unclassified Veillonella* | C4 | 1.83E-02 |  |  |

*P*-value represents the results from global test.

* indicates zOTUs additionally blasted on HOMD website with similarity ≥ 98.5%.

| **B** | **dental plaque samples in the CFAT vs C4 group at T2** | | | | | |
| --- | --- | --- | --- | --- | --- | --- |
|  | ***n* of zOTU groups** | **Numbers and taxonomical names of the zOTUs at genus and species level** | **Associated group** | ***P*-value of the individual zOTU** | ***P*-value of the group of zOTUs corrected for multiple testing** | **Bonferroni- corrected Overall *P*-value** |
|  | 1 | *zOTU_93 Leptotrichia wadei* | C4 | 2.33E-05 | 4.85E-02 | 6.84E-04 |
|  |  | *zOTU_1753 unclassified Leptotrichia* | C4 | 3.62E-03 |  |  |
|  |  | *zOTU_2172 unclassified Leptotrichia* | C4 | 1.50E-02 |  |  |
|  |  | *zOTU_2098 unclassified Leptotrichia* | C4 | 1.89E-02 |  |  |
|  |  | *zOTU_1237 Leptotrichia HOT 417 ** | C4 | 4.33E-02 |  |  |
|  |  | *zOTU_1053 unclassified Leptotrichia* | C4 | 4.91E-03 |  |  |
|  |  | *zOTU_848 unclassified Leptotrichia* | C4 | 6.43E-03 |  |  |
|  |  | *zOTU_1429 unclassified Leptotrichia* | C4 | 3.69E-02 |  |  |
|  |  | *zOTU_40 Leptotrichia HOT 417 ** | C4 | 1.27E-02 |  |  |
|  |  | *zOTU_926 unclassified Leptotrichia* | C4 | 9.84E-03 |  |  |
|  |  | *zOTU_144 Actinomyces georgiae/HOT 178/HOT 877* | C4 | 3.61E-02 |  |  |
|  |  | *zOTU_2339 unclassified Actinomyces* | C4 | 4.01E-02 |  |  |
|  |  | *zOTU_1293 unclassified Leptotrichia* | C4 | 9.51E-03 |  |  |
|  |  | *zOTU_201 Stomatobaculum longum* | C4 | 2.17E-02 |  |  |
|  |  | *zOTU_367 Capnocytophaga HOT 412/HOT 902/HOT 380/HOT 323** | C4 | 2.92E-02 |  |  |
|  | 2 | *zOTU_2229 unclassified Veillonella* | C4 | 4.50E-05 | 3.37E-02 |  |
|  |  | *zOTU_831 Veillonella dispar* | C4 | 1.80E-04 |  |  |
|  |  | *zOTU_1287 Corynebacterium matruchotii* | C4 | 3.88E-03 |  |  |
|  |  | *zOTU_2282 unclassified Veillonella* | C4 | 4.80E-04 |  |  |
|  |  | *zOTU_1583 Corynebacterium matruchotii* | C4 | 4.59E-03 |  |  |
|  |  | *zOTU_1557 Corynebacterium matruchotii* | C4 | 1.58E-02 |  |  |
|  |  | *zOTU_869 Veillonella dispar/atypica** | C4 | 7.55E-04 |  |  |
|  |  | *zOTU_1509 unclassified Veillonella* | C4 | 4.93E-03 |  |  |
|  |  | *zOTU_1411 Veillonella dispar* | C4 | 1.47E-02 |  |  |
|  |  | *zOTU_2318 Corynebacterium matruchotii* | C4 | 4.37E-03 |  |  |
|  |  | *zOTU_1655 Corynebacterium matruchotii* | C4 | 2.06E-02 |  |  |
|  |  | *zOTU_1881 Corynebacterium matruchotii* | C4 | 4.35E-02 |  |  |
|  |  | *zOTU_1443 Corynebacterium matruchotii* | C4 | 3.21E-02 |  |  |
|  |  | *zOTU_2226 Corynebacterium matruchotii* | C4 | 7.92E-03 |  |  |
|  |  | *zOTU_2051 Leptotrichia shahii* | C4 | 8.59E-04 |  |  |
|  |  | *zOTU_2010 Leptotrichia shahii* | C4 | 5.06E-03 |  |  |
|  |  | *zOTU_25 Leptotrichia shahii* | C4 | 1.81E-02 |  |  |
|  |  | *zOTU_1440 Leptotrichia hongkongensis* | C4 | 4.69E-03 |  |  |
|  |  | *zOTU_2398 unclassified Leptotrichia* | C4 | 8.10E-03 |  |  |
|  |  | *zOTU_971 Leptotrichia shahii* | C4 | 9.50E-03 |  |  |
|  |  | *zOTU_1130 Leptotrichia shahii* | C4 | 1.79E-02 |  |  |
|  |  | *zOTU_174 Lachnoanaerobaculum saburreum* | C4 | 1.85E-03 |  |  |
|  |  | *zOTU_234 Lachnoanaerobaculum saburreum* | C4 | 3.65E-03 |  |  |
|  |  | *zOTU_1078 Leptotrichia hongkongensis* | C4 | 1.27E-02 |  |  |
|  |  | *zOTU_2072 Leptotrichia hongkongensis* | C4 | 2.73E-02 |  |  |
|  | 3 | *zOTU_55 Streptococcus mutans* | C4 | 2.76E-05 | 5.39E-03 |  |
|  | 4 | *zOTU_1765 Streptococcus mutans* | C4 | 1.86E-04 | 4.52E-02 |  |
|  |  | *zOTU_1519 Streptococcus mutans* | C4 | 2.46E-04 |  |  |
|  |  | *zOTU_1600 Streptococcus mutans* | C4 | 2.77E-04 |  |  |
|  |  | *zOTU_1717 Streptococcus mutans* | C4 | 4.13E-04 |  |  |
|  | 5 | *zOTU_1622 unclassified Streptococcus* | C4 | 3.08E-05 | 1.25E-02 |  |
|  | 6 | *zOTU_103 Leptotrichia hofstadii** | C4 | 4.89E-04 | 4.90E-02 |  |
|  |  | *zOTU_311 Leptotrichia hofstadii** | C4 | 7.57E-04 |  |  |
|  |  | *zOTU_178 Leptotrichia hofstadii** | C4 | 2.93E-03 |  |  |
|  |  | *zOTU_2088 Leptotrichia HOT 909* | C4 | 3.86E-03 |  |  |
|  | 7 | *zOTU_490 Veillonella dispar* | C4 | 1.67E-04 | 3.71E-02 |  |
|  |  | *zOTU_591 unclassified Actinomyces* | C4 | 6.91E-04 |  |  |
|  |  | *zOTU_744 unclassified Actinomyces* | C4 | 1.48E-03 |  |  |
|  |  | *zOTU_514 Veillonella dispar/atypica** | C4 | 6.10E-03 |  |  |
|  |  | *zOTU_411 unclassified Veillonella* | C4 | 6.98E-03 |  |  |
|  |  | *zOTU_456 unclassified Veillonella* | C4 | 1.68E-02 |  |  |
|  |  | *zOTU_309 Streptococcus dentisani/infantis/mitis/oralis/HOT 058/HOT 061/HOT 064/HOT 070/HOT 423/HOT 431/tigurinus* | C4 | 1.66E-02 |  |  |
|  |  | *zOTU_1816 unclassified Veillonella* | C4 | 1.27E-02 |  |  |
|  |  | *zOTU_828 unclassified Veillonella* | C4 | 7.25E-03 |  |  |
|  |  | *zOTU_15 Veillonella dispar* | C4 | 9.74E-03 |  |  |
|  |  | *zOTU_1490 Streptococcus dentisani/infantis/mitis/oralis/HOT 058/HOT 061/HOT 064/HOT 070/HOT 423/HOT 431/tigurinus* | C4 | 9.63E-03 |  |  |
|  |  | *zOTU_1179 unclassified Veillonella* | C4 | 3.78E-04 |  |  |
|  |  | *zOTU_628 Veillonella dispar* | C4 | 7.00E-04 |  |  |
|  |  | *zOTU_781 Veillonella dispar* | C4 | 2.34E-02 |  |  |
|  |  | *zOTU_708 Veillonella dispar* | C4 | 2.55E-03 |  |  |
|  |  | *zOTU_2042 unclassified Streptococcus* | C4 | 3.10E-02 |  |  |
|  |  | *zOTU_2165 unclassified Veillonella* | C4 | 1.11E-02 |  |  |
|  |  | *zOTU_2100 Streptococcus dentisani/infantis/mitis/oralis/HOT 058/HOT 061/HOT 064/HOT 070/HOT 423/HOT 431/tigurinus* | C4 | 9.60E-03 |  |  |
|  |  | *zOTU_1465 Streptococcus dentisani/infantis/mitis/oralis/HOT 058/HOT 061/HOT 064/HOT 070/HOT 423/HOT 431/tigurinus* | C4 | 2.95E-02 |  |  |
|  |  | *zOTU_1353 Veillonella dispar* | C4 | 2.49E-02 |  |  |
|  |  | *zOTU_2335 Veillonella dispar* | C4 | 2.26E-04 |  |  |
|  |  | *zOTU_1506 Veillonella dispar* | C4 | 7.59E-03 |  |  |
|  |  | *zOTU_1936 unclassified Actinomyces* | C4 | 5.26E-03 |  |  |
|  |  | *zOTU_1584 unclassified Veillonella* | C4 | 1.77E-02 |  |  |
|  |  | *zOTU_2289 Veillonella parvula* | C4 | 2.13E-02 |  |  |
|  |  | *zOTU_1114 Veillonella dispar* | C4 | 6.65E-03 |  |  |
|  |  | *zOTU_44 Kingella oralis* | C4 | 3.90E-03 |  |  |
|  |  | *zOTU_1576 unclassified Kingella* | C4 | 3.12E-02 |  |  |
|  |  | *zOTU_1847 unclassified Neisseria* | C4 | 7.75E-03 |  |  |
|  |  | *zOTU_1354 unclassified Neisseria* | C4 | 2.99E-02 |  |  |
|  |  | *zOTU_2389 Neisseria pharyngis/sicca/subflava/mucosa/flavescens/flava** | C4 | 4.29E-02 |  |  |
|  | 8 | *zOTU_1088 unclassified Veillonella* | C4 | 3.20E-04 | 2.73E-02 |  |
|  |  | *zOTU_1579 Corynebacterium durum* | C4 | 6.84E-04 |  |  |
|  |  | *zOTU_1259 Veillonella dispar* | C4 | 7.77E-04 |  |  |
|  |  | *zOTU_2262 Corynebacterium durum* | C4 | 1.23E-02 |  |  |
|  | 9 | *zOTU_24 Leptotrichia hongkongensis* | C4 | 4.12E-04 | 5.89E-03 |  |
|  |  | *zOTU_1165 Leptotrichia hongkongensis* | C4 | 4.13E-03 |  |  |
|  |  | *zOTU_1940 Leptotrichia hongkongensis* | C4 | 1.24E-02 |  |  |
|  |  | *zOTU_943 Leptotrichia hongkongensis* | C4 | 1.80E-03 |  |  |
|  |  | *zOTU_1515 unclassified Leptotrichia* | C4 | 4.82E-02 |  |  |
|  |  | *zOTU_1908 Leptotrichia hongkongensis* | C4 | 3.20E-02 |  |  |
|  |  | *zOTU_1548 Streptococcus HOT 056/gordonii** | C4 | 4.59E-04 |  |  |
|  |  | *zOTU_34 Streptococcus HOT 056/gordonii** | C4 | 1.36E-03 |  |  |
|  |  | *zOTU_125 Leptotrichia HOT 221* | C4 | 2.08E-03 |  |  |
|  |  | *zOTU_136 Lachnoanaerobaculum orale** | C4 | 2.27E-02 |  |  |
|  | 10 | *zOTU_20 Lautropia mirabilis* | CFAT | 2.14E-04 | 4.28E-02 |  |
|  |  | *zOTU_2239 Lautropia mirabilis* | CFAT | 8.82E-03 |  |  |
|  |  | *zOTU_1257 unclassified Aggregatibacter/Haemophilus** | CFAT | 1.10E-02 |  |  |
|  |  | *zOTU_1955 unclassified Neisseria* | CFAT | 6.00E-04 |  |  |
|  |  | *zOTU_454 Lautropia mirabilis* | CFAT | 9.90E-04 |  |  |
|  |  | *zOTU_1426 unclassified Neisseria* | CFAT | 1.04E-03 |  |  |
|  |  | *zOTU_1109 unclassified Neisseria* | CFAT | 1.92E-02 |  |  |
|  |  | *zOTU_920 Neisseria flava/mucosa/pharyngis/sicca** | CFAT | 4.03E-02 |  |  |
|  |  | *zOTU_704 unclassified Neisseria* | CFAT | 2.26E-02 |  |  |
|  |  | *zOTU_1186 Lautropia mirabilis* | CFAT | 2.36E-02 |  |  |
|  |  | *zOTU_511 Neisseria flavescens/subflava* | CFAT | 2.77E-03 |  |  |
|  |  | *zOTU_480 unclassified Neisseria* | CFAT | 4.57E-03 |  |  |
|  |  | *zOTU_550 unclassified Neisseria* | CFAT | 1.34E-02 |  |  |
|  |  | *zOTU_2107 unclassified Neisseria* | CFAT | 9.51E-03 |  |  |
|  |  | *zOTU_1326 Haemophilus parainfluenzae* | CFAT | 2.76E-02 |  |  |
|  |  | *zOTU_36 Abiotrophia defectiva* | CFAT | 2.46E-03 |  |  |
|  |  | *zOTU_1964 Abiotrophia defectiva* | CFAT | 4.23E-02 |  |  |
|  |  | *zOTU_200 unclassified Streptococcus* | CFAT | 1.57E-02 |  |  |
|  |  | *zOTU_772 Abiotrophia defectiva* | CFAT | 3.93E-02 |  |  |
|  |  | *zOTU_1199 unclassified Streptococcus* | CFAT | 1.96E-02 |  |  |
|  |  | *zOTU_1808 Neisseria flava/mucosa/pharyngis/sicca** | CFAT | 3.99E-02 |  |  |
|  |  | *zOTU_2223 Lautropia mirabilis* | CFAT | 1.09E-02 |  |  |
|  |  | *zOTU_1047 Kingella denitrificans / Neisseria elongata* | CFAT | 3.41E-02 |  |  |
|  |  | *zOTU_776 unclassified Actinomyces* | CFAT | 1.33E-02 |  |  |
|  |  | *zOTU_596 Streptococcus sanguinis* | CFAT | 2.24E-02 |  |  |
|  |  | *zOTU_4 Streptococcus sanguinis* | CFAT | 2.67E-02 |  |  |
|  |  | *zOTU_65 Bergeyella HOT 322* | CFAT | 3.12E-04 |  |  |
|  | 11 | *zOTU_2220 unclassified Neisseria* | CFAT | 1.44E-04 | 4.75E-02 |  |
|  |  | *zOTU_1070 unclassified Neisseria* | CFAT | 4.79E-03 |  |  |
|  |  | *zOTU_58 Neisseria oralis* | CFAT | 2.37E-03 |  |  |
|  |  | *zOTU_2394 Neisseria oralis* | CFAT | 1.12E-02 |  |  |
|  |  | *zOTU_1481 Haemophilus parainfluenzae* | CFAT | 4.65E-02 |  |  |
|  |  | *zOTU_1708 Neisseria weaveri* | CFAT | 1.58E-03 |  |  |

*P*-value represents the results from global test.

* indicates zOTUs additionally blasted on HOMD website with similarity ≥ 98.5%.

| **C** | **dental plaque samples in the CFAT vs EC group at T2** | | | | | |
| --- | --- | --- | --- | --- | --- | --- |
|  | ***n* of zOTU groups** | **Numbers and taxonomical names of the zOTUs at genus and species level** | **Associated group** | ***P*-value of the individual zOTU** | ***P*-value of the group of zOTUs corrected for multiple testing** | **Bonferroni- corrected Overall *P*-value** |
|  | 1 | *zOTU_65 Bergeyella HOT 322* | CFAT | 1.15E-05 | 5.84E-03 | 1.98E-02 |
|  | 2 | *zOTU_36 Abiotrophia defectiva* | CFAT | 1.77E-05 | 9.89E-03 |  |
|  | 3 | *zOTU_870 unclassified Streptococcus* | CFAT | 6.27E-04 | 2.55E-02 |  |
|  |  | *zOTU_200 unclassified Streptococcus* | CFAT | 1.07E-03 |  |  |
|  |  | *zOTU_52 Streptococcus sanguinis** | CFAT | 6.62E-03 |  |  |
|  |  | *zOTU_32 Streptococcus dentisani/infantis/mitis/oralis/HOT 058/HOT 061/HOT 064/HOT 070/HOT 423/HOT 431/tigurinus** | CFAT | 7.09E-03 |  |  |
|  |  | *zOTU_1326 Haemophilus parainfluenzae* | CFAT | 1.20E-03 |  |  |
|  |  | *zOTU_3 Haemophilus parainfluenzae* | CFAT | 1.29E-03 |  |  |
|  |  | *zOTU_1279 Haemophilus parainfluenzae* | CFAT | 1.36E-03 |  |  |
|  |  | *zOTU_1664 Haemophilus parainfluenzae* | CFAT | 9.41E-03 |  |  |
|  |  | *zOTU_663 Haemophilus parainfluenzae* | CFAT | 4.72E-02 |  |  |
|  |  | *zOTU_1944 Streptococcus dentisani/infantis/mitis/oralis/HOT 058/HOT 061/HOT 064/HOT 070/HOT 423/HOT 431/tigurinus* | CFAT | 1.58E-03 |  |  |
|  |  | *zOTU_1257 unclassified Aggregatibacter/Haemophilus** | CFAT | 2.23E-03 |  |  |
|  |  | *zOTU_1838 Haemophilus parainfluenzae* | CFAT | 2.47E-02 |  |  |
|  | 4 | *zOTU_550 unclassified Neisseria* | CFAT | 9.41E-05 | 2.55E-02 |  |
|  |  | *zOTU_480 unclassified Neisseria* | CFAT | 2.20E-04 |  |  |
|  | 5 | *zOTU_1426 unclassified Neisseria* | CFAT | 2.22E-04 | 3.62E-02 |  |
|  |  | *zOTU_1955 unclassified Neisseria* | CFAT | 8.79E-04 |  |  |
|  |  | *zOTU_454 Lautropia mirabilis* | CFAT | 1.12E-03 |  |  |
|  |  | *zOTU_704 unclassified Neisseria* | CFAT | 2.04E-02 |  |  |
|  |  | *zOTU_1109 unclassified Neisseria* | CFAT | 2.69E-03 |  |  |
|  |  | *zOTU_920 Neisseria flava/mucosa/pharyngis/sicca** | CFAT | 1.12E-02 |  |  |
|  |  | *zOTU_20 Lautropia mirabilis* | CFAT | 2.41E-04 |  |  |
|  | 6 | *zOTU_189 Leptotrichia HOT 219* | CFAT | 1.84E-05 | 2.64E-02 |  |
|  |  | *zOTU_171 Capnocytophaga HOT 338* | CFAT | 1.31E-02 |  |  |
|  |  | *zOTU_73 Aggregatibacter aphrophilus/paraphrophilus* | CFAT | 1.16E-03 |  |  |
|  |  | *zOTU_242 unclassified Aggregatibacter/Haemophilus** | CFAT | 4.27E-03 |  |  |
|  |  | *zOTU_205 unclassified Aggregatibacter/Haemophilus** | CFAT | 4.05E-02 |  |  |
|  |  | *zOTU_1080 unclassified Aggregatibacter* | CFAT | 2.43E-02 |  |  |
|  |  | *zOTU_978 unclassified Aggregatibacter* | CFAT | 2.78E-02 |  |  |
|  |  | *zOTU_1599 unclassified Aggregatibacter* | CFAT | 4.02E-02 |  |  |
|  |  | *zOTU_2025 unclassified Neisseria* | CFAT | 4.67E-02 |  |  |
|  |  | *zOTU_108 Porphyromonas catoniae/HOT 275/HOT 277/HOT 284* | CFAT | 8.70E-04 |  |  |
|  |  | *zOTU_1052 unclassified Neisseria* | CFAT | 1.27E-02 |  |  |
|  |  | *zOTU_1692 Lautropia mirabilis* | CFAT | 4.64E-02 |  |  |
|  |  | *zOTU_1788 unclassified Haemophilus* | CFAT | 3.56E-02 |  |  |
|  |  | *zOTU_74 Capnocytophaga gingivalis* | CFAT | 1.30E-03 |  |  |
|  |  | *zOTU_280 GN02_[G-1] HOT 872* | CFAT | 3.88E-02 |  |  |
|  |  | *zOTU_2061 Fusobacterium HOT 203/nucleatum subsp. polymorphum** | CFAT | 1.27E-02 |  |  |
|  |  | *zOTU_923 Fusobacterium HOT 203/nucleatum subsp. nucleatum** | CFAT | 2.39E-02 |  |  |
|  |  | *zOTU_2285 unclassified Fusobacterium* | CFAT | 3.74E-02 |  |  |
|  |  | *zOTU_29 Capnocytophaga sputigena* | CFAT | 2.15E-02 |  |  |
|  |  | *zOTU_301 Lachnospiraceae_[G-3] HOT 100* | CFAT | 2.47E-02 |  |  |
|  |  | *zOTU_909 Porphyromonas pasteri/HOT 278* | EC | 4.51E-02 |  |  |
|  |  | *zOTU_173 Cardiobacterium valvarum* | CFAT | 1.64E-02 |  |  |
|  |  | *zOTU_2302 Lachnospiraceae_[G-3] HOT 100* | CFAT | 2.72E-02 |  |  |
|  |  | *zOTU_778 Propionibacterium propionicum* | CFAT | 2.29E-02 |  |  |
|  |  | *zOTU_119 Prevotella HOT 472/loescheii** | CFAT | 4.39E-02 |  |  |
|  |  | *zOTU_1560 unclassified Actinomyces* | CFAT | 3.88E-05 |  |  |
|  |  | *zOTU_1954 Leptotrichia HOT 215* | CFAT | 2.72E-02 |  |  |
|  |  | *zOTU_996 Porphyromonas pasteri/HOT 278* | CFAT | 3.29E-02 |  |  |
|  |  | *zOTU_2078 unclassified Haemophilus* | CFAT | 6.53E-03 |  |  |
|  |  | *zOTU_2363 unclassified Haemophilus* | CFAT | 7.22E-03 |  |  |
|  |  | *zOTU_1217 Porphyromonas pasteri/HOT 278* | EC | 4.95E-02 |  |  |
|  |  | *zOTU_55 Streptococcus mutans* | EC | 1.62E-03 |  |  |
|  |  | *zOTU_1205 unclassified Streptococcus* | EC | 1.19E-02 |  |  |
|  |  | *zOTU_2335 Veillonella dispar* | EC | 1.49E-02 |  |  |
|  |  | *zOTU_1509 unclassified Veillonella* | EC | 3.82E-02 |  |  |
|  |  | *zOTU_1936 unclassified Actinomyces* | EC | 4.33E-02 |  |  |
|  |  | *zOTU_490 Veillonella dispar* | EC | 1.78E-02 |  |  |
|  |  | *zOTU_591 unclassified Actinomyces* | EC | 4.73E-02 |  |  |
|  |  | *zOTU_628 Veillonella dispar* | EC | 3.16E-02 |  |  |
|  |  | *zOTU_1179 unclassified Veillonella* | EC | 4.36E-02 |  |  |
|  |  | *zOTU_2229 unclassified Veillonella* | EC | 2.14E-02 |  |  |
|  |  | *zOTU_2282 unclassified Veillonella* | EC | 2.41E-02 |  |  |
|  |  | *zOTU_1088 unclassified Veillonella* | EC | 3.21E-02 |  |  |
|  |  | *zOTU_1259 Veillonella dispar* | EC | 4.04E-02 |  |  |
|  |  | *zOTU_93 Leptotrichia wadei* | EC | 1.69E-03 |  |  |
|  |  | *zOTU_1053 unclassified Leptotrichia* | EC | 4.82E-02 |  |  |
|  |  | *zOTU_1687 unclassified Selenomonas* | EC | 3.53E-02 |  |  |
|  |  | *zOTU_234 Lachnoanaerobaculum saburreum* | EC | 4.65E-02 |  |  |
|  |  | *zOTU_1398 unclassified Veillonella* | EC | 3.30E-02 |  |  |
|  |  | *zOTU_831 Veillonella dispar* | EC | 3.88E-02 |  |  |
|  |  | *zOTU_169 Selenomonas HOT 136/HOT 149/HOT 478* | EC | 9.25E-03 |  |  |
|  |  | *zOTU_136 Lachnoanaerobaculum orale** | EC | 1.48E-02 |  |  |
|  |  | *zOTU_89 Campylobacter concisus** | EC | 3.80E-02 |  |  |
|  |  | *zOTU_76 Prevotella HOT 317* | EC | 4.88E-02 |  |  |
|  |  | *zOTU_232 Actinomyces dentalis* | EC | 1.30E-02 |  |  |
|  |  | *zOTU_248 TM7_[G-1] HOT 346* | EC | 4.55E-02 |  |  |
|  |  | *zOTU_363 Oribacterium HOT 078* | EC | 2.55E-02 |  |  |
|  |  | *zOTU_125 Leptotrichia HOT 221* | EC | 5.60E-03 |  |  |
|  |  | *zOTU_1580 unclassified Actinomyces* | CFAT | 2.67E-02 |  |  |
|  |  | *zOTU_2072 Leptotrichia hongkongensis* | EC | 2.47E-02 |  |  |
|  |  | *zOTU_1078 Leptotrichia hongkongensis* | EC | 4.97E-02 |  |  |
|  |  | *zOTU_943 Leptotrichia hongkongensis* | EC | 3.73E-02 |  |  |
|  |  | *zOTU_1165 Leptotrichia hongkongensis* | EC | 4.42E-02 |  |  |
|  |  | *zOTU_1940 Leptotrichia hongkongensis* | EC | 4.52E-02 |  |  |
|  |  | *zOTU_128 Actinomyces gerencseriae** | EC | 7.11E-03 |  |  |
|  |  | *zOTU_113 Fusobacterium nucleatum subsp. animalis* | EC | 2.12E-02 |  |  |
|  |  | *zOTU_317 Actinomyces HOT 448* | EC | 3.05E-02 |  |  |
|  |  | *zOTU_98 unclassified Capnocytophaga* | CFAT | 2.70E-02 |  |  |
|  |  | *zOTU_1340 unclassified Streptococcus* | CFAT | 3.49E-02 |  |  |
|  |  | *zOTU_1712 unclassified Streptococcus* | CFAT | 1.17E-02 |  |  |
|  |  | *zOTU_16 Rothia dentocariosa* | EC | 1.40E-02 |  |  |
|  |  | *zOTU_1382 unclassified Actinomyces/Rothia** | EC | 2.19E-02 |  |  |
|  |  | *zOTU_1649 unclassified Actinomyces/Rothia** | EC | 4.84E-02 |  |  |
|  |  | *zOTU_992 Streptococcus dentisani/infantis/mitis/oralis/HOT 058/HOT 061/HOT 064/HOT 070/HOT 423/HOT 431/tigurinus* | EC | 4.35E-02 |  |  |
|  |  | *zOTU_1548 Streptococcus HOT 056/gordonii** | EC | 4.62E-02 |  |  |

*P*-value represents the results from global test.

* indicates zOTUs additionally blasted on HOMD website with similarity ≥ 98.5%.

**Appendix Table 3:** zOTUs contributing to the difference in the microbiome composition of the saliva **(ABC)** and the dental plaque **(DEF)** samples of the children at T3. Only zOTUs with individual *P*<0.05 are shown.

| **A** | **saliva samples in the CFAT vs C6.5 group at T3** | | | | | |
| --- | --- | --- | --- | --- | --- | --- |
|  | ***n* of zOTU groups** | **Numbers and taxonomical names of the zOTUs at genus and species level** | **Associated group** | ***P*-value of the individual zOTU** | ***P*-value of the group of zOTUs corrected for multiple testing** | **Bonferroni- corrected Overall *P*-value** |
|  | 1 | *zOTU_23 Prevotella histicola** | C6.5 | 7.61E-08 | 6.22E-03 | 1.02E-02 |
|  | 2 | *zOTU_475 Prevotella melaninogenica** | C6.5 | 1.14E-04 | 3.85E-02 |  |
|  |  | *zOTU_120 Prevotella HOT 313** | C6.5 | 5.37E-03 |  |  |
|  |  | *zOTU_709 unclassified Prevotella* | C6.5 | 1.25E-03 |  |  |
|  |  | *zOTU_165 Prevotella veroralis* | C6.5 | 6.64E-03 |  |  |
|  |  | *zOTU_68 Prevotella salivae* | C6.5 | 1.56E-04 |  |  |
|  |  | *zOTU_239 Prevotella salivae* | C6.5 | 9.08E-03 |  |  |
|  |  | *zOTU_544 Prevotella salivae* | C6.5 | 1.08E-02 |  |  |
|  |  | *zOTU_549 Prevotella salivae* | C6.5 | 1.58E-02 |  |  |
|  | 3 | *zOTU_1429 unclassified Leptotrichia* | C6.5 | 6.60E-05 | 4.19E-02 |  |
|  |  | *zOTU_1508 unclassified Leptotrichia* | C6.5 | 3.02E-02 |  |  |
|  |  | *zOTU_93 Leptotrichia wadei* | C6.5 | 1.52E-04 |  |  |
|  |  | *zOTU_1053 unclassified Leptotrichia* | C6.5 | 2.21E-02 |  |  |
|  |  | *zOTU_848 unclassified Leptotrichia* | C6.5 | 2.67E-02 |  |  |
|  | 4 | *zOTU_40 Leptotrichia HOT 417** | C6.5 | 2.77E-04 | 4.00E-02 |  |
|  |  | *zOTU_1115 unclassified Leptotrichia* | C6.5 | 1.49E-03 |  |  |
|  |  | *zOTU_1562 unclassified Fusobacteriales* | C6.5 | 3.11E-03 |  |  |
|  |  | *zOTU_1237 Leptotrichia HOT 417** | C6.5 | 6.90E-03 |  |  |
|  |  | *zOTU_1073 Leptotrichia HOT 417** | C6.5 | 4.43E-03 |  |  |

*P*-value represents the results from global test.

* indicates zOTUs additionally blasted on HOMD website with similarity ≥ 98.5%.

| **B** | **dental plaque samples in the CFAT vs C6.5 group at T3** | | | | | |
| --- | --- | --- | --- | --- | --- | --- |
|  | ***n* of zOTU groups** | **Numbers and taxonomical names of the zOTUs at genus and species level** | **Associated group** | ***P*-value of the individual zOTU** | ***P*-value of the group of zOTUs corrected for multiple testing** | **Bonferroni- corrected Overall *P*-value** |
|  | 1 | *zOTU_93 Leptotrichia wadei* | C6.5 | 2.31E-06 | 8.86E-04 | 7.30E-04 |
|  | 2 | *zOTU_1562 unclassified Fusobacteriales* | C6.5 | 1.41E-04 | 3.95E-02 |  |
|  |  | *zOTU_2325 unclassified Leptotrichia* | C6.5 | 3.72E-04 |  |  |
|  |  | *zOTU_926 unclassified Leptotrichia* | C6.5 | 1.90E-03 |  |  |
|  |  | *zOTU_1115 unclassified Leptotrichia* | C6.5 | 2.29E-04 |  |  |
|  |  | *zOTU_1053 unclassified Leptotrichia* | C6.5 | 3.11E-04 |  |  |
|  |  | *zOTU_1429 unclassified Leptotrichia* | C6.5 | 4.33E-03 |  |  |
|  |  | *zOTU_848 unclassified Leptotrichia* | C6.5 | 9.67E-04 |  |  |
|  |  | *zOTU_974 unclassified Leptotrichia* | C6.5 | 2.59E-03 |  |  |
|  |  | *zOTU_2228 Leptotrichia HOT 417** | C6.5 | 1.38E-03 |  |  |
|  |  | *zOTU_1227 Leptotrichia HOT 225* | C6.5 | 4.67E-03 |  |  |
|  | 3 | *zOTU_1237 Leptotrichia HOT 417** | C6.5 | 1.05E-04 | 3.60E-02 |  |
|  |  | *zOTU_1660 Leptotrichia HOT 417** | C6.5 | 1.31E-03 |  |  |
|  |  | *zOTU_951 unclassified Leptotrichia* | C6.5 | 7.28E-04 |  |  |
|  |  | *zOTU_40 Leptotrichia HOT 417** | C6.5 | 9.90E-04 |  |  |
|  | 4 | *zOTU_1154 Prevotella HOT 317* | C6.5 | 2.58E-05 | 2.60E-02 |  |
|  |  | *zOTU_1563 Prevotella HOT 317* | C6.5 | 1.92E-04 |  |  |
|  |  | *zOTU_1726 unclassified Prevotella* | C6.5 | 7.60E-04 |  |  |
|  |  | *zOTU_1818 unclassified Prevotella* | C6.5 | 8.17E-04 |  |  |
|  |  | *zOTU_1101 unclassified Prevotella* | C6.5 | 3.82E-05 |  |  |
|  | 5 | *zOTU_311 Leptotrichia hofstadii** | C6.5 | 7.65E-05 | 2.90E-02 |  |

*P*-value represents the results from global test.

* indicates zOTUs additionally blasted on HOMD website with similarity ≥ 98.5%.

| **C** | **saliva samples in the CFAT vs EC group at T3** | | | | | |
| --- | --- | --- | --- | --- | --- | --- |
|  | ***n* of zOTU groups** | **Numbers and taxonomical names of the zOTUs at genus and species level** | **Associated group** | ***P*-value of the individual zOTU** | ***P*-value of the group of zOTUs corrected for multiple testing** | **Bonferroni- corrected Overall *P*-value** |
|  | 1 | *zOTU_183 Campylobacter concisus* | EC | 8.04E-05 | 4.59E-02 | 9.50E-03 |
|  |  | *zOTU_398 unclassified Streptococcus* | EC | 7.99E-03 |  |  |
|  |  | *zOTU_1076 unclassified Selenomonas* | EC | 1.32E-02 |  |  |
|  |  | *zOTU_2039 Selenomonas infelix/HOT 126/HOT 138/HOT 146/HOT 479/HOT 481/HOT 892/HOT 919/HOT 936* | EC | 1.86E-02 |  |  |
|  |  | *zOTU_2076 Selenomonas infelix/HOT 126/HOT 138/HOT 146/HOT 479/HOT 481/HOT 892/HOT 919/HOT 936* | EC | 2.33E-02 |  |  |
|  |  | *zOTU_1464 unclassified Selenomonas* | EC | 4.39E-02 |  |  |
|  |  | *zOTU_150 Selenomonas noxia** | EC | 1.87E-02 |  |  |
|  |  | *zOTU_1802 unclassified Selenomonas* | EC | 1.11E-02 |  |  |
|  |  | *zOTU_76 Prevotella HOT 317* | EC | 3.67E-02 |  |  |
|  |  | *zOTU_128 Actinomyces gerencseriae** | EC | 7.98E-03 |  |  |
|  |  | *zOTU_174 Lachnoanaerobaculum saburreum* | EC | 2.30E-02 |  |  |
|  |  | *zOTU_25 Leptotrichia shahii* | EC | 8.62E-05 |  |  |
|  |  | *zOTU_676 unclassified Leptotrichia* | EC | 6.51E-03 |  |  |
|  |  | *zOTU_2010 Leptotrichia shahii* | EC | 3.69E-02 |  |  |
|  |  | *zOTU_2398 unclassified Leptotrichia* | EC | 3.73E-02 |  |  |
|  |  | *zOTU_1094 unclassified Leptotrichia* | EC | 2.84E-02 |  |  |
|  |  | *zOTU_805 unclassified Leptotrichia* | EC | 4.52E-02 |  |  |
|  |  | *zOTU_1494 Leptotrichia hongkongensis* | EC | 4.90E-02 |  |  |
|  |  | *zOTU_143 Leptotrichia HOT 498* | EC | 9.72E-04 |  |  |
|  |  | *zOTU_399 Leptotrichia HOT 498* | EC | 2.89E-03 |  |  |
|  |  | *zOTU_151 Prevotella oulorum** | EC | 1.73E-03 |  |  |
|  |  | *zOTU_396 Actinomyces HOT 896* | EC | 3.75E-02 |  |  |
|  |  | *zOTU_845 unclassified Leptotrichia* | EC | 2.11E-02 |  |  |
|  | 2 | *zOTU_93 Leptotrichia wadei* | EC | 9.05E-05 | 2.76E-02 |  |

*P*-value represents the results from global test.

* indicates zOTUs additionally blasted on HOMD website with similarity ≥ 98.5%.

| **D** | **saliva samples in the CFAT vs AC group at T3** | | | | | |
| --- | --- | --- | --- | --- | --- | --- |
|  | ***n* of zOTU groups** | **Numbers and taxonomical names of the zOTUs at genus and species level** | **Associated group** | ***P*-value of the individual zOTU** | ***P*-value of the group of zOTUs corrected for multiple testing** | **Bonferroni- corrected Overall *P*-value** |
|  | 1 | *zOTU_93 Leptotrichia wadei* | AC | 4.23E-07 | 1.59E-04 | 4.10E-06 |
|  | 2 | *zOTU_1508 unclassified Leptotrichia* | AC | 4.37E-06 | 1.36E-02 |  |
|  | 3 | *zOTU_40 Leptotrichia HOT 417** | AC | 7.67E-05 | 2.03E-02 |  |
|  | 4 | *zOTU_174 Lachnoanaerobaculum saburreum* | AC | 1.31E-05 | 1.38E-02 |  |
|  | 5 | *zOTU_55 Streptococcus mutans* | AC | 2.78E-05 | 2.88E-02 |  |
|  | 6 | *zOTU_44 Kingella oralis* | AC | 6.70E-06 | 4.18E-03 |  |
|  | 7 | *zOTU_16 Rothia dentocariosa* | AC | 1.23E-06 | 3.84E-02 |  |
|  | 8 | *zOTU_25 Leptotrichia shahii* | AC | 2.17E-06 | 1.48E-02 |  |
|  | 9 | *zOTU_143 Leptotrichia HOT 498* | AC | 4.45E-06 | 2.65E-03 |  |
|  | 10 | *zOTU_24 Leptotrichia hongkongensis* | AC | 1.31E-05 | 4.12E-03 |  |
|  | 11 | *zOTU_49 Aggregatibacter segnis/HOT 458/HOT 512* | AC | 1.23E-05 | 4.29E-03 |  |
|  | 12 | *zOTU_113 Fusobacterium nucleatum subsp. animalis* | AC | 2.65E-06 | 1.66E-02 |  |
|  |  | *zOTU_102 Fusobacterium nucleatum subsp. animalis* | AC | 7.49E-03 |  |  |
|  |  | *zOTU_105 Streptococcus intermedius* | AC | 6.93E-03 |  |  |
|  |  | *zOTU_151 Prevotella oulorum** | AC | 9.07E-05 |  |  |
|  |  | *zOTU_671 unclassified Prevotella* | AC | 1.81E-02 |  |  |
|  |  | *zOTU_475 Prevotella melaninogenica** | AC | 4.98E-03 |  |  |
|  |  | *zOTU_1810 unclassified Prevotella* | AC | 9.46E-03 |  |  |
|  |  | *zOTU_233 Prevotella maculosa* | AC | 9.21E-03 |  |  |
|  |  | *zOTU_177 Selenomonas HOT 442/sputigena** | AC | 1.85E-03 |  |  |
|  |  | *zOTU_347 Selenomonas flueggei** | AC | 3.40E-02 |  |  |
|  |  | *zOTU_1076 unclassified Selenomonas* | AC | 3.15E-03 |  |  |
|  |  | *zOTU_398 unclassified Streptococcus* | AC | 2.51E-02 |  |  |
|  |  | *zOTU_208 Selenomonas dianae* | AC | 2.33E-03 |  |  |
|  |  | *zOTU_2076 Selenomonas infelix/HOT 126/HOT 138/HOT 146/HOT 479/HOT 481/HOT 892/HOT 919/HOT 936* | AC | 1.29E-02 |  |  |
|  |  | *zOTU_814 unclassified Veillonella* | AC | 4.07E-02 |  |  |
|  |  | *zOTU_459 Centipeda periodontii** | AC | 3.32E-03 |  |  |
|  |  | *zOTU_110 Prevotella melaninogenica/scopos/HOT 314** | AC | 9.80E-04 |  |  |
|  |  | *zOTU_76 Prevotella HOT 317* | AC | 6.09E-04 |  |  |
|  |  | *zOTU_2302 Lachnospiraceae [G-3] HOT 100* | CFAT | 3.10E-02 |  |  |
|  |  | *zOTU_301 Lachnospiraceae [G-3] HOT 100* | CFAT | 3.19E-02 |  |  |
|  | 13 | *zOTU_23 Prevotella histicola** | AC | 2.67E-05 | 4.78E-02 |  |
|  | 14 | *zOTU_15 Veillonella dispar* | AC | 3.03E-06 | 3.53E-02 |  |
|  | 15 | *zOTU_82 Veillonella denticariosi/parvula** | AC | 4.68E-05 | 3.53E-02 |  |

*P*-value represents the results from global test.

* indicates zOTUs additionally blasted on HOMD website with similarity ≥ 98.5%.

| **E** | **dental plaque samples in the CFAT vs AC group at T3** | | | | | |
| --- | --- | --- | --- | --- | --- | --- |
|  | ***n* of zOTU groups** | **Numbers and taxonomical names of the zOTUs at genus and species level** | **Associated group** | ***P*-value of the individual zOTU** | ***P*-value of the group of zOTUs corrected for multiple testing** | **Bonferroni- corrected Overall *P*-value** |
|  | 1 | *zOTU_93 Leptotrichia wadei* | AC | 1.16E-04 | 4.17E-02 | 2.30E-03 |
|  | 2 | *zOTU_411 unclassified Veillonella* | AC | 1.34E-04 | 4.55E-02 |  |
|  |  | *zOTU_456 unclassified Veillonella* | AC | 4.16E-03 |  |  |
|  |  | *zOTU_514 Veillonella atypica/dispar** | AC | 7.08E-03 |  |  |
|  |  | *zOTU_1816 unclassified Veillonella* | AC | 5.03E-03 |  |  |
|  |  | *zOTU_309 Streptococcus dentisani/infantis/mitis/oralis/HOT 058/HOT 061/HOT 064/HOT 070/HOT 423/HOT 431/tigurinus* | AC | 7.10E-03 |  |  |
|  |  | *zOTU_828 unclassified Veillonella* | AC | 1.34E-03 |  |  |
|  |  | *zOTU_1422 Veillonella dispar* | AC | 2.33E-02 |  |  |
|  |  | *zOTU_1490 Streptococcus dentisani/infantis/mitis/oralis/HOT 058/HOT 061/HOT 064/HOT 070/HOT 423/HOT 431/tigurinus* | AC | 3.54E-03 |  |  |
|  |  | *zOTU_15 Veillonella dispar* | AC | 3.62E-03 |  |  |
|  |  | *zOTU_1179 unclassified Veillonella* | AC | 6.60E-03 |  |  |
|  |  | *zOTU_490 Veillonella dispar* | AC | 1.87E-03 |  |  |
|  |  | *zOTU_591 unclassified Actinomyces* | AC | 3.41E-03 |  |  |
|  |  | *zOTU_744 unclassified Actinomyces* | AC | 1.56E-02 |  |  |
|  |  | *zOTU_708 Veillonella dispar* | AC | 4.08E-02 |  |  |
|  |  | *zOTU_628 Veillonella dispar* | AC | 6.24E-03 |  |  |
|  | 3 | *zOTU_24 Leptotrichia hongkongensis* | AC | 1.45E-04 | 2.63E-02 |  |
|  | 4 | *zOTU_108 Porphyromonas catoniae/HOT 275/HOT 277/HOT 284* | CFAT | 1.54E-04 | 4.91E-02 |  |
|  | 5 | *zOTU_73 Aggregatibacter aphrophilus/paraphrophilus* | CFAT | 6.92E-04 | 4.71E-02 |  |
|  |  | *zOTU_2025 unclassified Neisseria* | CFAT | 1.18E-03 |  |  |
|  |  | *zOTU_978 unclassified Aggregatibacter* | CFAT | 2.49E-03 |  |  |
|  |  | *zOTU_1080 unclassified Aggregatibacter* | CFAT | 1.32E-03 |  |  |
|  |  | *zOTU_1599 unclassified Aggregatibacter* | CFAT | 1.81E-03 |  |  |
|  |  | *zOTU_1790 unclassified Aggregatibacter* | CFAT | 7.20E-03 |  |  |
|  |  | *zOTU_205 unclassified Haemophilus** | CFAT | 1.13E-02 |  |  |
|  |  | *zOTU_242 unclassified Aggregatibacter** | CFAT | 4.60E-02 |  |  |
|  |  | *zOTU_67 Aggregatibacter HOT 513/HOT 898** | CFAT | 2.78E-02 |  |  |

*P*-value represents the results from global test.

* indicates zOTUs additionally blasted on HOMD website with similarity ≥ 98.5%.

| **F** | **dental plaque samples in the C6.5 vs EC group at T3** | | | | | |
| --- | --- | --- | --- | --- | --- | --- |
|  | ***n* of zOTU groups** | **Numbers and taxonomical names of the zOTUs at genus and species level** | **Associated group** | ***P*-value of the individual zOTU** | ***P*-value of the group of zOTUs corrected for multiple testing** | **Bonferroni- corrected Overall *P*-value** |
|  | 1 | *zOTU_334 Lachnospiraceae[G-3] HOT 100* | C6.5 | 4.31E-06 | 3.81E-02 | 2.80E-03 |
|  | 2 | *zOTU_2175 unclassified Selenomonas* | C6.5 | 2.83E-04 | 2.87E-02 |  |
|  |  | *zOTU_76 Prevotella HOT 317* | C6.5 | 7.16E-04 |  |  |
|  |  | *zOTU_2278 Selenomonas HOT 136/HOT 149/HOT 478* | C6.5 | 1.05E-03 |  |  |
|  |  | *zOTU_2331 unclassified Selenomonas* | C6.5 | 1.04E-02 |  |  |
|  |  | *zOTU_2076 Selenomonas infelix/HOT 126/HOT 138/HOT 146/HOT 479/HOT 481/HOT 892/HOT 919/HOT 936* | C6.5 | 1.03E-02 |  |  |
|  |  | *zOTU_1076 unclassified Selenomonas* | C6.5 | 1.50E-03 |  |  |
|  |  | *zOTU_398 unclassified Streptococcus* | C6.5 | 1.74E-03 |  |  |
|  |  | *zOTU_1464 unclassified Selenomonas* | C6.5 | 4.11E-03 |  |  |
|  |  | *zOTU_177 Selenomonas HOT 442/sputigena** | C6.5 | 3.75E-03 |  |  |
|  |  | *zOTU_347 Selenomonas flueggei** | C6.5 | 9.38E-03 |  |  |
|  |  | *zOTU_153 unclassified Selenomonas* | C6.5 | 3.92E-02 |  |  |
|  |  | *zOTU_990 unclassified Selenomonas* | C6.5 | 1.54E-02 |  |  |
|  |  | *zOTU_1236 unclassified Selenomonas* | C6.5 | 2.19E-02 |  |  |
|  |  | *zOTU_2143 unclassified Selenomonas* | C6.5 | 3.54E-02 |  |  |
|  |  | *zOTU_150 Selenomonas noxia** | C6.5 | 1.09E-02 |  |  |
|  |  | *zOTU_161 Selenomonas artemidis/HOT 137** | C6.5 | 6.09E-04 |  |  |
|  |  | *zOTU_1832 Streptococcus sanguinis* | C6.5 | 6.16E-03 |  |  |
|  | 3 | *zOTU_102 Fusobacterium nucleatum subsp animalis* | C6.5 | 2.96E-04 | 2.71E-02 |  |
|  |  | *zOTU_159 Capnocytophaga HOT 326* | C6.5 | 1.58E-02 |  |  |
|  |  | *zOTU_113 Fusobacterium nucleatum subsp animalis* | C6.5 | 1.61E-03 |  |  |
|  |  | *zOTU_164 Campylobacter gracilis* | C6.5 | 2.79E-02 |  |  |
|  |  | *zOTU_352 Actinomyces HOT 525* | C6.5 | 2.37E-02 |  |  |
|  | 4 | *zOTU_1163 unclassified Fusobacterium* | C6.5 | 1.36E-04 | 4.55E-02 |  |
|  |  | *zOTU_37 Fusobacterium nucleatum subsp nucleatum/HOT 203** | C6.5 | 6.87E-04 |  |  |
|  |  | *zOTU_923 Fusobacterium nucleatum subsp nucleatum/HOT 203** | C6.5 | 1.36E-03 |  |  |
|  |  | *zOTU_95 Lachnoanaerobaculum umeaense* | C6.5 | 1.72E-02 |  |  |
|  |  | *zOTU_1463 Fusobacterium periodonticum* | C6.5 | 2.57E-03 |  |  |
|  | 5 | *zOTU_270 Capnocytophaga HOT 324** | C6.5 | 1.46E-04 | 4.32E-02 |  |
|  |  | *zOTU_1926 unclassified Capnocytophaga* | C6.5 | 2.37E-03 |  |  |
|  | 6 | *zOTU_457 unclassified Propionibacterium* | C6.5 | 5.40E-04 | 3.38E-02 |  |
|  |  | *zOTU_521 unclassified Propionibacterium* | C6.5 | 7.06E-04 |  |  |
|  |  | *zOTU_214 unclassified Propionibacterium* | C6.5 | 1.64E-03 |  |  |
|  |  | *zOTU_432 unclassified Propionibacterium* | C6.5 | 2.12E-02 |  |  |
|  |  | *zOTU_509 unclassified Propionibacterium* | C6.5 | 2.71E-02 |  |  |
|  |  | *zOTU_855 unclassified Propionibacterium* | C6.5 | 2.53E-03 |  |  |
|  |  | *zOTU_1598 unclassified Propionibacterium* | C6.5 | 3.23E-03 |  |  |
|  |  | *zOTU_883 unclassified Propionibacterium* | C6.5 | 4.50E-02 |  |  |
|  |  | *zOTU_942 Neisseria oralis* | C6.5 | 4.14E-03 |  |  |
|  |  | *zOTU_2394 Neisseria oralis* | C6.5 | 3.24E-02 |  |  |
|  |  | *zOTU_104 Neisseria oralis* | C6.5 | 1.26E-02 |  |  |
|  |  | *zOTU_2220 unclassified Neisseria* | C6.5 | 2.95E-02 |  |  |

*P*-value represents the results from global test.

* indicates zOTUs additionally blasted on HOMD website with similarity ≥ 98.5%.

**Appendix Table 4:** zOTUs discriminating the microbiome composition according to the caries groups of the children in the saliva at T1 **(A)** and the dental plaque samples at T2 **(BC)**.

| **A** | **Groups compared at T1 in the salivary samples** | |
| --- | --- | --- |
|  | **CFAT** | **C4** |
|  | *zOTU_3 Haemophilus parainfluenzae* |  |
|  | *zOTU_892 Haemophilus parainfluenzae* |  |
|  | *zOTU_1886 unclassified Leptotrichia* |  |
|  | *zOTU_1 Streptococcus dentisani/infantis/mitis/oralis/HOT 058/HOT 061/HOT 064/HOT 070/HOT 423/HOT 431/tigurinus* |  |
|  | *zOTU_1944 Streptococcus dentisani/infantis/mitis/oralis/HOT 058/HOT 061/HOT 064/HOT 070/HOT 423/HOT 431/tigurinus* |  |
|  | *zOTU_2174 Streptococcus dentisani/infantis/mitis/oralis/HOT 058/HOT 061/HOT 064/HOT 070/HOT 423/HOT 431/tigurinus* |  |

The results from ShrinkBayes analyses (BFDR ≤ 0.1).

zOTUs, which significantly contributed to the differences among the caries groups, shown in the columns under the name of the respective caries group.

zOTUs were at a higher proportion in the caries group they are written compared to the other group.

zOTUs are ordered by name of genera in alphabetical order

| **B** | **Groups compared at T2 in the dental plaque samples** | |
| --- | --- | --- |
|  | **CFAT** | **C4** |
|  | *zOTU_1560 unclassified Actinomyces* | *zOTU_591 unclassified Actinomyces* |
|  | *zOTU_20 Lautropia mirabilis* | *zOTU_1319 unclassified Actinomyces* |
|  | *zOTU_454 Lautropia mirabilis* | *zOTU_1230 unclassified Actinobacteria* |
|  | *zOTU_2239 Lautropia mirabilis* | *zOTU_439 Capnocytophaga HOT 336/HOT 864* |
|  | *zOTU_145 Moraxella catarrhalis* | *zOTU_1579 Corynebacterium durum* |
|  | *zOTU_1496 Neisseria flava/mucosa/pharyngis/sicca** | *zOTU_2262 Corynebacterium durum* |
|  | *zOTU_1109 unclassified Neisseria* | *zOTU_1062 Corynebacterium matruchotii* |
|  | *zOTU_1955 unclassified Neisseria* | *zOTU_1184 Corynebacterium matruchotii* |
|  | *zOTU_2220 unclassified Neisseria* | *zOTU_1655 Corynebacterium matruchotii* |
|  | *zOTU_1708 unclassified Proteobacteria* | *zOTU_1794 Corynebacterium matruchotii* |
|  |  | *zOTU_1881 Corynebacterium matruchotii* |
|  |  | *zOTU_2186 Corynebacterium matruchotii* |
|  |  | *zOTU_1138 unclassified Corynebacterium** |
|  |  | *zOTU_802 Granulicatella adiacens* |
|  |  | *zOTU_178 Leptotrichia hofstadii** |
|  |  | *zOTU_16 Rothia dentocariosa* |
|  |  | *zOTU_2209 Rothia dentocariosa* |
|  |  | *zOTU_26 Streptococcus cristatus/oligofermentans/sinensis** |
|  |  | *zOTU_34 Streptococcus gordonii/HOT 056** |
|  |  | *zOTU_47 Streptococcus gordonii/HOT 056** |
|  |  | *zOTU_1548 Streptococcus gordonii/HOT 056** |
|  |  | *zOTU_1205 unclassified Streptococcus* |
|  |  | *zOTU_1534 unclassified Streptococcus* |
|  |  | *zOTU_1622 unclassified Streptococcus* |
|  |  | *zOTU_2251 unclassified Streptococcus* |
|  |  | *zOTU_2360 unclassified Streptococcus* |
|  |  | *zOTU_869 Veillonella atypica/dispar** |
|  |  | *zOTU_490 Veillonella dispar* |
|  |  | *zOTU_1259 Veillonella dispar* |
|  |  | *zOTU_2335 Veillonella dispar* |
|  |  | *zOTU_1088 unclassified Veillonella* |
|  |  | *zOTU_1179 unclassified Veillonella* |
|  |  | *zOTU_2165 unclassified Veillonella* |
|  |  | *zOTU_2229 unclassified Veillonella* |

The results from ShrinkBayes analyses (BFDR ≤ 0.1).

zOTUs, which significantly contributed to the differences among the caries groups, shown in the columns under the name of the respective caries group.

zOTUs were at a higher proportion in the caries group they are written compared to the other group.

zOTUs are ordered by name of genera in alphabetical order

zOTUs are ordered by name of genera in alphabetical order

* indicates zOTUs additionally blasted on HOMD website with similarity ≥ 98.5%.

| **C** | **Groups compared at T2 in the dental plaque samples** | |
| --- | --- | --- |
|  | **CFAT** | **EC** |
|  | *zOTU_36 Abiotrophia defectiva* | *zOTU_1078 Leptotrichia hongkongensis* |
|  | *zOTU_74 Capnocytophaga gingivalis* | *zOTU_1301 Streptococcus dentisani/infantis/mitis/oralis/HOT 058/HOT 061/HOT 064/HOT 070/HOT 423/HOT 431/tigurinus* |
|  | *zOTU_2061 Fusobacterium nucleatum subsp polymorphum/HOT 203** | *zOTU_490 Veillonella dispar* |
|  | *zOTU_20 Lautropia mirabilis* |  |
|  | *zOTU_2239 Lautropia mirabilis* |  |
|  | *zOTU_2336 Streptococcus lactarius/peroris/HOT 074** |  |
|  | *zOTU_4 Streptococcus sanguinis* |  |
|  | *zOTU_1560 unclassified Actinomyces* |  |
|  | *zOTU_1364 unclassified Streptococcus* |  |
|  | *zOTU_1899 unclassified Streptococcus* |  |

The results from ShrinkBayes analyses (BFDR ≤ 0.1).

zOTUs, which significantly contributed to the differences among the caries groups, shown in the columns under the name of the respective caries group.

zOTUs were at a higher proportion in the caries group they are written compared to the other group.

zOTUs are ordered by name of genera in alphabetical order

zOTUs are ordered by name of genera in alphabetical order

* indicates zOTUs additionally blasted on HOMD website with similarity ≥ 98.5%.

**Appendix Table 5:** zOTUs discriminating the microbiome composition according to the caries groups of the children in the saliva samples at T3 **(ABC)**.

| **A** | **Groups compared at T3 in the salivary samples** | |
| --- | --- | --- |
|  | **CFAT** | **C6.5** |
|  |  | *zOTU_63 Alloprevotella HOT 308* |
|  |  | *zOTU_256 unclassified Aggregatibacter/Haemophilus** |
|  |  | *zOTU_2211 Bergeyella HOT 931* |
|  |  | *zOTU_89 Campylobacter concisus** |
|  |  | *zOTU_183 Campylobacter concisus* |
|  |  | *zOTU_364 Capnocytophaga HOT 335/HOT 336* |
|  |  | *zOTU_227 Catonella morbi* |
|  |  | *zOTU_98 unclassified Capnocytophaga* |
|  |  | *zOTU_22 unclassified Kingella/Neisseria** |
|  |  | *zOTU_536 unclassified Kingella/Neisseria** |
|  |  | *zOTU_1562 unclassified Fusobacteriales* |
|  |  | *zOTU_1657 unclassified Fusobacterium* |
|  |  | *zOTU_136 Lachnoanaerobaculum orale** |
|  |  | *zOTU_152 Lachnospiraceae [G-2] HOT 096* |
|  |  | *zOTU_1523 Leptotrichia buccalis/HOT 225/HOT 417/HOT 463** |
|  |  | *zOTU_1702 Leptotrichia buccalis/HOT 225/HOT 417/HOT 463** |
|  |  | *zOTU_1380 Leptotrichia HOT 225* |
|  |  | *zOTU_40 Leptotrichia HOT 417** |
|  |  | *zOTU_1073 Leptotrichia HOT 417** |
|  |  | *zOTU_1237 Leptotrichia HOT 417** |
|  |  | *zOTU_1660 Leptotrichia HOT 417** |
|  |  | *zOTU_1909 Leptotrichia HOT 417** |
|  |  | *zOTU_1115 unclassified Leptotrichia* |
|  |  | *zOTU_1429 unclassified Leptotrichia* |
|  |  | *zOTU_2048 unclassified Leptotrichia* |
|  |  | *zOTU_2094 unclassified Leptotrichia* |
|  |  | *zOTU_2098 unclassified Leptotrichia* |
|  |  | *zOTU_2325 unclassified Leptotrichia* |
|  |  | *zOTU_96 Megasphaera micronuciformis* |
|  |  | *zOTU_149 Peptostreptococcus stomatis* |
|  |  | *zOTU_23 Prevotella histicola** |
|  |  | *zOTU_107 Prevotella histicola** |
|  |  | *zOTU_99 Prevotella histicola/melaninogenica** |
|  |  | *zOTU_328 Prevotella HOT 309** |
|  |  | *zOTU_120 Prevotella HOT 313** |
|  |  | *zOTU_2067 Prevotella HOT 942* |
|  |  | *zOTU_1385 Prevotella maculosa* |
|  |  | *zOTU_475 Prevotella melaninogenica** |
|  |  | *zOTU_77 Prevotella nigrescens* |
|  |  | *zOTU_2371 Prevotella nigrescens* |
|  |  | *zOTU_151 Prevotella oulorum** |
|  |  | *zOTU_69 Prevotella pallens* |
|  |  | *zOTU_68 Prevotella salivae* |
|  |  | *zOTU_239 Prevotella salivae* |
|  |  | *zOTU_483 Prevotella salivae* |
|  |  | *zOTU_544 Prevotella salivae* |
|  |  | *zOTU_549 Prevotella salivae* |
|  |  | *zOTU_1820 Prevotella salivae* |
|  |  | *zOTU_109 Prevotella scopos/HOT 313/HOT 314** |
|  |  | *zOTU_39 Prevotella veroralis* |
|  |  | *zOTU_51 Prevotella veroralis* |
|  |  | *zOTU_193 Prevotella veroralis* |
|  |  | *zOTU_671 unclassified Prevotella* |
|  |  | *zOTU_709 unclassified Prevotella* |
|  |  | *zOTU_740 unclassified Prevotella* |
|  |  | *zOTU_1406 unclassified Prevotella* |
|  |  | *zOTU_1810 unclassified Prevotella* |
|  |  | *zOTU_1986 unclassified Prevotella* |
|  |  | *zOTU_54 Rothia mucilaginosa* |
|  |  | *zOTU_1229 Selenomonas HOT 442/sputigena** |
|  |  | *zOTU_201 Stomatobaculum longum** |
|  |  | *zOTU_362 Stomatobaculum longum** |
|  |  | *zOTU_217 Streptococcus anginosus* |
|  |  | *zOTU_1383 Streptococcus dentisani/infantis/mitis/oralis/HOT 058/HOT 061/HOT 064/HOT 070/HOT 423/HOT 431/tigurinus* |
|  |  | *zOTU_2085 Streptococcus dentisani/infantis/mitis/oralis/HOT 058/HOT 061/HOT 064/HOT 070/HOT 423/HOT 431/tigurinus* |
|  |  | *zOTU_34 Streptococcus gordonii/HOT 056** |
|  |  | *zOTU_973 Streptococcus salivarius/vestibularis* |
|  |  | *zOTU_1710 Streptococcus salivarius/vestibularis* |
|  |  | *zOTU_336 Tannerella HOT 286/HOT 808* |
|  |  | *zOTU_1475 Tannerella HOT 286/HOT 808* |
|  |  | *zOTU_346 Tannerella HOT 286/HOT 808/HOT 916** |
|  |  | *zOTU_1705 Veillonella atypica/dispar** |
|  |  | *zOTU_1201 Veillonella dispar* |
|  |  | *zOTU_1218 Veillonella parvula* |
|  |  | *zOTU_449 unclassified Veillonella* |
|  |  | *zOTU_814 unclassified Veillonella* |
|  |  | *zOTU_1179 unclassified Veillonella* |
|  |  | *zOTU_1398 unclassified Veillonella* |
|  |  | *zOTU_1403 unclassified Veillonella* |
|  |  | *zOTU_1575 unclassified Veillonella* |
|  |  | *zOTU_2091 unclassified Veillonella* |

The results from ShrinkBayes analyses (BFDR ≤ 0.1).

zOTUs, which significantly contributed to the differences among the caries groups, shown in the columns under the name of the respective caries group.

zOTUs were at a higher proportion in the caries group they are written compared to the other group.

zOTUs are ordered by name of genera in alphabetical order

zOTUs are ordered by name of genera in alphabetical order

* indicates zOTUs additionally blasted on HOMD website with similarity ≥ 98.5%.

| **B** | **Groups compared at T3 in the salivary samples** | |
| --- | --- | --- |
|  | **CFAT** | **EC** |
|  | *zOTU_78 Alloprevotella HOT 914** | *zOTU_2270 Actinomyces massiliensis* |
|  |  | *zOTU_183 Campylobacter concisus* |
|  |  | *zOTU_1237 Leptotrichia HOT 417** |
|  |  | *zOTU_23 Prevotella histicola** |
|  |  | *zOTU_151 Prevotella oulorum** |
|  |  | *zOTU_201 Stomatobaculum longum** |
|  |  | *zOTU_34 Streptococcus gordonii/HOT 056** |
|  |  | *zOTU_1339 unclassified Leptotrichia* |
|  |  | *zOTU_1429 unclassified Leptotrichia* |

The results from ShrinkBayes analyses (BFDR ≤ 0.1).

zOTUs, which significantly contributed to the differences among the caries groups, shown in the columns under the name of the respective caries group.

zOTUs were at a higher proportion in the caries group they are written compared to the other group.

zOTUs are ordered by name of genera in alphabetical order

zOTUs are ordered by name of genera in alphabetical order

* indicates zOTUs additionally blasted on HOMD website with similarity ≥ 98.5%.

| **C** | **Groups compared at T3 in the salivary samples** | |
| --- | --- | --- |
|  | **CFAT** | **AC** |
|  | *zOTU_87 Haemophilus pittmaniae** | *zOTU_137 Actinobaculum HOT 183* |
|  | *zOTU_257 Leptotrichia HOT 212/HOT 215/HOT 217** | *zOTU_49 Aggregatibacter segnis/HOT 458/HOT 512* |
|  | *zOTU_2274 unclassified Leptotrichia* | *zOTU_306 Alloprevotella HOT 912* |
|  | *zOTU_1231 Neisseria flavescens/subflava* | *zOTU_256 unclassified Aggregatibacter/Haemophilus** |
|  | *zOTU_2371 Prevotella nigrescens* | *zOTU_340 unclassified Aggregatibacter/Haemophilus** |
|  | *zOTU_124 Rothia mucilaginosa* | *zOTU_183 Campylobacter concisus* |
|  | *zOTU_262 SR1 [G-1] HOT 875** | *zOTU_226 Campylobacter rectus/showae* |
|  | *zOTU_1204 Streptococcus lactarius/ peroris/HOT 074** | *zOTU_57 Capnocytophaga granulosa** |
|  | *zOTU_666 unclassified Veillonella* | *zOTU_98 unclassified Capnocytophaga* |
|  | *zOTU_64 Veillonella rogosae** | *zOTU_212 unclassified Capnocytophaga* |
|  |  | *zOTU_459 Centipeda periodontii** |
|  |  | *zOTU_2186 Corynebacterium matruchotii* |
|  |  | *zOTU_1184 Corynebacterium matruchotii* |
|  |  | *zOTU_113 Fusobacterium nucleatum subsp. animalis* |
|  |  | *zOTU_1562 unclassified Fusobacteriales* |
|  |  | *zOTU_44 Kingella oralis* |
|  |  | *zOTU_1576 unclassified Kingella* |
|  |  | *zOTU_136 Lachnoanaerobaculum orale** |
|  |  | *zOTU_174 Lachnoanaerobaculum saburreum* |
|  |  | *zOTU_84 Leptotrichia buccalis** |
|  |  | *zOTU_2227 Leptotrichia buccalis** |
|  |  | *zOTU_1651 Leptotrichia buccalis/HOT 225** |
|  |  | *zOTU_1523 Leptotrichia buccalis/HOT 225/HOT 417/HOT 463** |
|  |  | *zOTU_311 Leptotrichia hofstadii** |
|  |  | *zOTU_24 Leptotrichia hongkongensis* |
|  |  | *zOTU_1042 Leptotrichia hongkongensis* |
|  |  | *zOTU_2260 Leptotrichia hongkongensis** |
|  |  | *zOTU_1270 Leptotrichia HOT 225* |
|  |  | *zOTU_1266 Leptotrichia HOT 225* |
|  |  | *zOTU_1380 Leptotrichia HOT 225* |
|  |  | *zOTU_40 Leptotrichia HOT 417** |
|  |  | *zOTU_1237 Leptotrichia HOT 417** |
|  |  | *zOTU_1660 Leptotrichia HOT 417** |
|  |  | *zOTU_1073 Leptotrichia HOT 417** |
|  |  | *zOTU_2228 Leptotrichia HOT 417** |
|  |  | *zOTU_1909 Leptotrichia HOT 417** |
|  |  | *zOTU_399 Leptotrichia HOT 498* |
|  |  | *zOTU_143 Leptotrichia HOT 498* |
|  |  | *zOTU_1680 Leptotrichia HOT 498* |
|  |  | *zOTU_93 Leptotrichia wadei* |
|  |  | *zOTU_805 unclassified Leptotrichia* |
|  |  | *zOTU_1115 unclassified Leptotrichia* |
|  |  | *zOTU_1339 unclassified Leptotrichia* |
|  |  | *zOTU_1429 unclassified Leptotrichia* |
|  |  | *zOTU_1489 unclassified Leptotrichia* |
|  |  | *zOTU_1508 unclassified Leptotrichia* |
|  |  | *zOTU_1776 unclassified Leptotrichia* |
|  |  | *zOTU_2098 unclassified Leptotrichia* |
|  |  | *zOTU_2321 unclassified Leptotrichia* |
|  |  | *zOTU_2391 unclassified Leptotrichia* |
|  |  | *zOTU_96 Megasphaera micronuciformis* |
|  |  | *zOTU_1288 Neisseria flava/ flavescens/ mucosa/ pharyngis/ sicca/ subflava** |
|  |  | *zOTU_126 Neisseria HOT 018** |
|  |  | *zOTU_1847 unclassified Neisseria* |
|  |  | *zOTU_1993 unclassified Neisseria* |
|  |  | *zOTU_809 Porphyromonas pasteri/HOT 278* |
|  |  | *zOTU_147 unclassified Porphyromonas* |
|  |  | *zOTU_157 Prevotella denticola* |
|  |  | *zOTU_23 Prevotella histicola** |
|  |  | *zOTU_1775 Prevotella HOT 317* |
|  |  | *zOTU_475 Prevotella melaninogenica** |
|  |  | *zOTU_273 Prevotella micans* |
|  |  | *zOTU_77 Prevotella nigrescens* |
|  |  | *zOTU_215 Prevotella oris* |
|  |  | *zOTU_177 Prevotella oulorum** |
|  |  | *zOTU_68 Prevotella salivae* |
|  |  | *zOTU_39 Prevotella veroralis* |
|  |  | *zOTU_193 Prevotella veroralis* |
|  |  | *zOTU_151 unclassified Prevotella* |
|  |  | *zOTU_671 unclassified Prevotella* |
|  |  | *zOTU_709 unclassified Prevotella* |
|  |  | *zOTU_1810 unclassified Prevotella* |
|  |  | *zOTU_16 Rothia dentocariosa* |
|  |  | *zOTU_2268 Rothia dentocariosa* |
|  |  | *zOTU_208 Selenomonas dianae* |
|  |  | *zOTU_2076 Selenomonas infelix/HOT 126/HOT 138/HOT 146/HOT 479/HOT 481/HOT 892/HOT 919/HOT 936* |
|  |  | *zOTU_990 unclassified Selenomonas* |
|  |  | *zOTU_1076 unclassified Selenomonas* |
|  |  | *zOTU_201 Stomatobaculum longum** |
|  |  | *zOTU_217 Streptococcus anginosus* |
|  |  | *zOTU_47 Streptococcus gordonii/HOT 056** |
|  |  | *zOTU_34 Streptococcus gordonii/HOT 056** |
|  |  | *zOTU_55 Streptococcus mutans* |
|  |  | *zOTU_1710 Streptococcus salivarius/vestibularis* |
|  |  | *zOTU_1397 Streptococcus salivarius/vestibularis* |
|  |  | *zOTU_346 Tannerella HOT 286/HOT 808/HOT 916** |
|  |  | *zOTU_82 Veillonella denticariosi/ parvula** |
|  |  | *zOTU_514 Veillonella atypica/dispar** |
|  |  | *zOTU_15 Veillonella dispar* |
|  |  | *zOTU_915 Veillonella dispar* |
|  |  | *zOTU_1914 Veillonella dispar* |
|  |  | *zOTU_411 unclassified Veillonella* |
|  |  | *zOTU_814 unclassified Veillonella* |
|  |  | *zOTU_1179 unclassified Veillonella* |
|  |  | *zOTU_1608 unclassified Veillonella* |
|  |  | *zOTU_2091 unclassified Veillonella* |

The results from ShrinkBayes analyses (BFDR ≤ 0.1).

zOTUs, which significantly contributed to the differences among the caries groups, shown in the columns under the name of the respective caries group.

zOTUs were at a higher proportion in the caries group they are written compared to the other group.

zOTUs are ordered by name of genera in alphabetical order

zOTUs are ordered by name of genera in alphabetical order

* indicates zOTUs additionally blasted on HOMD website with similarity ≥ 98.5%.

**Appendix Table 6:** zOTUs discriminating the microbiome composition according to the caries groups of the children in the dental plaque samples at T3 **(ABC)**.

| **A** | **Groups compared at T3 in the dental plaque samples** | |
| --- | --- | --- |
|  | **CFAT** | **C6.5** |
|  | *zOTU_73 Aggregatibacter aphrophilus/paraphrophilus* | *zOTU_287 Actinomyces HOT 414* |
|  | *zOTU_296 Cardiobacterium valvarum* | *zOTU_317 Actinomyces HOT 448* |
|  | *zOTU_35 Haemophilus parahaemolyticus/ paraphrohaemolyticus/ sputorum** | *zOTU_352 Actinomyces HOT 525* |
|  | *zOTU_1661 Rothia aeria* | *zOTU_2275 Actinomyces naeslundii/ oris/HOT 169/HOT 171/HOT 175** |
|  | *zOTU_12 Streptococcus australis/parasanguinis_I/parasanguinis_II/HOT 057/HOT 066* | *zOTU_1936 unclassified Actinomyces* |
|  | *zOTU_2336 Streptococcus lactarius/ peroris/HOT 074** | *zOTU_183 Campylobacter concisus* |
|  |  | *zOTU_195 Campylobacter gracilis* |
|  |  | *zOTU_270 Capnocytophaga HOT 324** |
|  |  | *zOTU_364 Capnocytophaga HOT 335/HOT 336* |
|  |  | *zOTU_439 Capnocytophaga HOT 336/HOT 864* |
|  |  | *zOTU_513 Capnocytophaga HOT 338* |
|  |  | *zOTU_409 Corynebacterium diphtheriae* |
|  |  | *zOTU_290 Dialister invisus* |
|  |  | *zOTU_113 Fusobacterium nucleatum subsp. animalis* |
|  |  | *zOTU_1657 unclassified Fusobacterium* |
|  |  | *zOTU_1711 unclassified Fusobacterium* |
|  |  | *zOTU_1562 unclassified Fusobacteriales* |
|  |  | *zOTU_2238 unclassified Fusobacteriales* |
|  |  | *zOTU_1125 Leptotrichia hongkongensis* |
|  |  | *zOTU_1471 Leptotrichia hongkongensis* |
|  |  | *zOTU_1908 Leptotrichia hongkongensis* |
|  |  | *zOTU_210 Leptotrichia HOT 212* |
|  |  | *zOTU_1380 Leptotrichia HOT 225* |
|  |  | *zOTU_40 Leptotrichia HOT 417** |
|  |  | *zOTU_1073 Leptotrichia HOT 417** |
|  |  | *zOTU_1237 Leptotrichia HOT 417** |
|  |  | *zOTU_1660 Leptotrichia HOT 417** |
|  |  | *zOTU_2228 Leptotrichia HOT 417** |
|  |  | *zOTU_258 Leptotrichia HOT 498* |
|  |  | *zOTU_93 Leptotrichia wadei* |
|  |  | *zOTU_845 unclassified Leptotrichia* |
|  |  | *zOTU_848 unclassified Leptotrichia* |
|  |  | *zOTU_926 unclassified Leptotrichia* |
|  |  | *zOTU_951 unclassified Leptotrichia* |
|  |  | *zOTU_974 unclassified Leptotrichia* |
|  |  | *zOTU_1023 unclassified Leptotrichia* |
|  |  | *zOTU_1053 unclassified Leptotrichia* |
|  |  | *zOTU_1115 unclassified Leptotrichia* |
|  |  | *zOTU_1245 unclassified Leptotrichia* |
|  |  | *zOTU_1293 unclassified Leptotrichia* |
|  |  | *zOTU_1429 unclassified Leptotrichia* |
|  |  | *zOTU_1508 unclassified Leptotrichia* |
|  |  | *zOTU_1515 unclassified Leptotrichia* |
|  |  | *zOTU_1530 unclassified Leptotrichia* |
|  |  | *zOTU_1753 unclassified Leptotrichia* |
|  |  | *zOTU_2048 unclassified Leptotrichia* |
|  |  | *zOTU_2098 unclassified Leptotrichia* |
|  |  | *zOTU_2308 unclassified Leptotrichia* |
|  |  | *zOTU_2325 unclassified Leptotrichia* |
|  |  | *zOTU_2398 unclassified Leptotrichia* |
|  |  | *zOTU_363 Oribacterium HOT 078* |
|  |  | *zOTU_705 Porphyromonas pasteri/HOT 278* |
|  |  | *zOTU_909 Porphyromonas pasteri/HOT 278* |
|  |  | *zOTU_23 Prevotella histicola** |
|  |  | *zOTU_107 Prevotella histicola** |
|  |  | *zOTU_176 Prevotella histicola/HOT 306/HOT 313** |
|  |  | *zOTU_1154 Prevotella HOT 317* |
|  |  | *zOTU_1187 Prevotella HOT 317* |
|  |  | *zOTU_1563 Prevotella HOT 317* |
|  |  | *zOTU_1775 Prevotella HOT 317* |
|  |  | *zOTU_233 Prevotella maculosa* |
|  |  | *zOTU_1140 Prevotella melaninogenica* |
|  |  | *zOTU_77 Prevotella nigrescens* |
|  |  | *zOTU_163 Prevotella oris* |
|  |  | *zOTU_215 Prevotella oris* |
|  |  | *zOTU_243 Prevotella oulorum* |
|  |  | *zOTU_68 Prevotella salivae* |
|  |  | *zOTU_109 Prevotella scopos/HOT 313/HOT 314** |
|  |  | *zOTU_397 unclassified Prevotella* |
|  |  | *zOTU_709 unclassified Prevotella* |
|  |  | *zOTU_1048 unclassified Prevotella* |
|  |  | *zOTU_1101 unclassified Prevotella* |
|  |  | *zOTU_1726 unclassified Prevotella* |
|  |  | *zOTU_1818 unclassified Prevotella* |
|  |  | *zOTU_1865 unclassified Prevotella* |
|  |  | *zOTU_2148 unclassified Prevotella* |
|  |  | *zOTU_855 unclassified Propionibacterium* |
|  |  | *zOTU_984 unclassified Propionibacterium* |
|  |  | *zOTU_169 Selenomonas HOT 136/HOT 149/HOT 478* |
|  |  | *zOTU_2278 Selenomonas HOT 136/HOT 149/HOT 478* |
|  |  | *zOTU_177 Selenomonas HOT 442/sputigena** |
|  |  | *zOTU_1229 Selenomonas HOT 442/sputigena** |
|  |  | *zOTU_299 Selenomonas infelix/HOT 126/HOT 138/HOT 146/HOT 479/HOT 481/HOT 892/HOT 919/HOT 936** |
|  |  | *zOTU_2039 Selenomonas infelix/HOT 126/HOT 138/HOT 146/HOT 479/HOT 481/HOT 892/HOT 919/HOT 936* |
|  |  | *zOTU_201 Stomatobaculum longum** |
|  |  | *zOTU_1474 Streptococcus dentisani/infantis/mitis/oralis/HOT 058/HOT 061/HOT 064/HOT 070/HOT 423/HOT 431/tigurinus* |
|  |  | *zOTU_1969 Streptococcus intermedius* |
|  |  | *zOTU_1803 Streptococcus pneumoniae** |
|  |  | *zOTU_1710 Streptococcus salivarius/vestibularis* |
|  |  | *zOTU_1205 unclassified Streptococcus* |
|  |  | *zOTU_1687 unclassified Selenomonas* |
|  |  | *zOTU_2341 Tannerella HOT 286/HOT 808* |
|  |  | *zOTU_1575 unclassified Veillonella* |
|  |  | *zOTU_1638 unclassified Veillonella* |
|  |  | *zOTU_1878 unclassified Veillonella* |
|  | **Groups compared at T3 in the dental plaque samples** | |
|  | **C6.5** | **EC** |
|  | *zOTU_2270 Actinomyces massiliensis* |  |
|  | *zOTU_2275 Actinomyces naeslundii/oris/HOT 169/HOT 171/HOT 175** |  |
|  | *zOTU_2339 unclassified Actinomyces* |  |
|  | *zOTU_225 Bergeyella HOT 931* |  |
|  | *zOTU_138 Campylobacter rectus/showae* |  |
|  | *zOTU_812 Capnocytophaga leadbetteri/HOT 324/HOT 335/HOT 336** |  |
|  | *zOTU_1184 Corynebacterium matruchotii* |  |
|  | *zOTU_332 Eikenella corrodens* |  |
|  | *zOTU_113 Fusobacterium nucleatum subsp. animalis* |  |
|  | *zOTU_70 Fusobacterium HOT 370** |  |
|  | *zOTU_95 Lachnoanaerobaculum umeaense* |  |
|  | *zOTU_334 Lachnospiraceae [G-3] HOT 100* |  |
|  | *zOTU_2403 Leptotrichia HOT 212* |  |
|  | *zOTU_1227 Leptotrichia HOT 225* |  |
|  | *zOTU_1073 Leptotrichia HOT 417** |  |
|  | *zOTU_848 unclassified Leptotrichia* |  |
|  | *zOTU_944 unclassified Leptotrichia* |  |
|  | *zOTU_1643 unclassified Leptotrichia* |  |
|  | *zOTU_1898 unclassified Leptotrichia* |  |
|  | *zOTU_2092 unclassified Leptotrichia* |  |
|  | *zOTU_2308 unclassified Leptotrichia* |  |
|  | *zOTU_2398 unclassified Leptotrichia* |  |
|  | *zOTU_942 Neisseria oralis* |  |
|  | *zOTU_2220 unclassified Neisseria* |  |
|  | *zOTU_1417 Prevotella oris* |  |
|  | *zOTU_1154 Prevotella HOT 317* |  |
|  | *zOTU_1860 Prevotella HOT 317* |  |
|  | *zOTU_1726 unclassified Prevotella* |  |
|  | *zOTU_1818 unclassified Prevotella* |  |
|  | *zOTU_213 Prevotella shahii/HOT 317** |  |
|  | *zOTU_214 unclassified Propionibacterium* |  |
|  | *zOTU_432 unclassified Propionibacterium* |  |
|  | *zOTU_457 unclassified Propionibacterium* |  |
|  | *zOTU_509 unclassified Propionibacterium* |  |
|  | *zOTU_521 unclassified Propionibacterium* |  |
|  | *zOTU_1598 unclassified Propionibacterium* |  |
|  | *zOTU_41 Rothia mucilaginosa* |  |
|  | *zOTU_2175 Selenomonas infelix/HOT 126/HOT 138/HOT 146/HOT 479/HOT 481/HOT 892/HOT 919/HOT 936* |  |
|  | *zOTU_1474 Streptococcus dentisani/infantis/mitis/oralis/HOT 058/HOT 061/HOT 064/HOT 070/HOT 423/HOT 431/tigurinus* |  |
|  | *zOTU_1786 Streptococcus dentisani/infantis/mitis/oralis/HOT 058/HOT 061/HOT 064/HOT 070/HOT 423/HOT 431/tigurinus* |  |
|  | *zOTU_613 Streptococcus dentisani/infantis/mitis/oralis/HOT 058/HOT 061/HOT 064/HOT 070/HOT 423/HOT 431/tigurinus* |  |
|  | *zOTU_694 Streptococcus salivarius/vestibularis* |  |
|  | *zOTU_1703 unclassified Firmicutes* |  |

The results from ShrinkBayes analyses (BFDR ≤ 0.1).

zOTUs, which significantly contributed to the differences among the caries groups, shown in the columns under the name of the respective caries group.

zOTUs were at a higher proportion in the caries group they are written compared to the other group.

zOTUs are ordered by name of genera in alphabetical order

zOTUs are ordered by name of genera in alphabetical order

* indicates zOTUs additionally blasted on HOMD website with similarity ≥ 98.5%.

| **B** | **Groups compared at T3 in the dental plaque samples** | |
| --- | --- | --- |
|  | **C6.5** | **AC** |
|  | *zOTU_2307 Aggregatibacter segnis/HOT 458/HOT 512* | *zOTU_33 Granulicatella adiacens* |
|  | *zOTU_1359 unclassified Actinomyces* | *zOTU_3 Haemophilus parainfluenzae* |
|  | *zOTU_138 Campylobacter rectus/showae* | *zOTU_663 Haemophilus parainfluenzae* |
|  | *zOTU_160 Capnocytophaga HOT 332* | *zOTU_1326 Haemophilus parainfluenzae* |
|  | *zOTU_45 Capnocytophaga leadbetteri* | *zOTU_44 Kingella oralis* |
|  | *zOTU_966 Capnocytophaga leadbetteri** | *zOTU_2129 Kingella oralis* |
|  | *zOTU_409 Corynebacterium diphtheriae* | *zOTU_943 Leptotrichia hongkongensis* |
|  | *zOTU_17 Corynebacterium matruchotii* | *zOTU_2389 Neisseria flava/flavescens/mucosa/pharyngis/sicca/subflava** |
|  | *zOTU_765 Corynebacterium matruchotii* | *zOTU_1455 Neisseria flava/mucosa/pharyngis/sicca** |
|  | *zOTU_1184 Corynebacterium matruchotii* | *zOTU_12 Streptococcus australis/parasanguinis I/parasanguinis II/HOT 057/HOT 066* |
|  | *zOTU_87 Haemophilus pittmaniae** | *zOTU_1944 Streptococcus dentisani/infantis/mitis/oralis/HOT 058/HOT 061/HOT 064/HOT 070/HOT 423/HOT 431/tigurinus* |
|  | *zOTU_132 Kingella HOT 012* | *zOTU_411 unclassified Veillonella* |
|  | *zOTU_22 unclassified Kingella/Neisseria** |  |
|  | *zOTU_265 unclassified Kingella/Neisseria** |  |
|  | *zOTU_1798 unclassified Kingella/Neisseria** |  |
|  | *zOTU_95 Lachnoanaerobaculum umeaense* |  |
|  | *zOTU_334 Lachnospiraceae [G-3] HOT 100* |  |
|  | *zOTU_103 Leptotrichia hofstadii** |  |
|  | *zOTU_66 Leptotrichia HOT 212* |  |
|  | *zOTU_1435 Leptotrichia HOT 212* |  |
|  | *zOTU_1831 Leptotrichia HOT 212* |  |
|  | *zOTU_1972 Leptotrichia HOT 212* |  |
|  | *zOTU_2081 Leptotrichia HOT 212* |  |
|  | *zOTU_2403 Leptotrichia HOT 212* |  |
|  | *zOTU_166 Leptotrichia HOT 225* |  |
|  | *zOTU_1447 Leptotrichia HOT 225* |  |
|  | *zOTU_2088 Leptotrichia HOT 909* |  |
|  | *zOTU_1842 Leptotrichia shahii* |  |
|  | *zOTU_676 unclassified Leptotrichia* |  |
|  | *zOTU_944 unclassified Leptotrichia* |  |
|  | *zOTU_2092 unclassified Leptotrichia* |  |
|  | *zOTU_2308 unclassified Leptotrichia* |  |
|  | *zOTU_1445 Neisseria flava/mucosa/pharyngis/sicca** |  |
|  | *zOTU_942 Neisseria oralis* |  |
|  | *zOTU_1092 unclassified Neisseria* |  |
|  | *zOTU_1187 Prevotella HOT 317* |  |
|  | *zOTU_1726 unclassified Prevotella* |  |
|  | *zOTU_1052 unclassified Proteobacteria* |  |
|  | *zOTU_2167 Rothia dentocariosa* |  |
|  | *zOTU_2076 Selenomonas infelix/HOT 126/HOT 138/HOT 146/HOT 479/HOT 481/HOT 892/HOT 919/HOT 936* |  |
|  | *zOTU_2175 Selenomonas infelix/HOT 126/HOT 138/HOT 146/HOT 479/HOT 481/HOT 892/HOT 919/HOT 936* |  |
|  | *zOTU_150 Selenomonas noxia** |  |
|  | *zOTU_1464 unclassified Selenomonas* |  |
|  | *zOTU_90 unclassified Sneathia* |  |
|  | *zOTU_613 Streptococcus dentisani/infantis/mitis/oralis/HOT 058/HOT 061/HOT 064/HOT 070/HOT 423/HOT 431/tigurinus* |  |
|  | *zOTU_55 Streptococcus mutans* |  |
|  | *zOTU_2281 unclassified Streptococcus* |  |
|  | **Groups compared at T3 in the dental plaque samples** | |
|  | **CFAT** | **EC** |
|  | *zOTU_978 unclassified Aggregatibacter* | *zOTU_2139 Neisseria flavescens/subflava* |
|  | *zOTU_1080 unclassified Aggregatibacter* | *zOTU_34 Streptococcus gordonii/HOT 056** |
|  | *zOTU_65 Bergeyella HOT 322* | *zOTU_2042 unclassified Streptococcus* |
|  | *zOTU_138 Campylobacter rectus/showae* | *zOTU_997 unclassified Veillonella* |
|  | *zOTU_35 Haemophilus parahaemolyticus/paraphrohaemolyticus/sputorum** | *zOTU_1638 unclassified Veillonella* |
|  | *zOTU_1481 Haemophilus parainfluenzae* | *zOTU_490 Veillonella dispar* |
|  | *zOTU_334 Lachnospiraceae [G-3] HOT 100* | *zOTU_780 Veillonella dispar* |
|  | *zOTU_20 Lautropia mirabilis* | *zOTU_803 Veillonella dispar* |
|  | *zOTU_1692 Lautropia mirabilis* | *zOTU_19 Veillonella parvula* |
|  | *zOTU_189 Leptotrichia HOT 219* |  |
|  | *zOTU_58 Neisseria oralis* |  |
|  | *zOTU_942 Neisseria oralis* |  |
|  | *zOTU_2025 unclassified Neisseria* |  |
|  | *zOTU_2220 unclassified Neisseria* |  |
|  | *zOTU_457 unclassified Propionibacterium* |  |
|  | *zOTU_21 Rothia aeria* |  |
|  | *zOTU_1174 Rothia aeria* |  |
|  | *zOTU_12 Streptococcus australis/parasanguinis I/parasanguinis II/HOT 057/HOT 066* |  |
|  | *zOTU_2336 Streptococcus lactarius/peroris/HOT 074** |  |
|  | *zOTU_302 TM7 [G-1] HOT 348** |  |
|  | *zOTU_291 unclassified Lachnospiraceae [XIV]* |  |
|  | *zOTU_205 unclassified Pasteurellaceae* |  |
|  | *zOTU_242 unclassified Pasteurellaceae* |  |
|  | *zOTU_1257 unclassified Pasteurellaceae* |  |

zOTUs, which significantly contributed to the differences among the caries groups, shown in the columns under the name of the respective caries group.

zOTUs were at a higher proportion in the caries group they are written compared to the other group.

zOTUs are ordered by name of genera in alphabetical order

zOTUs are ordered by name of genera in alphabetical order

* indicates zOTUs additionally blasted on HOMD website with similarity ≥ 98.5%.

| **C** | **Groups compared at T3 in the dental plaque samples** | |
| --- | --- | --- |
|  | **CFAT** | **AC** |
|  | *zOTU_36 Abiotrophia defectiva* | *zOTU_1138 unclassified Actinobacteria* |
|  | *zOTU_73 Aggregatibacter aphrophilus/paraphrophilus* | *zOTU_591 unclassified Actinomyces* |
|  | *zOTU_131 Aggregatibacter HOT 513/HOT 898** | *zOTU_1319 unclassified Actinomyces* |
|  | *zOTU_978 unclassified Aggregatibacter* | *zOTU_1823 unclassified Corynebacterium* |
|  | *zOTU_1080 unclassified Aggregatibacter* | *zOTU_44 Kingella oralis* |
|  | *zOTU_1599 unclassified Aggregatibacter* | *zOTU_2129 Kingella oralis* |
|  | *zOTU_11 Alloprevotella HOT 473* | *zOTU_943 Leptotrichia hongkongensis* |
|  | *zOTU_1724 unclassified Betaproteobacteria* | *zOTU_1165 Leptotrichia hongkongensis* |
|  | *zOTU_138 Campylobacter rectus/showae* | *zOTU_1940 Leptotrichia hongkongensis* |
|  | *zOTU_160 Capnocytophaga HOT 332* | *zOTU_92 Leptotrichia HOT 221* |
|  | *zOTU_171 Capnocytophaga HOT 338* | *zOTU_93 Leptotrichia wadei* |
|  | *zOTU_172 unclassified Capnocytophaga* | *zOTU_1115 unclassified Leptotrichia* |
|  | *zOTU_48 Cardiobacterium hominis* | *zOTU_2389 Neisseria flava/flavescens/mucosa/pharyngis/sicca/subflava** |
|  | *zOTU_14 Corynebacterium durum* | *zOTU_107 Prevotella histicola** |
|  | *zOTU_202 Eikenella corrodens* | *zOTU_541 Prevotella salivae* |
|  | *zOTU_30 Fusobacterium periodonticum* | *zOTU_1374 Porphyromonas pasteri/HOT 278* |
|  | *zOTU_2285 unclassified Fusobacterium* | *zOTU_2268 Rothia dentocariosa* |
|  | *zOTU_1481 Haemophilus parainfluenzae* | *zOTU_201 Stomatobaculum longum** |
|  | *zOTU_35 Haemophilus parahaemolyticus/paraphrohaemolyticus/sputorum** | *zOTU_80 Streptococcus australis/lactarius/parasanguinis I/parasanguinis II/peroris/HOT 057/HOT 066/HOT 074** |
|  | *zOTU_87 Haemophilus pittmaniae** | *zOTU_992 Streptococcus dentisani/infantis/mitis/oralis/HOT 058/HOT 061/HOT 064/HOT 070/HOT 423/HOT 431/tigurinus* |
|  | *zOTU_216 unclassified Haemophilus* | *zOTU_2411 Streptococcus dentisani/infantis/mitis/oralis/HOT 058/HOT 061/HOT 064/HOT 070/HOT 423/HOT 431/tigurinus* |
|  | *zOTU_132 Kingella HOT 012* | *zOTU_47 Streptococcus gordonii/HOT 056** |
|  | *zOTU_22 unclassified Kingella/Neisseria** | *zOTU_1548 Streptococcus gordonii/HOT 056** |
|  | *zOTU_265 unclassified Kingella/Neisseria** | *zOTU_266 Streptococcus intermedius** |
|  | *zOTU_1798 unclassified Kingella/Neisseria** | *zOTU_1519 Streptococcus mutans* |
|  | *zOTU_334 Lachnospiraceae [G-3] HOT 100* | *zOTU_1803 Streptococcus pneumoniae** |
|  | *zOTU_291 unclassified Lachnospiraceae [XIV]* | *zOTU_75 unclassified Streptococcus* |
|  | *zOTU_385 unclassified Lachnospiraceae [XIV]* | *zOTU_1205 unclassified Streptococcus* |
|  | *zOTU_1692 Lautropia mirabilis* | *zOTU_1622 unclassified Streptococcus* |
|  | *zOTU_260 Leptotrichia goodfellowii* | *zOTU_158 unclassified Lactobacillales* |
|  | *zOTU_66 Leptotrichia HOT 212* | *zOTU_353 unclassified Lactobacillales* |
|  | *zOTU_1831 Leptotrichia HOT 212* | *zOTU_490 Veillonella dispar* |
|  | *zOTU_2095 Leptotrichia HOT 212* | *zOTU_1879 Veillonella dispar* |
|  | *zOTU_2113 Leptotrichia HOT 212* | *zOTU_19 Veillonella parvula* |
|  | *zOTU_2403 Leptotrichia HOT 212* | *zOTU_828 unclassified Veillonella* |
|  | *zOTU_189 Leptotrichia HOT 219* | *zOTU_1088 unclassified Veillonella* |
|  | *zOTU_53 Leptotrichia HOT 225* | *zOTU_1179 unclassified Veillonella* |
|  | *zOTU_166 Leptotrichia HOT 225* | *zOTU_1509 unclassified Veillonella* |
|  | *zOTU_1447 Leptotrichia HOT 225* | *zOTU_2146 unclassified Veillonella* |
|  | *zOTU_1689 Leptotrichia HOT 225* | *zOTU_2165 unclassified Veillonella* |
|  | *zOTU_2066 Leptotrichia HOT 225* | *zOTU_2229 unclassified Veillonella* |
|  | *zOTU_497 Neisseria flavescens/subflava* |  |
|  | *zOTU_942 Neisseria oralis* |  |
|  | *zOTU_1070 unclassified Neisseria* |  |
|  | *zOTU_1092 unclassified Neisseria* |  |
|  | *zOTU_1844 unclassified Neisseria* |  |
|  | *zOTU_2025 unclassified Neisseria* |  |
|  | *zOTU_2220 unclassified Neisseria* |  |
|  | *zOTU_1047 unclassified Neisseriaceae* |  |
|  | *zOTU_205 unclassified Pasteurellaceae* |  |
|  | *zOTU_242 unclassified Pasteurellaceae* |  |
|  | *zOTU_1052 unclassified Proteobacteria* |  |
|  | *zOTU_21 Rothia aeria* |  |
|  | *zOTU_979 Rothia aeria* |  |
|  | *zOTU_1174 Rothia aeria* |  |
|  | *zOTU_1662 Rothia aeria* |  |
|  | *zOTU_1892 Rothia aeria* |  |
|  | *zOTU_2350 Streptococcus dentisani/infantis/mitis/oralis/HOT 058/HOT 061/HOT 064/HOT 070/HOT 423/HOT 431/tigurinus* |  |

zOTUs, which significantly contributed to the differences among the caries groups, shown in the columns under the name of the respective caries group.

zOTUs were at a higher proportion in the caries group they are written compared to the other group.

zOTUs are ordered by name of genera in alphabetical order

zOTUs are ordered by name of genera in alphabetical order

* indicates zOTUs additionally blasted on HOMD website with similarity ≥ 98.5%.
